# Cardiovascular screening to reduce the burden from cardiovascular disease: microsimulation study to quantify policy options

**DOI:** 10.1136/bmj.i2793

**Published:** 2016-06-08

**Authors:** Chris Kypridemos, Kirk Allen, Graeme L Hickey, Maria Guzman-Castillo, Piotr Bandosz, Iain Buchan, Simon Capewell, Martin O’Flaherty

**Affiliations:** 1 Department of Public Health and Policy, University of Liverpool, Liverpool L69 3GB, UK; 2 Lancaster Medical School, Lancaster University, Lancaster, UK; 3 Department of Biostatistics, University of Liverpool, Liverpool, UK; 4 Department of Prevention and Medical Education, Medical University of Gdansk, Gdansk, Poland; 5 Farr Institute @ HeRC, University of Manchester, Manchester, UK

## Abstract

**Objectives** To estimate the potential impact of universal screening for primary prevention of cardiovascular disease (National Health Service Health Checks) on disease burden and socioeconomic inequalities in health in England, and to compare universal screening with alternative feasible strategies.

**Design** Microsimulation study of a close-to-reality synthetic population. Five scenarios were considered: baseline scenario, assuming that current trends in risk factors will continue in the future; universal screening; screening concentrated only in the most deprived areas; structural population-wide intervention; and combination of population-wide intervention and concentrated screening.

**Setting** Synthetic population with similar characteristics to the community dwelling population of England.

**Participants** Synthetic people with traits informed by the health survey for England.

**Main outcome measure** Cardiovascular disease cases and deaths prevented or postponed by 2030, stratified by fifths of socioeconomic status using the index of multiple deprivation.

**Results** Compared with the baseline scenario, universal screening may prevent or postpone approximately 19 000 cases (interquartile range 11 000-28 000) and 3000 deaths (−1000-6000); concentrated screening 17 000 cases (9000-26 000) and 2000 deaths (−1000-5000); population-wide intervention 67 000 cases (57 000-77 000) and 8000 deaths (4000-11 000); and the combination of the population-wide intervention and concentrated screening 82 000 cases (73 000-93 000) and 9000 deaths (6000-13 000). The most equitable strategy would be the combination of the population-wide intervention and concentrated screening, followed by concentrated screening alone and the population-wide intervention. Universal screening had the least apparent impact on socioeconomic inequalities in health.

**Conclusions** When primary prevention strategies for reducing cardiovascular disease burden and inequalities are compared, universal screening seems less effective than alternative strategies, which incorporate population-wide approaches. Further research is needed to identify the best mix of population-wide and risk targeted CVD strategies to maximise cost effectiveness and minimise inequalities.

## Introduction

Cardiovascular disease (CVD) is the leading cause of death worldwide.^1^ Furthermore, substantial socioeconomic inequalities have been observed in CVD mortality in England and elsewhere.^2^ ^3^ These inequalities powerfully reflect much greater premature mortality, and hence shorter life expectancy, among the most deprived groups. In England, the current governmental action plan to tackle the burden of CVD includes a programme known as NHS (National Health Service) Health Checks. Introduced in 2009, this programme promotes the screening of all healthy adults aged 40 to 74 for CVD risk stratification, and treatment of those at high risk.^4^ ^5^ Recently, the debate about the programme’s scientific foundation, effectiveness, and cost effectiveness, however, has been heated.^6^ ^7^ ^8^ ^9^ ^10^ Despite the controversy, the programme remains policy.

Beyond the obvious importance of the debate to national public health, the programme’s relevance extends internationally. Choices about public health policy in the United Kingdom influence policy worldwide; the UK policies on tobacco control and salt reduction are two recent examples.^11^ ^12^ In essence, the debate about NHS Health Checks originates from the archetypal debate of targeted “high risk” versus “population-wide” preventive interventions that was first articulated by Geoffrey Rose.^13^ Rose argued that population-wide interventions are more effective than ones aimed at high risk groups because the majority of incident cases occur in the multitudinous group of people at low and intermediate risk. In Rose terminology, NHS Health Checks is a typical “high risk intervention,” as it targets people at high risk rather than lowering risk in the whole population.

The effectiveness of high risk interventions for CVD prevention has been previously challenged.^14^ More recently, a Cochrane systematic review and the Inter99 trial found no benefits of health checks on CVD morbidity or mortality.^15^ ^16^ There were, however, major limitations to these studies: Inter99 trialled a counselling intervention not supported by additional drug treatment, and in the Cochrane review nine out of 14 trials were conducted before 1980, when the treatment options for high risk people were limited. In addition, high risk interventions may be more effective in populations with high clustering of risk factors, resulting in a high concentration of the risk to certain groups in the population.^17^ In fact, the English population has such characteristics, with the risk of CVD being higher among those in the most socioeconomically deprived groups.^18^

High risk interventions may generate health inequalities because they require active participation of people in both screening and treatment of those at high risk, favouring those with more resources.^14^ ^19^ ^20^ ^21^ The particular effect of NHS Health Checks on socioeconomic health inequalities remains unclear however. A national study reported no difference in the coverage of the intervention by deprivation,^22^ whereas several smaller, but more detailed, studies showed substantially lower uptake in deprived areas.^23^ ^24^ ^25^

We estimated the potential impact of universal screening for primary prevention of CVD on disease burden and socioeconomic health inequalities in England. Available data on the effectiveness of the NHS Health Check programme have been used to model this scenario. We further compared universal CVD screening with an alternative approach targeting only deprived areas, a feasible population-wide intervention, and a combination of both.

## Methods

Building on experience from the original, validated IMPACT model^26^ and the more recent IMPACT_SEC_^27^ and IMPACT2 models,^28^ we created IMPACT_NCD_, a discrete time dynamic stochastic microsimulation model. IMPACT_NCD_ simulates the life course of synthetic individuals under different counterfactual scenarios, up to 2030 (the projection horizon). During the simulation, CVD incidence and CVD and non-CVD mortality are recorded. The results are stratified by year, five year age group, sex, and fifths of index of multiple deprivation. The last is a relative measure of area deprivation that is widely used by public health authorities in England, and it has been used as the measure of socioeconomic classification for this study.^29^

A more detailed description of the model is provided in the supplementary material and the source code is available at https://github.com/ChristK/IMPACTncd/tree/CVD-policy-options.

### Scenarios

We considered five scenarios.

#### Baseline (current trends)

In the baseline scenario, we assumed that the recent observed trends in CVD risk factor trajectories by age, sex, and socioeconomic status will continue in the near future. We extracted the trends from the health survey for England 2001-12, a nationally representative series of health surveys conducted in England annually.^30^ ^31^ ^32^ ^33^ ^34^ ^35^ ^36^ ^37^ ^38^ ^39^ ^40^ ^41^ ^42^

#### Universal screening

This scenario modelled the potential health effects of universal screening to identify and treat people at high risk for CVD. Input variables were informed from current implementation of the NHS Health Check programme. Eligible people were defined as adults aged between 40 and 74, excluding those with a known history of CVD, atrial fibrillation, diabetes mellitus, rheumatoid arthritis, or renal disease; closely resembling real life eligibility criteria. Based on existing evidence we assumed an uptake of 50% for screening,^43^ and we calibrated the distribution of the estimated 10 year risk of developing CVD among those participating: 70% with a less than 10% risk, 25% with between 10% and 20%, and 5% with more than 20%.^22^ In addition, we calibrated the age distribution so that around 30% of those screened were older than 60.^22^ Participants with a higher than 10% estimated 10 year risk of developing CVD were considered at high risk and eligible for treatment. We used the QRISK2 score to estimate the 10 year risk of developing CVD, as perceived from healthcare.^44^

Based on published evidence, we assumed that about 24% with an estimated risk of 20% or more and total cholesterol of 5 mmol/L or more will be prescribed atorvastatin 20 mg and about 27% with an estimated risk of 20% or more and a systolic blood pressure of 135 mm Hg or more will be prescribed antihypertensive drugs. For those with a risk between 10% and 20% we assumed that about 17% and 20% will be prescribed treatment, respectively.^45^ We assumed an 80% persistence with treatment and a mean adherence of approximately 70%, roughly based on evidence from Denmark.^46^ Moreover, we modelled high risk participants with a body mass index of more than 50 kg/m^2^ to undergo bariatric surgery and reduce their body mass index to 30 kg/m^2^. We assumed that with lifestyle counselling half of the high risk participants consuming fewer than five fruit and vegetable portions daily will increase their consumption by a portion daily. Half of those being active for less than five days a week will increase their physical activity by an active day each week, and all high risk participants will decrease their body mass index by around 1%.^45^ ^47^ Finally, we modelled 10% of high risk smokers to achieve cessation for a year and have a probability of relapse equal to that of the general population by sex, fifth of multiple deprivation, and years since cessation.^48^ ^49^

#### Concentrated screening

In the concentrated screening scenario, we simulated a hypothetical strategy where screening had only been implemented in the most deprived fifths (groups 4 and 5), the groups with the greatest concentration of CVD risk. We assumed that the uptake of the intervention was 50% and the risk and age distribution in the participants was similar to that in the eligible population. Otherwise, the strategy is similar to the previous universal screening scenario. Given the recent criticism about the cost and cost effectiveness of the intervention,^9^ offering the intervention where the risk is more concentrated may reduce costs.

#### Population-wide intervention

This scenario modelled the effects of a feasible population-wide structural intervention targeting unhealthy diet and smoking. Several studies have found that a tax on sugar sweetened beverages may reduce the prevalence of obesity.^50^ ^51^ ^52^ For this scenario we assumed that such a tax may reduce the mean increase in body mass index by about 5% annually. Moreover, the United Kingdom has had one of the world’s most successful salt reduction strategies, including public awareness campaigns, food labelling, and voluntary reformulation of processed foods.^53^ Modelling studies suggested that the addition of mandatory reformulation of processed foods may further reduce mean systolic blood pressure by 0.8 mm Hg^54^; we modelled this decrease. A large randomised trial in the United States showed that subsidies on fruits and vegetables may increase consumption by about half a portion daily, and a modelling study in the UK found that subsidising fruits and vegetables combined with taxation of unhealthy foods may increase fruit and vegetable annual consumption by about 10%.^55^ ^56^ We modelled an increase of a portion of fruit and vegetable each day in 50% of the population. Finally, a SimSmoke modelling study estimated that full compliance with the framework convention on tobacco control may reduce smoking prevalence by 13% (relative) in five years^57^; we modelled this decrease.

#### Population-wide intervention and concentrated screening

This scenario is the combination of the population-wide intervention and concentrated screening strategies. We modelled the implementation of a population-wide strategy identical to the previous scenario, complemented by concentrated screening for people at high risk of CVD in the most deprived fifths (groups 4 and 5).

#### Common scenario assumptions

All interventions begun in 2011 and were linearly diffused into the population over a five year period. Trends in population risk factors were assumed to be the same as those of the baseline scenario for all but the population-wide intervention. All of the scenarios assumed that CVD case fatality will keep improving by 3% (relative) annually. In addition, we assumed a socioeconomic gradient in CVD case fatality, forcing the more deprived people to experience worse outcomes. Both case fatality assumptions were based on recent trends and are supported by the British Heart Foundation’s statistics on coronary heart disease.^2^ Finally, a five year lag time was assumed between exposure to cardiovascular risk factors and disease.

### Model description

#### Inputs and logic

IMPACT_NCD_ synthesises information from the Office for National Statistics and the health surveys for England on the English population’s demographics and its exposure to CVD associated risk factors, to generate a close-to-reality synthetic population.^58^ Well established causal pathways between CVD and the associated risk factors are used to translate exposure into CVD incidence and mortality, in a competing risk framework. We obtained effect sizes for exposures from published meta-analyses and longitudinal studies (see supplementary table S1).

The risk factors we considered for this study were age, sex, fifth of deprivation, body mass index, systolic blood pressure, total cholesterol level, diabetes mellitus (diagnosis or increased glycated haemoglobin level/no diabetes), smoking status (current, former, or never smoker), environmental tobacco exposure (binary variable), fruit and vegetable consumption (portions daily), and physical activity (days with at least 30 minutes of moderate or vigorous physical activity each week). CVD was defined as the sum of coronary heart disease and stroke (any type) cases. As this study focuses on primary prevention, we considered only the first ever episode of coronary heart disease or stroke. The competing risk framework allowed people to develop coronary heart disease and/or stroke separately, and to die from these two diseases or any other cause.

#### Model outputs

We report the cumulative estimates of cases and deaths prevented or postponed as measures of overall effectiveness of the modelled interventions. To measure the impact of the modelled interventions on absolute and relative socioeconomic health inequalities, we developed and used two regression based metrics inspired by the slope index of inequality^59^; the absolute equity slope index and the relative equity slope index. The absolute equity slope index measures the impact of an intervention on absolute inequality; for example, a value of 100 means 100 more cases were prevented or postponed in most deprived areas compared with least deprived areas, resulting in a decrease in absolute inequality. The relative equity slope index takes into account the pre-existing socioeconomic gradient of disease burden and measures the impact of an intervention on relative inequality. Positive values mean the intervention tackles relative inequalities and negative values that the intervention generates relative inequality. Finally, we summarised the overall impact of each scenario on CVD burden and equity in the equity summary chart.

### Uncertainty and sensitivity analysis

IMPACT_NCD_ implements a second order Monte Carlo design that allows uncertainty to be quantified from the outputs. We used distributions to model the uncertainty around all scenario specific inputs and the sampling error of the risk associated with the CVD related risk factors. The probabilistic sensitivity analysis has been incorporated in our estimates. We summarise the distributions by reporting medians and interquartile ranges in the form of first and third fourths. The supplementary file provides a more detailed description of the sources of uncertainty and the relevant distributions.

We ran three further scenarios offering slight variations on the two primary ones of universal screening and population-wide intervention: a universal screening variation, where we assumed a treatment threshold recommendation of 20% risk instead of 10%; another variation on universal screening, where we assumed a socioeconomic differential in screening uptake, with the most deprived of the population to be 10% less likely to participate; and a variation on the population-wide intervention, where we only modelled dietary interventions, excluding smoking interventions. The supplementary file provides detailed information on the extra scenarios.

### Validation

We assessed the predictive validity of the IMPACT_NCD_ model by comparing the estimated number of deaths from CVD with the observed number of deaths from the same causes for 2006 to 2013 in England.^60^ We further compared the IMPACT_NCD_ output with CVD mortality forecasts from a bayesian age-period-cohort model.^61^

### Patient involvement

No patients were involved in setting the research question or the outcome measures, nor were they involved in developing plans for design or implementation of the study. No patients were asked to advise on interpretation or writing up of results. There are no plans to disseminate the results of the research to study participants or the relevant patient community.

## Results

IMPACT_NCD_ outputs for CVD burden and inequality are summarised for ages 30 to 84. Because of the assumed five year time lag, the interventions affect the population from 2016 up to the projection horizon of 2030. The impact of the five scenarios on risk factor trajectories are further illustrated in additional graphs in the supplementary file.

### Overall effectiveness

Under the baseline scenario, IMPACT_NCD_ estimated about 1.4 million (interquartile range 1.3-1.5) cases of CVD and 540 000 deaths (interquartile range 520 000 to 550 000) between 2016 and 2030. The most effective intervention was the combination of the population-wide intervention and concentrated screening. The population-wide intervention alone had the second highest effectiveness, whereas the universal and the concentrated screening scenarios were considerably less effective (table 1). Despite the improvement of most CVD related risk factors, the proportion of high risk people in the eligible population is slowly increasing over time, because of population aging (fig 1).

**Table 1 tbl1:** Estimated cases and deaths prevented or postponed under each scenario, by 2030

Scenarios	No (interquartile range) prevented or postponed	
	Cases	Deaths
Universal screening	19 000 (11 000-28 000)	3000 (−1000-6000)
Concentrated screening	17 000 (9000-26 000)	2000 (−1000-5000)
Population-wide intervention	67 000 (57 000-77 000)	8000 (4000-11 000)
Population-wide intervention and concentrated screening	82 000 (73 000-93 000)	9000 (6000-13 000)

Results rounded to nearest 1000.

**Figure f1:**
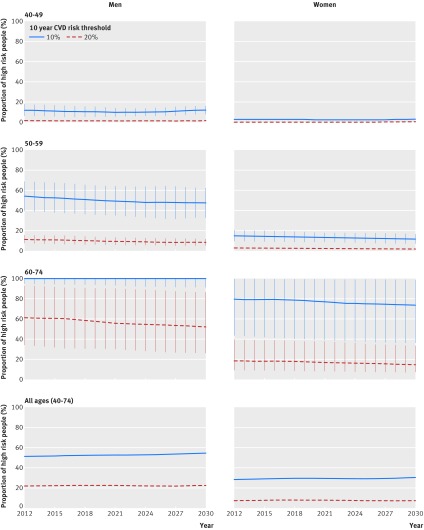
**Fig 1** Proportion of high risk people eligible for universal screening population projections, by age group and sex. 10 year risk of cardiovascular disease (CVD) was estimated from QRISK2 score. Error bars represent interquartile ranges

### Socioeconomic inequalities

When socioeconomic inequalities were considered, the patterns for reductions in absolute and relative inequalities were similar. The combination of the population-wide intervention and concentrated screening seemed the most powerful among the simulated interventions (tables 2 and 3[Table tbl2 tbl3]). Concentrated screening alone was the second most powerful intervention in tackling inequalities, followed by the population-wide intervention. Finally, universal screening of CVD is likely to have a small, if any, effect on socioeconomic inequalities.

**Table 2 tbl2:** Cases prevented or postponed according to fifth of deprivation by 2030, along with absolute equity slope index for each scenario

Deprivation fifth*	No (interquartile range) of cases prevented or postponed			
	Universal screening	Concentrated screening	Population-wide intervention	Population-wide intervention+concentrated screening
First (least deprived)	3400 (−1400-8300)	0	10 800 (5900-15 500)	10 800 (6200-15 700)
Second	2900 (−1500-8400)	0	12 200 (6200-17 200)	11 500 (6600-17 000)
Third	4000 (−900-9300)	0	13 100 (8100-18 300)	12 600 (7400-17 700)
Fourth	3700 (−1600-8600)	6400 (1500-11 800)	12 500 (7100-18 400)	18 700 (13 900-24 200)
Fifth (most deprived)	4900 (−600-10 400)	10 700 (5300-16 300)	18 700 (13 000-24 000)	28 600 (22 800-33 200)
Absolute equity slope index	1700 (−6200-9300)	14 100 (5700-23 000)	8400 (−400-16 900)	21 100 (12 800-29 300)

Results rounded to nearest 1000.

*According to index of multiple deprivation.

**Table 3 tbl3:** Relative percentage reduction in cases of cardiovascular disease according to fifth of deprivation by 2030, along with relative equity slope index for each scenario

Deprivation fifth*	Relative % reduction (interquartile range)			
	Universal screening	Concentrated screening	Population-wide intervention	Population-wide intervention+concentrated screening
First (least deprived)	1.3 (−0.5-3.1)	0	4.1 (2.2-5.9)	4.0 (2.4-6.0)
Second	1.1 (−0.5-2.9)	0	4.2 (2.2-5.9)	4.0 (2.3-5.9)
Third	1.4 (−0.3-3.2)	0	4.6 (2.8-6.3)	4.4 (2.6-6.2)
Fourth	1.3 (−0.6-3.1)	2.4 (0.6-4.3)	4.6 (2.7-6.6)	6.9 (5.1-8.9)
Fifth (most deprived)	1.6 (−0.2-3.3)	3.6 (1.8-5.3)	6.2 (4.4-8.0)	9.4 (7.6-11.2)
Relative equity slope index	0.4 (−2.4-3.2)	4.9 (1.8-7.9)	2.3 (−0.7-5.3)	6.7 (3.8-9.5)

Results rounded to one decimal place.

*According to index of multiple deprivation.

### Equity summary chart

We summarised our estimates for the effectiveness and equity of the modelled interventions in the equity summary chart (fig 2). The horizontal axis of the chart represents the cases of CVD prevented or postponed and the vertical axis the reduction in absolute inequality. Scenarios above the equity curve (dashed curve in the figure) decrease relative socioeconomic inequality, and scenarios below the curve increase it. The vertical distance from the curve approximates the impact of the scenario on relative inequality. (See the supplementary file for more details about this chart.) The combination of the population-wide intervention and concentrated screening is by far the most effective and equitable intervention. Concentrated screening is also equitable but with few mortality gains.

**Figure f2:**
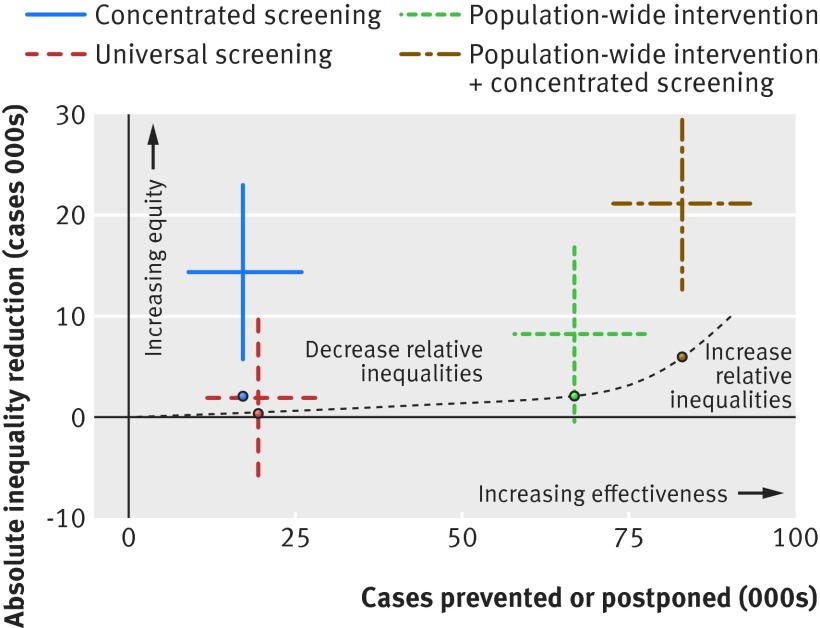
**Fig 2** Equity summary chart of effectiveness and equity of all modelled interventions, compared with baseline scenario (beginning of axes). Dashed line represents “equity” curve. Interventions below the curve increase relative inequality, whereas interventions above it decrease relative inequalities. Smaller coloured dots represent reference points used to fit equity curve. Horizontal and vertical error bars represent interquartile ranges

### Sensitivity analysis

Adding assumptions to extend the scenarios did not displace our main findings. The three most notable results of the sensitivity analysis were:

Raising the treatment threshold from 10% to 20% further reduced the effectiveness of universal screening by about 60% in preventing CVD cases. However, in preventing deaths from CVD the effectiveness decreased by only 15% as raising the treatment threshold excludes younger participants at intermediate risk from treatment.

Assuming a differential uptake of universal screening by deprivation fifth essentially eliminated the estimated small potential benefit of universal screening in tackling health inequalities.

A population-wide intervention targeting only diet would still be about twice as effective as universal screening and more than twice as effective as population-wide intervention targeting smoking alone—so the relative ranking of scenario effectiveness would remain unaltered. For detailed results see supplementary tables S11-S13.

### Validation

We assessed the predictive validity of the IMPACT_NCD_ model by comparing the estimated number of deaths from CVD with the observed number of deaths from the same cause for 2006 to 2013 in England (fig 3). See the supplementary file for detailed graphs by age group, sex, deprivation fifth, and disease.

**Figure f3:**
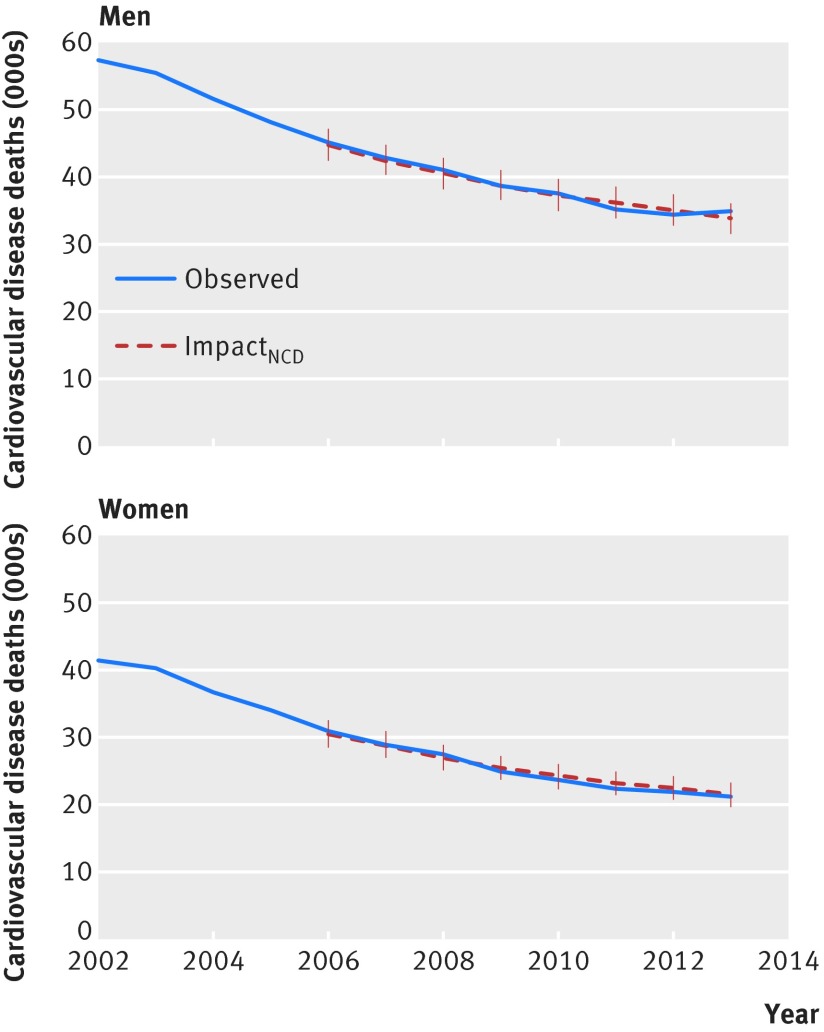
**Fig 3** Number of deaths from cardiovascular disease (CVD) in England, by year for ages 30 to 84. Office for National Statistics reported deaths (observed) versus IMPACT_NCD_ estimated. Observed deaths after 2010 were adjusted to account for changes in ICD-10 version used by the Office for National Statistics from 2011 onwards. Error bars represent interquartile ranges

## Discussion

Our results strongly suggest that universal screening and treatment of people at high risk is not the most effective option for primary prevention of cardiovascular disease (CVD) overall, nor for reducing socioeconomic inequalities. In contrast, prevention strategies that include population-wide structural interventions seem to be the consistently better options for reducing overall CVD burden and inequalities. This echoes and quantifies findings from other, mostly theoretical, studies supporting that structural population-wide interventions are powerful, while reducing socioeconomic health inequalities.^13^ ^14^ ^62^ ^63^ Indeed, the impact of the population-wide intervention scenario on reduction in estimated mortality and inequalities seems compatible with previous estimates, considering the different methodologies.^64^ Furthermore, the effectiveness and equity of population-wide structural interventions can be further improved by the addition of targeted interventions in the most deprived groups, as highlighted in the combined scenario of the population-wide intervention and concentrated screening.

Compared with other modelling approaches, our IMPACT_NCD_ model estimated that NHS Health Checks might prevent approximately 1000 non-fatal and 200 fatal cases of CVD annually. This is comparable with the Department of Health estimates of 1600 non-fatal CVD cases and 650 deaths prevented annually.^4^ Furthermore, the Department of Health modelling approach assumed an intervention uptake of 75%; higher than the current observed levels. Using the Archimedes model, Schuetz et al estimated that health checks in the UK could prevent some 12 CVD cases per 1000 population screened after 30 years’ follow-up^65^ (7500 CVD cases prevented each year extrapolating to the eligible English population). That higher estimate reflects the researchers’ apparently unrealistic assumption of 100% screening uptake and 50% overall uptake of treatment.

### The scenarios

We modelled the universal screening scenario to closely resemble the current implementation of the NHS Health Check programme, based on published evidence. Therefore, we maintain that our estimates on the effectiveness of this scenario are not far from the real world effectiveness of NHS Health Checks. However, our output suggesting that universal screening might reduce socioeconomic inequalities seems to contradict existing empirical and modelling evidence.^14^ ^19^ ^20^ ^21^ This is because we generously assumed identical screening uptake and treatment adherence for all socioeconomic groups. In fact, any potential reduction in socioeconomic health inequalities was essentially eliminated when we considered a small socioeconomic differential in uptake in the sensitivity analysis. Furthermore, additional health inequalities may arise from differential persistence and adherence to treatment by deprivation status.^46^

The population-wide intervention scenario on the other hand, is based mostly on structural policies targeting price and availability. This scenario potential effectiveness was mostly based on natural experiments,^66^ ^67^ and on previous modelling studies from the UK and elsewhere. The size of the changes in the population risk factors that we modelled were modest, and actually smaller than the reductions observed in countries such as France, Finland, and the USA during recent decades.^68^ ^69^ ^70^ This scenario estimated the reduction in mortality conservatively, because it ignored the beneficial effect of the policies on survival from CVD. Similarly, it underestimated the reduction of the gap in inequalities, because it did not fully consider the current disproportionate burden of poor diet among the most deprived of the population,^71^ and hence the potential for improvement through population-wide policies.

Finally, the concentrated screening strategy was the weakest in terms of overall effectiveness, yet more powerful in tackling inequalities. Its increased impact on socioeconomic health inequalities is a direct consequence of the concentrated prevention only to the more deprived quantiles of the population. However, the scenario assumptions may not fully hold in real world implementation. Hence, concentrated screening represents a challenge for public health practitioners and policymakers to exploit the opportunity of a smaller and more homogeneous eligible population and to implement better recruitment and tactics for treatment adherence. Yet, cost effectiveness might also fall because of loss of economies of scale.

### Public health implications

This IMPACT_NCD_ modelling may help stakeholders to understand better the interplay between preventive policies, risk factors, disease, and inequalities, and thus potentially inform health policy and strategy. Hence, when compared with the alternative feasible interventions, universal screening seemed inferior both in primary prevention and in reducing socioeconomic health inequalities. Additionally, we estimated that the proportion of young people at high risk aged less than 60 in the eligible population will decrease in future (fig 1). This will render universal screening less effective and less cost effective for this age group, because a larger number will need to be screened to identify each high risk individual.

Our study suggests that despite the high clustering of risk factors in the most deprived parts of the population, structural population-wide approaches remain more effective than high risk ones for the prevention of CVD. Population-wide approaches also seem to be more effective in reducing absolute and relative socioeconomic health inequalities, generally cost much less than a universal screening programme, and may even be cost saving.^72^ ^73^ In this study, we did not model the full potential of these policies, as we focused only on diet and smoking interventions; we did not, for example, incorporate alcohol consumption or physical activity. In addition, we did not simulate the likely wider benefits of improved diet and smoking cessation on the plethora of relevant non-communicable diseases. Despite this restricted scope, for CVD prevention we estimated that structural policies targeting diet could be twice as effective as those targeting smoking. Yet, structural interventions for a healthier diet are currently underutilised compared with tobacco control. Several countries have now introduced taxes on sugary drinks or sugar, including Finland, France, Latvia, and Mexico. The UK has recently followed their example. Hungary is the only European country currently taxing unhealthy “junk” food.^74^ However, fiscal interventions may face opposition from commercial vested interests.^75^ Interestingly, an increasing body of evidence from empirical studies and modelling analyses suggest that the maximum health impact with a neutral effect on poverty may occur when food or drinks taxes are combined with subsidies for healthy foods.^56^ ^76^ ^77^

Moreover, the combination of a population-wide intervention with an intervention targeting the most deprived members, may further improve effectiveness and equity. This approach is in the spirit of proportionate universalism that was identified in the Marmot review as the best approach to tackle socioeconomic inequalities in health.^78^ Our study provides evidence that in CVD prevention proportionate universalism may be the best option not only for tackling inequalities but also for overall effectiveness.

### Strengths and limitations of this study

IMPACT_NCD_ is the first microsimulation model to synthesise core principles of social and CVD epidemiology, vital demographics, published literature, and recent health surveys for England to create a synthetic population of England, including socioeconomic structure, at the individual level. The microsimulation approach allows for the simulation of detailed scenarios and explores the distributional nature of their impact on the population, in a competing risks framework. Microsimulation allows for greater flexibility and more detailed simulation, demanding more statistical and computational resources than older approaches; we utilised the Farr Institute’s statistical high performance computing facilities.^79^ Many assumptions must be made with such models. Yet, despite the potential frailty of such assumptions, this model validated well against observed CVD mortality, even when multiply stratified. Finally, to ensure transparency, we have made the IMPACT_NCD_ source code open under GNU GPLv3 license.

Models are simplifications of reality and thus possess inherent limitations. At least four items were not included in the current model. Firstly, the multiplicative risk assumption is considered the status quo in comparative risk assessments^80^; however, this may oversimplify the complex nature of interactions between multiple risk factors and disease outcome over the life course. Secondly, IMPACT_NCD_ currently ignores the effect of risk factors on CVD case fatality, although in this study we considered only primary prevention scenarios. Thirdly, complex population dynamics such as migration, social mobility, and the socioeconomic consequences of disease were not modelled. We consider this bias would be relatively small for projections with a short horizon. Fourthly, the model ignores the impact of universal screening in recognising previously undiagnosed cases of atrial fibrillation and other opportunistic diagnoses. Reassuringly, most of these biases apply across all scenarios; their effects would thus be reduced in comparisons between scenarios.

### Conclusions

When comparing primary prevention strategies for reducing CVD burden and inequalities, universal screening seems less effective than alternative strategies that incorporate population-wide approaches. Further research is needed to identify the best mix of population-wide and risk targeted CVD strategies to maximise cost effectiveness and minimise inequalities.

**What is already known on this topic**

Two main strategies for the primary prevention of cardiovascular disease (CVD) is to screen the population, find those individuals at high risk, and treat them or to reduce the CVD risk of the whole population irrespective of individuals’ baseline risk
Evidence suggests that the second approach is more effective and likely more equitable, yet this depends on the distribution of CVD risk throughout the population
In England, the Department of Health adopted the first approach, although this decision has recently attracted some criticism

**What this study adds**

In England, despite the observed higher concentration of CVD risk in more deprived areas, structural population-wide interventions targeting unhealthy diet and tobacco might be three times more effective than the existing screening policy
Structural population-wide interventions are also likely to be more equitable than screening
A comprehensive strategy, combining structural population-wide interventions with screening in the most deprived areas (where CVD risk is concentrated) is most likely to maximise both effectiveness and equity of primary CVD prevention

## Web extra

Extra material supplied by authors

## Notes

We thank Julia Critchley for her valuable advice about modelling physical activity, and the reviewers and editorial committee for their comments and suggestions, which have greatly improved the original manuscript.

Contributors: All authors made substantial contributions to the conception and design of the study. CK and MOF had the original idea. CK prepared and conducted data analysis. All authors drafted and critically revised the manuscript. All authors, external and internal, had full access to all of the data (including statistical reports and tables) in the study and can take responsibility for the integrity of the data and the accuracy of the data analysis. CK is the guarantor of this study.

Ethical approval: Not required as this study is an analysis of previously collected data. Ethical approval for each survey was obtained by the health survey for England team.

Data sharing: Anonymised, non-identifiable participant level cross sectional survey data are freely available for academic researchers and public health staff to download from the UK data service (www.ukdataservice.ac.uk). The source code for IMPACT_NCD_ is available at https://github.com/christk/impactncd/tree/cvd-policy-options.

Transparency: The lead author (the manuscript’s guarantor) affirms that this manuscript is an honest, accurate, and transparent account of the study being reported; that no important aspects of the study have been omitted; and that any discrepancies from the study as planned have been explained.
