# Supplementary material for: Cardiovascular screening to reduce the burden from cardiovascular disease: microsimulation study to quantify policy options
Source: BMJ. 2016 Jun 8;353:i2793. doi: 10.1136/bmj.i2793 (PMC4898640; doi:10.1136/bmj.i2793)
Supplement: Supplementary file 1 — Appendix: supplementary information [file kypc031638.ww1_default.pdf]

Supplementary Appendix

This appendix has been provided by the authors to give readers additional information about their work.

Supplement to: Is cardiovascular screening the best option for reducing future cardiovascular disease burden? A microsimulation study to quantify the policy options.

Table of Contents

Chapter 1. High level description of $IMPACT_{ncd}$ 5

Technical requirements 5

Chapter 2. Population module 7

Estimating exposure to risk factors (steps 2-3) 7

Generating the 'close to reality' synthetic population for $IMPACT_{NCD}$ 7

$IMPACT_{NCD}$ implementation of individualised risk factor trajectories 9

Lag times 13

Birth engine (Step 4) 13

Chapter 3. Disease module 14

Estimating the annual individualised disease risk and incidence (Step 5) 14

Stage 1 14

Stage 2 15

Stage 3 15

Simulating disease histories (Step 6) 16

Simulating mortality (Step 7) 16

Chapter 4. Scenarios 18

Universal screening 18

Population-wide intervention 20

Risk factors trajectories 20

Chapter 5. Sensitivity analysis 29

Chapter 6. Uncertainty 30

Chapter 7. Equity metrics 32

Absolute and relative equity slope index 32

Equity summary chart 33

Chapter 8. Validation 36

Synthetic population validation 36

Risk factor trends validation 45

Mortality concurrent and predictive validation........56

Tables........65

References........82

List of tables

Table S1 IMPACT$_{NCD}$ data sources. 64
Table S2 IMPACT$_{NCD}$ main assumptions and limitations 67
Table S3 Coefficients of the model for fruit and vegetable consumption per day predictions. Brackets contain 95% confidence intervals for the coefficients 68
Table S4 Coefficients of the model for active days per week predictions. Brackets contain 95% confidence intervals for the coefficients 69
Table S5 Coefficients of the model for smoking initiation predictions. Brackets contain 95% confidence intervals for the coefficients 70
Table S6 Coefficients of the model for smoking cessation predictions. Brackets contain 95% confidence intervals for the coefficients 70
Table S7 Coefficients of the model for smoking relapse predictions. Brackets contain 95% confidence intervals for the coefficients 71
Table S8 Coefficients of the model for body mass index predictions. Brackets contain 95% confidence intervals for the coefficients 71
Table S9 Coefficients of the model for systolic blood pressure predictions. Brackets contain 95% confidence intervals for the coefficients 72
Table S10 Coefficients of the model for total cholesterol predictions. Brackets contain 95% confidence intervals for the coefficients 73
Table S11 Estimated cases and deaths prevented or postponed under each scenario, by 2030. Brackets contain the respective interquartile ranges (IQR). 73
Table S12 Cases prevented or postponed per quintile groups of the index of multiple deprivation (QIMD), by 2030. The absolute equity slope index for each scenario is also presented. Brackets contain the respective interquartile ranges (IQR). 74
Table S13 Relative percentage reduction in cardiovascular disease cases per quintile groups of the index of multiple deprivation (QIMD), by 2030. The relative equity slope index for each scenario is also presented. Brackets contain the respective interquartile ranges (IQR). 74
Table S14 Distributions that were used as inputs for the simulations. Numbers are rounded 75

CHAPTER 1. HIGH LEVEL DESCRIPTION OF IMPACT$_{NCD}$

IMPACT$_{NCD}$ is a discrete time dynamic stochastic microsimulation model.[1,2] Within IMPACT$_{NCD}$ each unit is a synthetic individual and is represented by a record containing a unique identifier and a set of associated attributes.

For this study we considered age, sex, quintile groups of index of multiple deprivation (QIMD)[*], body mass index (BMI), systolic blood pressure (SBP), total plasma cholesterol (TC), diabetes mellitus[†] (DM, binary variable), smoking status (current/ex/never smoker), environmental tobacco exposure (ETS, binary variable), fruit and vegetable (F&V) consumption and physical activity[‡] (PA) as the set of associated attributes. A set of stochastic rules are then applied to these individuals, such as the probability of developing coronary heart disease (CHD) or dying, as the simulation advances in discrete annual steps. The output is an estimate of the burden of CHD and stroke in the synthetic population including both total aggregate change and, more importantly, the distributional nature of the change. This allows, among others, for an investigation of the impact of different scenarios on social equity.

IMPACT$_{NCD}$ is a complex model that simulates the life course of synthetic individuals and consists of two modules: The ‘population’ module and the ‘disease’ module. Figure S1 highlights the steps of the algorithm that generate the life course of each synthetic individual. We will fully describe IMPACT$_{NCD}$ by describing the processes in each of these steps in the following chapters. The description is from an epidemiological rather than technical perspective. The source code and all parameter input files are available in https://github.com/ChristK/IMPACTncd/tree/CVD-policy-options under the GNU GPLv3 licence. Tables S1 and S2 summarise the sources of the input parameters and the main assumptions and limitations, respectively.

Technical requirements

IMPACT$_{NCD}$ is being developed in R v3.2.0[4] and is currently deployed in an 80 logical core server with 2TB of RAM running Scientific Linux v6.2. IMPACT$_{NCD}$ is built around the R package ‘data.table’[5], which imports a new heavily optimised data structure in R. Most functions that operate in a data table have been coded in C to improve performance. Each iteration for each scenario is running independently in one of the CPU cores and the R package ‘foreach’[6] is responsible for the distribution of the jobs and collection of the results. To ensure statistical independence of the pseudo-random number generators

[*] QIMD is a measure of relative area deprivation based on the 2010 version of the Index of Multiple Deprivation.[3]

[†] We defined as diabetics those with self-reported medically diagnosed diabetes (excluding pregnancy-only diabetes) or glycated haemoglobin (HbA1c) ≥ 6.5.

[‡] Measured as days per week with 30 or more minutes of moderate or vigorous activity.

running in parallel, the R package ‘doRNG’[7] was used to produce independent random steams of numbers, generated by L'Ecuyer's combined multiple-recursive generator.[8]

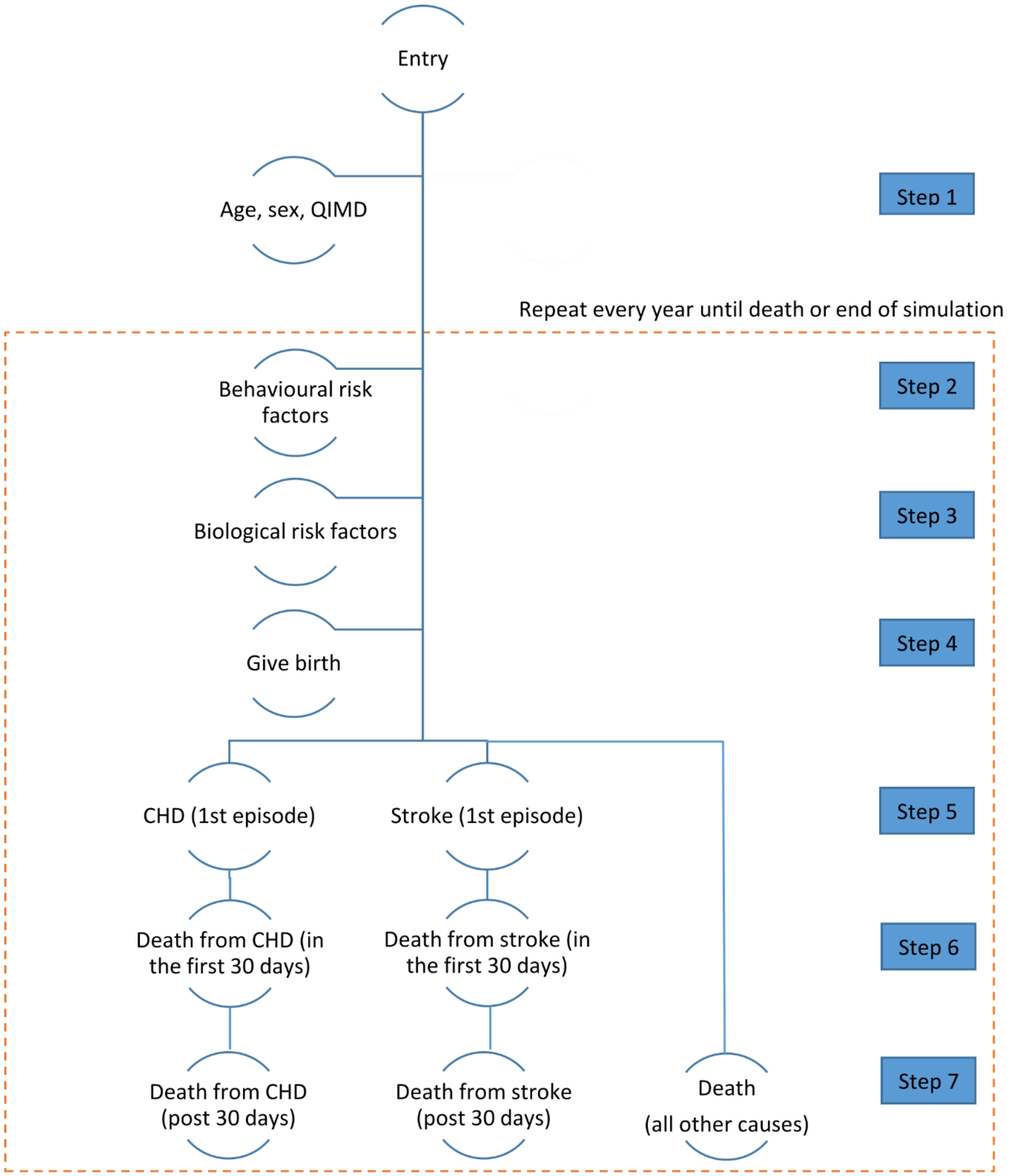

Figure S1 Simplified IMPACT$_{NCD}$ algorithm for individuals. For each step, the algorithm uses information from all appropriate previous steps. CHD denotes coronary heart disease.

Population module

The 'population' module consists of steps 1 to 4 in Figure S1. Synthetic individuals enter into the simulation in the initial year (2011 for this study). The number of synthetic individuals that enter into the simulation is user defined and for this study was set to 400,000. The algorithm ensures that the age, sex and QIMD distribution of the sample is similar to this of the English population in mid-2011. This concludes step 1, which only happens at the beginning of each simulation. Steps 2-7 are calculated annually (in simulation time) for each synthetic individual until the simulation horizon is reached, or death occurs.

Estimating exposure to risk factors (steps 2-3)

In steps 2 and 3, $\text{IMPACT}_{\text{NCD}}$ estimates the exposure of the synthetic individual to the modelled risk factors. It is essential the risk profile of each synthetic individual to be similar to the risk profiles that can be observed in the real English population. For this, we first built a 'close to reality' synthetic population of England from which we sampled the synthetic individuals. Then, we used generalised linear models (GLM) for each modelled risk factor, to simulate individualised risk factor trajectories for all synthetic individuals.

Generating the 'close to reality' synthetic population for $\text{IMPACT}_{\text{NCD}}$

The 'close to reality' synthetic population ensures that the sample of synthetic individuals for the simulation is drawn from a synthetic population similar to the real one in terms of age, sex, socioeconomic circumstance, and risk factors conditional distributions. In our implementation we used the same statistical framework originally developed by Alfons et al.[9] and adapted it to make it compatible with epidemiological principles and frameworks.

In general, this method uses a nationally representative survey of the real population to generate a 'close to reality' synthetic population. Therefore, the method expands the, often small, sample of the survey into a significantly larger synthetic population, while preserves the statistical properties and important correlations of the original survey.

The main advantages over other approaches is: 1) it takes into account the hierarchical structure of the sample design of the original survey, and 2) it can generate trait combinations which were not present in the original survey but are likely to exist in the real population. The second is particularly important, because it avoids bias from excessive repetition of specific combination of traits present in the original survey that results from multilevel stratification of a relatively small sample. For example, the original survey may have only two 35-year-old male participants, one with a BMI of 35 and the other with a BMI of 40 and no 35-year-old male participants with BMI between 35 and 40. Unlike other methodologies, the approach proposed by Alfons et al. can produce 35-year-old male synthetic

individuals with a BMI between 35 and 40. This is possible because the synthetic population is produced by drawing from conditional distributions that were estimated from multinomial models fitted in the original survey data. The detailed statistical methodology and justification can be found elsewhere.[9]

Our approach consists of four stages from which the first is common with the original method described by Alfons et al.[9] The following stages have been adapted in order to be compatible with the widely accepted 'wider determinants of health' framework.[10] The main notion of this framework is that upstream factors such as the socioeconomic conditions, influence individual behavioural risk factors (e.g. diet, smoking), which in turn, influence individual downstream risk factors such as systolic blood pressure and total cholesterol. The four stages are:

1. Setup of the household structure.
2. Generate the socioeconomic variables.
3. Generate the behavioural variables.
4. Generate the biological variables.

In each stage, information from all previous stages is used. All the variables of the synthetic population for this study were informed by the Health Survey for England 2011 (HSE11).[11,12] The R language for statistical computing v3.2.0 and the R package 'simPopulation' v0.4.1 were used to implement the method.[4,13]

Stage 1: household structure

The household size, and the age and sex of the individuals in each household that have been recorded in HSE11 were used to inform the synthetic population, stratified by Strategic Health Authority (SHA)[*].

Stage 2: socioeconomic variables

Once the basic age, sex, household and spatial information of the synthetic population was generated, other socioeconomic information was built up. QIMD for each synthetic individual was generated dependent on the household size and the age and sex of the individuals, stratified by SHA. Then, the equivalised income quintile groups[14] (EQV5) for each household was generated, dependent on five-year age groups and sex, stratified by QIMD. Finally, the employment status of the head of the household (HPNSSEC8) was generated using the National Statistics Socio-Economic Classification[15], dependent on 5-year age groups, sex and EQV5, stratified by QIMD.

Stage 3: behavioural variables

[*] SHAs were 10 large geographic areas, part of the structure of the National Health Service in England before 2013. SHA is the only variable with spatial information in HSE11 and it was used as a proxy, to include some spatial information to the synthetic population.

In this stage, behavioural variables such as F&V portions per day, days achieving more than 30 min of moderate or vigorous PA per week,* smoking status, and exposure to ETS were generated, dependent on 5-year age groups, sex, HPNSSEC8 and EQV5, stratified by QIMD. Moreover, statins and antihypertensive medication use (two separate binary variables) were generated, dependent on 5-year age groups, sex and HPNSSEC8, stratified by QIMD. Other smoking related variables like cigarettes smoked per day for smokers, years since cessation for ex-smokers and pack-years for ever-smokers were also generated in this step.

Stage 4: biological variables

The last stage is the generation of the biological variables. Widely accepted causal pathways that have been observed in cohort studies, were used to identify associations between biological and behavioural variables. F&V consumption was used as a proxy to healthy diet. Citations refer to specific evidence regarding the associations. BMI is associated with SBP[16–19], TC[20] and DM[21]. Thus, BMI was the first to be generated in the synthetic population dependent on 5-year age groups, sex, EQV5, F&V consumption[22] and PA[22–24], stratified by QIMD. Then, DM was generated dependent on 5-year age groups, sex, HPNSSEC8 and QIMD, stratified by BMI deciles. The TC was generated dependent on 5-year age groups, sex, deciles of BMI, use of a statin and F&V consumption, stratified by QIMD. Similarly, for the SBP the 5-year age groups, sex, deciles of BMI, smoking status[25,26] and deciles of salt consumption were used as predictors, stratified by QIMD. Socioeconomic variables were used as predictors for both behavioural and biological variables to allow for possible interaction between socioeconomic and behavioural variables.

In the end, a synthetic population of 55 million synthetic individuals with similar characteristics to the non-institutionalised population of England in 2011. The synthetic population was validated against the original HSE11 sample (see p35, Synthetic population validation).

IMPACT$_{NCD}$ implementation of individualised risk factor trajectories

IMPACT$_{NCD}$ only applies the previous process for the initial year of the simulation. As the simulation evolves over time, all variables are recalculated to take into account age and period effects. This feature justifies the classification of IMPACT$_{NCD}$ as a dynamic microsimulation. The process depends on the nature of each variable and the available information but generally, it uses HSE01 – HSE12[12,27–37] to capture the time trends by age, sex, and QIMD and project them into the future.

* For PA, HSE2012 was used as HSE2011 did not measure PA.

AGE, SEX AND SOCIOECONOMIC VARIABLES

As the simulation progress in annual circles the age of the synthetic individuals in the model increase by one year in each loop. The sex and socioeconomic variables remain unchanged. Therefore, social mobility is not simulated in the current version of $\text{IMPACT}_{\text{NCD}}$.

FRUIT & VEG CONSUMPTION AND PHYSICAL ACTIVITY

Both F&V consumption (portions/day) and PA (days with more than 30 min of moderate or vigorous activity/week) were modelled as ordinal factor variables. A proportional odds logistic regression model[*] was fitted in the HSE01, HSE02, HSE04-11 individual level data with F&V consumption as the dependent variable and year, 2nd degree polynomial of age, sex, QIMD and their 1st order interactions. Similarly, for PA a similar model[†] was fitted in the HSE06, HSE08 and HSE12 data. These models were used for individual level predictions about the synthetic individuals as the simulation was evolving. Tables S3 and S4 present coefficients of the two models. Footnotes have the direct links to the actual R objects in the GitHub repository.

SMOKING

The 'close to reality' synthetic population is an accurate snapshot of active, ex-, and never smokers in 2011, as it was observed in HSE11. Then $\text{IMPACT}_{\text{NCD}}$ uses transitional probabilities for smoking initiation[‡], cessation[§], and relapse[**], to generate and record smoking histories of the synthetic individuals. For smoking initiation and cessation probabilities, logistic regression models were fitted in HSE data with age, sex, and QIMD as the independent variables. A similar approach was followed for relapse probabilities with years since cessation, sex and QIMD as the independent variables. Tables S5, S6, and S7 present coefficients of the models. Footnotes have the direct links to the actual R objects in the GitHub repository.

ENVIRONMENTAL TOBACCO SMOKING

For ETS we assumed a linear relation between smoking prevalence and ETS, stratified by QIMD. The models are estimated dynamically during the simulation. We assumed no intercept; when smoking prevalence reaches 0, ETS prevalence will be 0 too.

CONTINUOUS BIOLOGICAL VARIABLES

In $\text{IMPACT}_{\text{NCD}}$ the value of each continuous biological risk factor (BMI, SBP, and TC) is calculated in a two-stage process for each synthetic individual and each projected year. The first stage simulates

[*] https://github.com/ChristK/IMPACTncd/blob/CVD-policy-options/Lagtimes/fv.svylr.rda
[†] https://github.com/ChristK/IMPACTncd/blob/CVD-policy-options/Lagtimes/pa.svylr.rda
[‡] https://github.com/ChristK/IMPACTncd/blob/CVD-policy-options/Lagtimes/smok.start.svylr.rda
[§] https://github.com/ChristK/IMPACTncd/blob/CVD-policy-options/Lagtimes/smok.cess.svylr.rda
[**] https://github.com/ChristK/IMPACTncd/blob/CVD-policy-options/Lagtimes/smok.cess.success.rda

ageing effects, while the second stage simulates period effects. We follow this approach mainly for two reasons. Firstly, to simulate physiological mechanisms of ageing. For example, the change of lipid profile in post-menopausal women, or the increase of SBP due to age-related stiffening of the arteries. Secondly, because the variance of the risk factor distributions increases with age, and we wanted to model this phenomenon. Below we describe the stages:

Stage 1: Instead of tracking the actual biological risk factor values for the synthetic individuals, we track the percentile ranks[*] of the values by age, sex and QIMD. These percentile ranks remain fixed for each synthetic individuals throughout his/her life course. In each simulated year, the percentile ranks are converted back to actual risk factor values, by matching to the percentile ranks of a sample of the initial synthetic population of same age group, sex, and QIMD.

For example, in 2011 a 20-year-old male synthetic individual living in a QIMD 3 area with SBP of 120 mmHg has a SBP percentile rank of 0.52. Fifty years later, the same synthetic individual has preserve his percentile rank score for SBP. However, his SBP is now calculated to 137.6 mmHg in order to match the SBP of a 70-year old man living in a QIMD 3 area in 2006 with the same percentile rank of 0.52. Figure S2 illustrates the previous example. Despite, individuals preserve their percentile for the respective risk factor throughout the simulation (vertical position in Figure S2), this stage remains stochastic, because each time this stage is implemented a different sample from the synthetic population is drawn. Finally, the distance from the mean for each risk factor is calculated stratified by 5-year age group, sex, and QIMD. For instance, if a synthetic individual has SBP of 140 mmHg and the mean SBP in the respective group of same age group, sex and QIMD is 130 mmHg, the distance from the mean is 140 – 130 = 10 mmHg.

Stage 2: Similarly to the approach followed for other variables, we fitted regression models to the HSE01-12 data. For BMI[†], year, age, sex, QIMD and PA were the independent variables. For SBP[‡], year, age, sex, QIMD, smoking status, BMI, and PA were the independent variables. Finally, for TC[§], year,

---

[*] For the percentile rank the formula $R_{percentile} = (R-1)/(n-1)$ is used, where $R_{percentile}$ is the percentile rank and $R = (R_1, \dots, R_n)$ is the rank vector constructed from a random observation vector $(X_1, \dots, X_n)$. In IMPACTNCD specifically, vector X is constructed from the subset of the respective continuous risk factor values, by 5-year age group, sex and QIMD, for each year of the simulation.

[†] https://github.com/ChristK/IMPACTncd/blob/CVD-policy-options/Lagtimes/bmi.svylm.rda
[‡] https://github.com/ChristK/IMPACTncd/blob/CVD-policy-options/Lagtimes/sbp.svylm.rda
[§] https://github.com/ChristK/IMPACTncd/blob/CVD-policy-options/Lagtimes/chol.svylm.rda

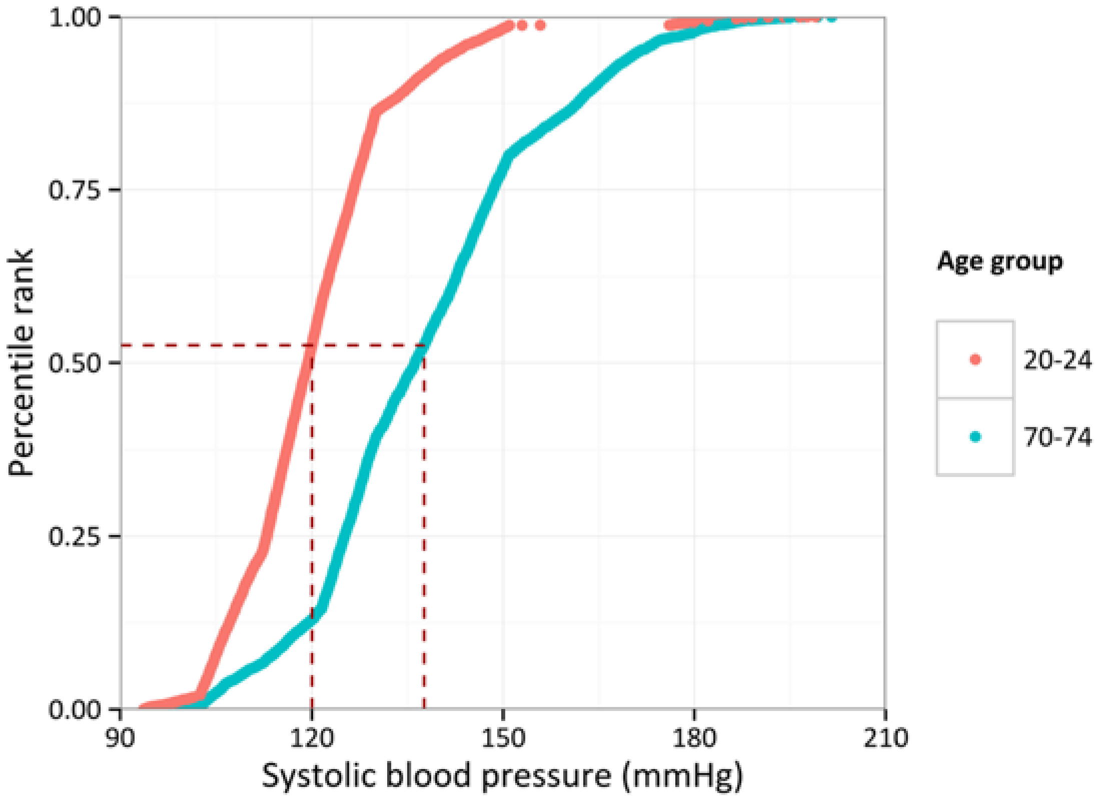

Figure S2 Plot of the percentile rank against the systolic blood pressure of male synthetic individuals living in QIMD 3 area for age groups 20-24 and 70-74.

age, sex, QIMD, BMI, F&V consumption and PA were the independent variables.* These models are used to predict the mean of the relevant group. These predicted means are added then, to the distances calculated in previous stage. The result is the final value of the relevant risk factor that will be used for risk estimation. Tables S8, S9, and S10 present coefficients of the models. Footnotes have the direct links to the actual R objects in the GitHub repository.

Diabetes mellitus

As with smoking, the 'close to reality' synthetic population is an accurate snapshot of diagnosed and non-diagnosed diabetics in 2011, as it was observed in HSE11. We assumed DM is an incurable chronic condition. $IMPACT_{NCD}$ uses the validated for English population Qdiabetes algorithm (ex QDscore) to calculate annual transitional probabilities of non-diabetic synthetic individuals to develop DM.[38]

* As before, the independent variables for each risk factor were selected based on known associations from longitudinal studies. Therefore, only the magnitude of the association is informed by cross-sectional data and possibly attenuated due to reverse causality.

Lag times

All the function that have been described above for risk factor trajectories include time and age (in years) as one of the independent variables. Therefore, lag times can be potentially considered on a per risk factor basis. For instance, let us consider a 50-year-old synthetic individual in 2015 and an assumed lag time of 5 years for F&V. When $\text{IMPACT}_{\text{NCD}}$ calculates the probabilities for F&V consumption of this individual, it will use time – (lag time) = 2015 – 5 = 2010 and age – (lag time) = 50 – 5 = 45. So, when the 'disease' module of $\text{IMPACT}_{\text{NCD}}$, uses the risk exposure to F&V to estimate a disease incidence transitional probability, the lag timed exposure will be used.

For the sake of simplicity, in this study we assumed that the lag time between exposure and CVD is 5 years.[39–41]. The lag time was roughly informed from risk reversibility trials, when available, or the median observation times of the cohort studies we used to inform the risk magnitude for each risk factor.

Birth engine (Step 4)

The Office for National Statistics (ONS) principal-assumption fertility projections for England are used to estimate the number of new synthetic individuals entering the model through birth, in every simulated year.[42] The birth engine only becomes important for simulations featuring a horizon of more than 30 years. Therefore, we do not describe this step in detail here.

CHAPTER 2. DISEASE MODULE

The disease module contains the last three steps of the model (Figure S1). The risk (probability) for each synthetic individual aged 30 – 84, to develop each of the modelled diseases is estimated in step 5 conditional on the exposure to relevant risk factors. The step ends by selecting synthetic individuals to develop the modelled diseases. Finally, in steps 6 and 7 the risk of dying from one of the modelled diseases or any other cause is estimated and applied. Steps 2 to 7 are then repeated for the surviving individuals until the simulation horizon is reached.

Estimating the annual individualised disease risk and incidence (Step 5)

In order to estimate the individualised annual probability of a synthetic individual to develop a specific disease conditional on his/her relevant risk exposures we follow a 3-stage approach:

1. The proportion of incidence attributable to each modelled risk factor by age group and sex is estimated, assuming a specific time lag.
2. Assuming multiplicative risks, the portion of the disease incidence attributable to all the modelled risk factors is estimated and subtracted from the total incidence.
3. For each individual in the synthetic population, the probability to develop the disease is estimated and then is used in an independent Bernoulli trial to select those who finally develop the disease.

Next, the implementation of the above method is described in more detail using CHD as an example. The same process is used for stroke.

Stage 1

The population attributable risk (PAF) is an epidemiological measure that estimates the proportion of the disease attributable to an associated risk factor.[43] It depends on the relative risk associated with the risk factor and the prevalence of the risk factor in the population. Specifically, for each modelled binary risk factors associated with CHD, PAF was calculated by 5-year age group and sex using the formula:

$$PAF = \frac{P * (RR - 1)}{P * (RR - 1) + 1} ,$$

where P is the prevalence of the risk factor in the population, and RR is the relative risk of the risk factor. For categorical risk factors with n levels of exposure, we used the formula:

$$PAF = \frac{\sum_{i=1}^{n} P_i * (RR_i - 1)}{\sum_{i=1}^{n} P_i * (RR_i - 1) + 1} ,$$

where P_i is the prevalence of the risk factor at level i in the population and RR_i is the relative risk associated with i level of exposure. The same formula was used to approximate the PAF of continuous risk factors because they behave like discrete variables in the model. Consistent with findings from the respective meta-analyses that were used for $\text{IMPACT}_{\text{NCD}}$ (Error! Reference source not found.), SBP below 115 mmHg, TC below 3.8 mmol/l and BMI below 20 Kg/m^2 were considered to have a relative risk of 1. Similarly, consumption of 8 or more portions of F&V and 5 or more days with more than 30 minutes of moderate to vigorous activity per week were also considered to have a relative risk of 1.

For the estimation of prevalence of risk factors, we used their prevalence in the synthetic population taking into account any assumed lag time. All the relative risks were taken from published meta-analyses and cohort studies (**Error! Reference source not found.**).

Stage 2

Assuming multiplicative risk factors with no interactions, the incidence of CHD not attributable to the modelled risk factors can be estimated by the formula:

$$I_{Theoretical\ minimum} = I_{Observed} * (1 - PAF_1) * (1 - PAF_2) * (1 - PAF_3) * \ldots * (1 - PAF_m)$$

Where $I_{Observed}$ is the CHD incidence and $PAF_{1\ldots m}$ are the PAF of each risk factor from Step 1. $I_{Theoretical\ minimum}$ represents CHD incidence if all the modelled risk factors were at optimal levels. The theoretical minimum incidence is calculated by 5-year age group and sex only in the initial year of the simulation and it is assumed stable thereafter.

Stage 3

Assuming that $I_{Theoretical\ minimum}$ is the baseline annual probability of a synthetic individual to develop CHD for a given age and sex due to risk factors not included in the model (i.e. genetics etc.), the individualised annual probability to develop CHD, $\mathbb{P}(\text{CHD} \mid \text{age, sex, exposures})$, given his/her risk factors were estimated by the formula:

$$\mathbb{P}(CHD \mid age, sex, exposures) = I_{Theoretical\ minimum} * RR_1 * RR_2 * RR_3 * \ldots * RR_m$$

Where $RR_{1\ldots m}$ the relative risks that are related to the specific risk exposures of the synthetic individual, same as in stage 1. Depending on data availability this method can be further stratified by QIMD; however, data were not available for this in the current study.

The above method can be used only when the incidence of the disease in the population is known. The true incidence of CHD (and stroke) though, is largely unknown. Several estimates exist nonetheless all have limitations. Therefore, for the estimation of CHD incidence by age and sex we

opted for a modelling solution to synthesise all the available sources of information and minimise bias. Specifically, we used ONS CHD mortality (ICD10 I20-I25) for England in 2011,[44] self-reported prevalence of CHD from HSE11, incidence of angina from primary care data[45] and incidence of acute myocardial infarction (AMI) from mortality and hospital statistics[46] to inform the World Health Organisation (WHO) DISMOD II model.[47] DISMOD II is a multi-state life table model that is able to estimate the incidence, prevalence, mortality, fatality and remission of a disease, when information about at least three of these indicators is available. A similar approach has been followed by the Global Burden of Disease team and others.[48,49] We considered CHD an incurable chronic disease (i.e. remission rate was set to 0); therefore, the derived DISMOD II incidence refers to the first ever manifestation of angina or AMI excluding any recurrent episodes. For the DISMOD II calculations, we assumed that incidence and case-fatality had been declining by 3% (relative), over the last 20 years. The derived CHD incidence, prevalence, and fatality were used as an input for $\text{IMPACT}_{\textbf{NCD}}$. Similar approach was used for stroke.

For the initial year of the simulation, some synthetic individuals need to be allocated as prevalent cases for each of the modelled diseases. DISMOD II model[47] is used again to estimate the number of prevalent cases of the disease by age and sex. Then, the estimated number of prevalent cases are sampled independently from the individuals in the population with weights proportional to their relevant exposures to the associated risk factors.

Simulating disease histories (Step 6)

In the current stage of development, $\text{IMPACT}_{\textbf{NCD}}$ does not contain a detailed disease history module. However, Step 6 is used to simulate significant aspects of the disease. For CVD, this was used to simulate the observable spike of short-term (30 days) mortality after the first event of AMI or stroke. Data about short term mortality were used from the 'Coronary heart disease statistics 2012 edition' report.[45]

Simulating mortality (Step 7)

All synthetic individuals are exposed to the risk of dying from any of their acquired modelled diseases or any other non-modelled cause. However, the algorithm behaves differently depending on the age and life course trajectory of the synthetic individual.

For ages 0 to 29, we used all-cause mortality rate by age, sex, and QIMD to inform an independent Bernoulli trial and select synthetic individuals that die every year. For years 2011 to 2013 we used the observed mortality rates as were reported from ONS.[44] For years after 2013, functional demographic models by sex and QIMD were fitted to the ONS reported annual mortality rates, from years 2002 to 2013, and then they were projected to the simulation horizon using the R package 'demography'.[50]

Functional demographic models are generalisations of the Lee-Carter demographic model, influenced by ideas from functional data analysis and non-parametric smoothing.[51]

The same approach as above was followed for synthetic individuals aged 85 to 100. We considered a mortality rate of 1 for all synthetic individuals reaching the age of 100. Hence, IMPACT$_{\textbf{NCD}}$ maximum synthetic individual age is 100 years.

Finally, for synthetic individuals with ages between 30 and 84 the all-cause mortality was decomposed into modelled-diseases specific mortality and any-other cause mortality. The former applies only to the prevalent cases of each modelled disease in the synthetic population. For this, case-fatality rates by age and sex were estimated by DISMOD II for each modelled disease, as described before. Then case-fatality rates are used in a Bernoulli trial to select prevalent cases that die from the disease in a year.

For the any-other cause mortality, a process similar to the one described for ages 0 to 29 and 85 to 100. However, this time CHD and stroke specific mortality are removed from the observed mortality and mortality projections to avoid double counting.

The case mortality and fatality rates are further parametrised and individualised based on established epidemiological evidence. The 'male British doctors' and DECODE studies have showed that smokers and diabetics have increased overall mortality even when CVD was excluded[52,53]. IMPACT$_{\textbf{NCD}}$ adjusts for that by inflating the any-other cause mortality rate for smokers and diabetics and deflating it for non-smokers and non-diabetics, while it constrains the sum to remain the same as before the adjustments. Furthermore, we assumed that CVD case-fatality is improving by 3%, and that there is a constant case-fatality socioeconomic gradient of approximately 5% by QIMD level (halved for ages over 70) for CHD, and 2% for stroke. The socioeconomic gradient forces the more deprived to experience worse disease outcomes. These assumptions are based on empirical evidence.[45]

Finally, synthetic individuals who remain alive after this step progress to the next year and start again from step 1, unless the simulation horizon has been reached.

CHAPTER 3. SCENARIOS

The method described above, is used to for the baseline scenario. In general, primary prevention interventions or policies can then be modelled as counterfactual scenarios, through their estimated effects on the relevant risk factors. The modelled scenarios are generated from alterations of the baseline scenario, mainly in three ways:

1. Population-wide interventions can be modelled, by altering the intercept or the coefficients of the regression equations that are used to estimate risk factor exposures. For example, when continuous risk factors are considered, adding or subtracting from the intercept increases or decreases the related risk factor for each synthetic individual; therefore, the mean of the risk factor for the whole population. Altering the year coefficient accelerates, decelerates or reverses the trend for the whole population. Likewise, altering the QIMD coefficients or/and the coefficient of the interaction between year and QIMD can simulate differential effects and trends by QIMD. A similar approach sometimes can be used also for the non-continuous risk factors. The benefit is that by just altering a few parameters the changes are translated down to individual level characteristics in a computationally efficient way.
2. Targeted interventions can be modelled by selecting synthetic individuals with a specific trait or combination of traits, and apply an intervention to them, by changing their attributes. For example, to simulate the effect of statins a simple approach would be to randomly select 30% of the synthetic individuals with TC higher than 4 mmol/l not currently on statins; and apply a 25% reduction of their TC between steps 4 and 5 (Figure S1).
3. Some hybrid combination of the previous methods or some 'exotic' approaches like have the time stop at a specific year, or running backwards to simulate disaster scenarios etc.

In the following paragraphs, we highlight some details of the scenarios that we used in the main paper. They are meant to be read in conjunction with the scenario description in the main text.

Universal screening

This was a typical targeted intervention, so this scenario was built with the second approach, as described above. The high-risk synthetic participants eligible for treatment were selected based on the QRISK2 score.[54] The score requires extra information about the synthetic individual that was not originally modelled and at the current stage is used exclusively for the calculation of the QRISK2 score. This includes information about ethnicity, specific type of diabetes (I or II), family history of CVD, chronic kidney disease (stage 4 or 5), atrial fibrillation, rheumatoid arthritis, and the TC/HDL ratio. To model these extra attributes for the synthetic individuals we fitted multinomial, logistic or generalised linear regression models to HSE data, and then we used the models to predict synthetic individuals'

status. Exceptions, to this approach were type I diabetes and rheumatoid arthritis prevalence. We assumed a prevalence of 0.5% for type I diabetes and we extracted age and sex specific rheumatoid arthritis prevalence from published data.[55]

To simulate ethnicity of synthetic individuals, a multinomial model was fitted to HSE data with 5-year age group, sex and QIMD as the independent variables.[*] To simulate family history of CVD, a logistic regression model was fitted in HSE06 data that contained this information, with age and QIMD as the independent variables.[†] For the prevalence of atrial fibrillation, a logistic regression model was fitted in HSE11 data that contained this information, with age and QIMD and smoking status as the independent variables.[‡] To model the prevalence of chronic kidney disease a logistic regression model with age, sex, and QIMD as independent variables was fitted to HSE10 data.[§] Finally, for TC/HDL ratio a GLM was fitted to HSE data, with TC, age, sex, QIMD, BMI, PA, and smoking status as the independent variables.[**]

To model the effect of Atorvastatin 20mg we used evidence from Law at al. and Edwards et al. and we estimated ~32% reduction in TC.[56,57] To estimate the individualised effectiveness of Atorvastatin, we used the formula:

$$Individualised\ effectiveness = Effectiveness * Prescription * Persistence * Adherence$$

Where *Prescription* is a binary variable (whether Atorvastatin was prescribed (1), or not (0)), *Persistence* is a binary variable (whether the synthetic individual continue with the medication (1), or not (0)), and *Adherence* is a value between 0 and 1 modelling the proportion of daily dose taken. For these variables, values where drawn from distributions (Table S14).

A similar approach was used for antihypertensive medication. Given the numerous antihypertensive treatment combinations, we assumed that medication could fully control hypertension for all synthetic individuals down to a target of 115mmHg of SBP. We applied the same approach as above to adjust treatment effectiveness to prescription, persistence, and adherence.

Information regarding medication prescription after a Health Check was extracted from Forster et al.[58] This study was conducted while the recommendation for primary prevention statin prescription was

---

[*] https://github.com/ChristK/IMPACTncd/blob/CVD-policy-options/Lagtimes/origin.multinom.rda
[†] https://github.com/ChristK/IMPACTncd/blob/CVD-policy-options/Lagtimes/famcvd.svylr.rda
[‡] https://github.com/ChristK/IMPACTncd/blob/CVD-policy-options/Lagtimes/af.svylr.rda
[§] https://github.com/ChristK/IMPACTncd/blob/CVD-policy-options/Lagtimes/kiddiag.svylr.rda
[**] https://github.com/ChristK/IMPACTncd/blob/CVD-policy-options/Lagtimes/tctohdl.svylm.rda

based on 20% risk for a CVD event in 10 years. Yet, statin prescription was low in this group and statins were prescribed to individuals with lower than 20% risk. We chose to inflate the reported from Forster et al. prescription rate for participants with a risk between 10% and 20%. This was made to reflect the recent change in recommendation about statin prescription for primary prevention, which lower the threshold from 20% to 10% risk for a CVD event in 10 years. We avoided making it equal to the prescription rate of those with a risk higher than 20% based on finding from Usher-Smith et al. that reported reluctant statin uptake to the newly eligible population.[59]

In one of the simulated scenarios for sensitivity analysis, we assumed a treatment threshold of 20% risk for a CVD event in 10 years. For this scenario, we used prescription rates as reported from Forster et al. for the participants with a risk higher than 20%.[58] Yet, we also allowed synthetic participants to be prescribed medication with a risk higher than 10% as was reported by Forster et al. The justification was that despite the recommended 20% threshold to offer treatment to high-risk individuals, when the study from Forster et al. was contacted, participants with lower risk were still prescribed medication.

Population-wide intervention

Many of the interventions in this scenario were modelled by altering the coefficients of the models that were used to estimate the attributes of the synthetic individuals. Specifically, this approach was followed for BMI and SBP. Smoking and F&V consumption interventions were modelled by altering the attributes of synthetic individuals after they were estimated in step 2 (Figure S1). Given the existing limitations to measure the direct effect of a structural population-wide intervention, we inflated the uncertainty around the inputs we have used for this scenario (Table S14).

Risk factors trajectories

The effects of all modelled scenarios on population risk factors are summarised in the following graphs, for ages 30 to 84.

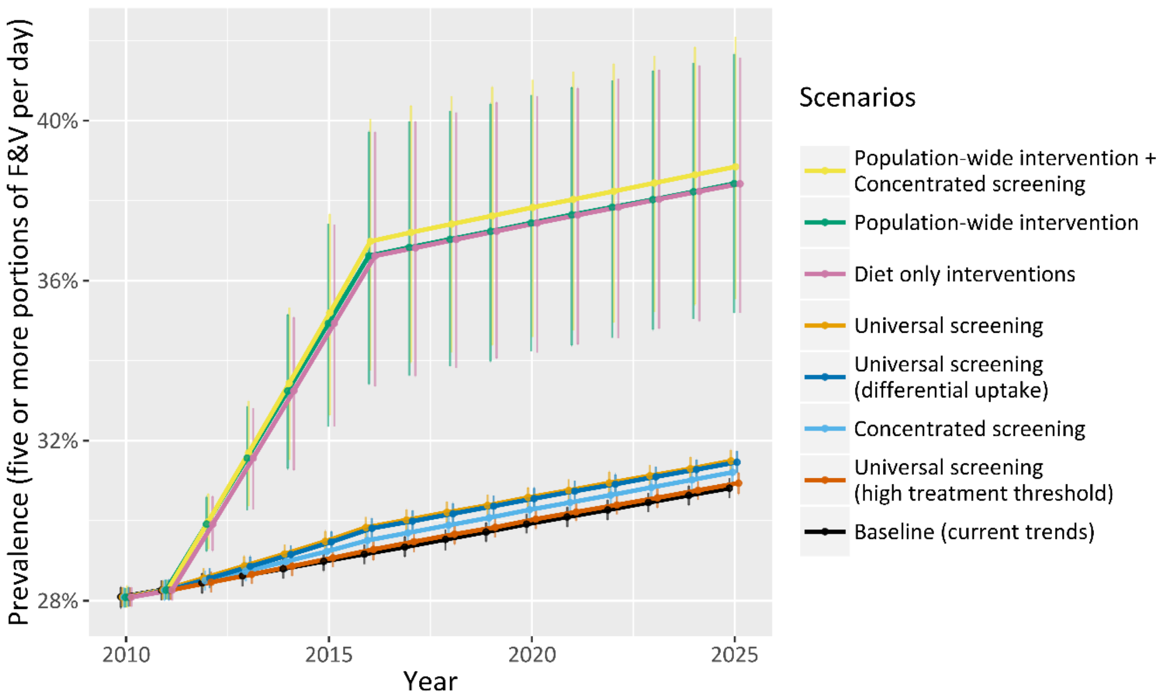

Figure S3 Proportion of the synthetic population achieving the target of five or more portions of fruit and vegetable per day for each scenarios. Error bars represent 95% uncertainty intervals.

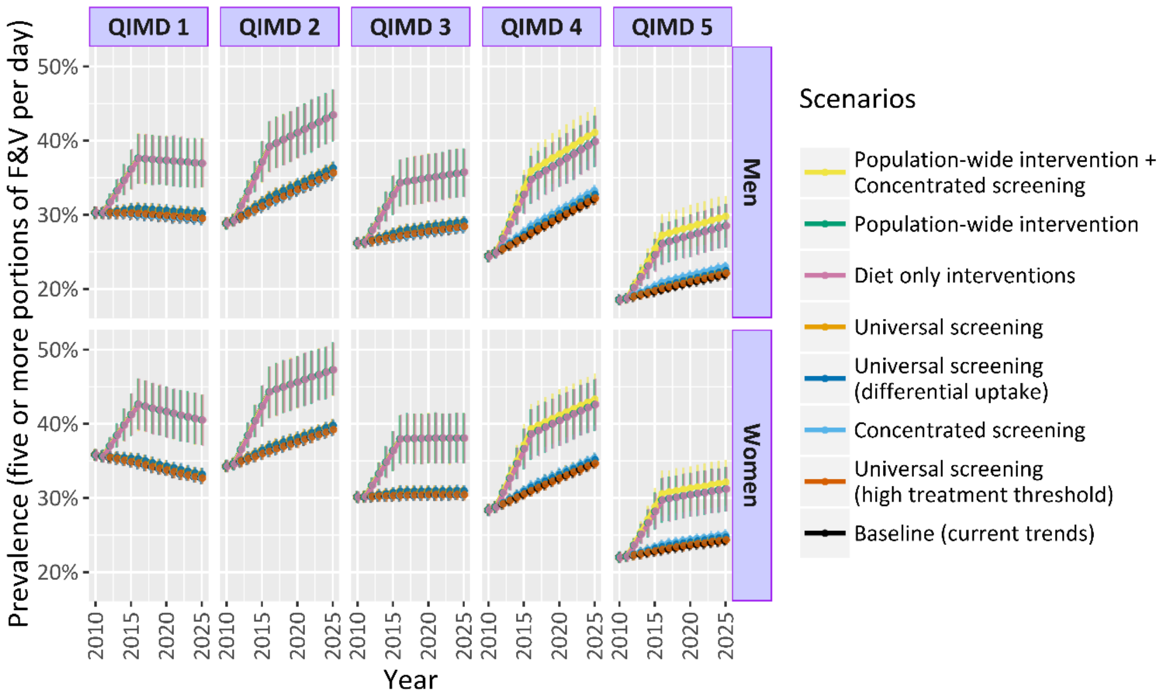

Figure S4 Proportion of the synthetic population achieving the target of five or more portions of fruit and vegetable per day for each scenarios, by sex and quintile group of multiple deprivation (QIMD, 1=most affluent, 5= most deprived). Error bars represent 95% uncertainty intervals.

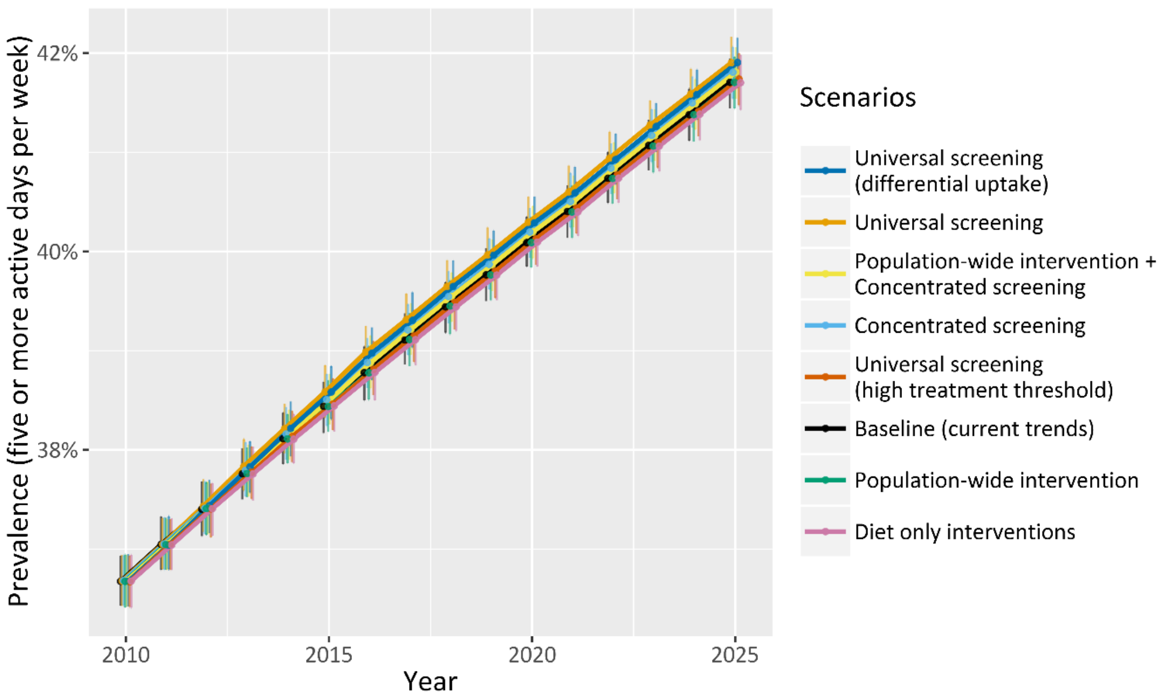

Figure S5 Proportion of the synthetic population achieving the target of five or more active days per week for each scenarios. Error bars represent 95% uncertainty intervals.

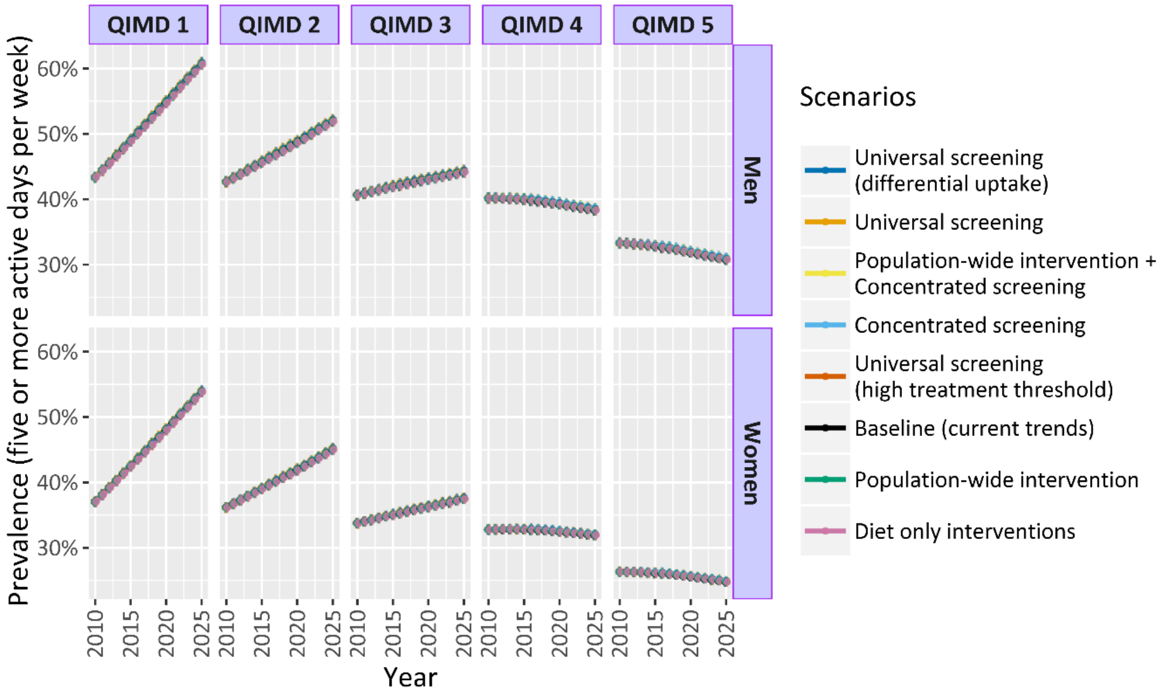

Figure S6 Proportion of the synthetic population achieving the target of five or more active days per week for each scenarios, by sex and quintile group of multiple deprivation (QIMD, 1=most affluent, 5= most deprived). Error bars represent 95% uncertainty intervals.

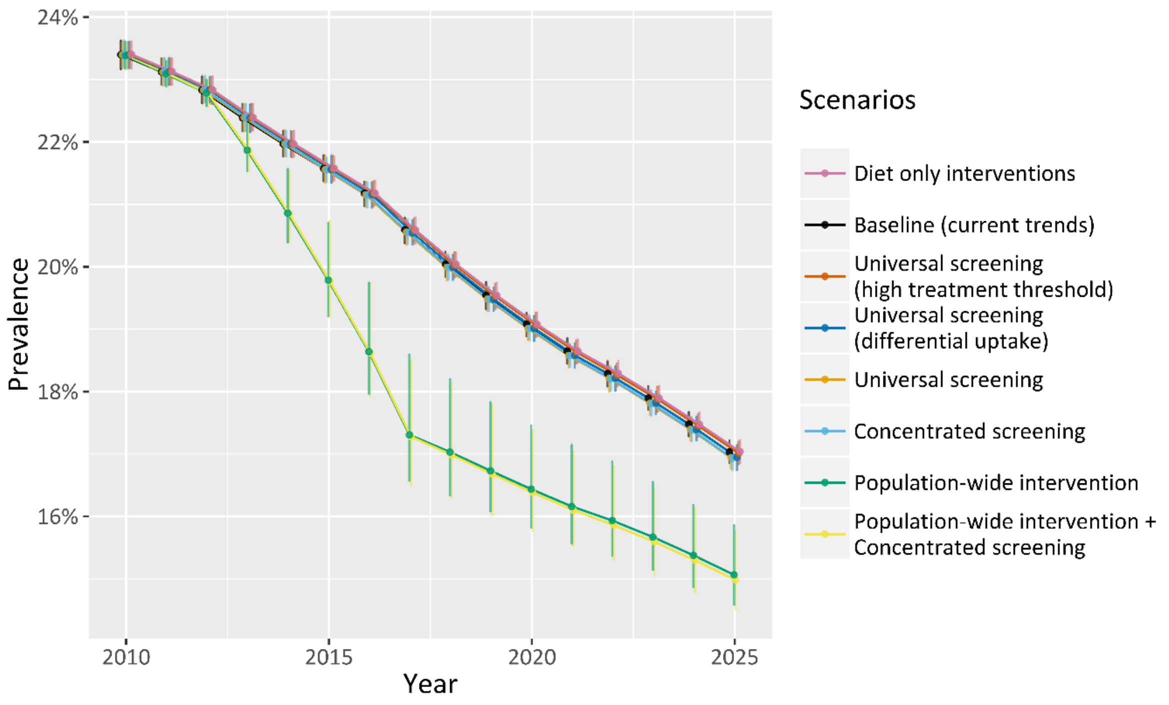

Figure S7 Smoking prevalence in the synthetic population for each scenario. Error bars represent 95% uncertainty intervals.

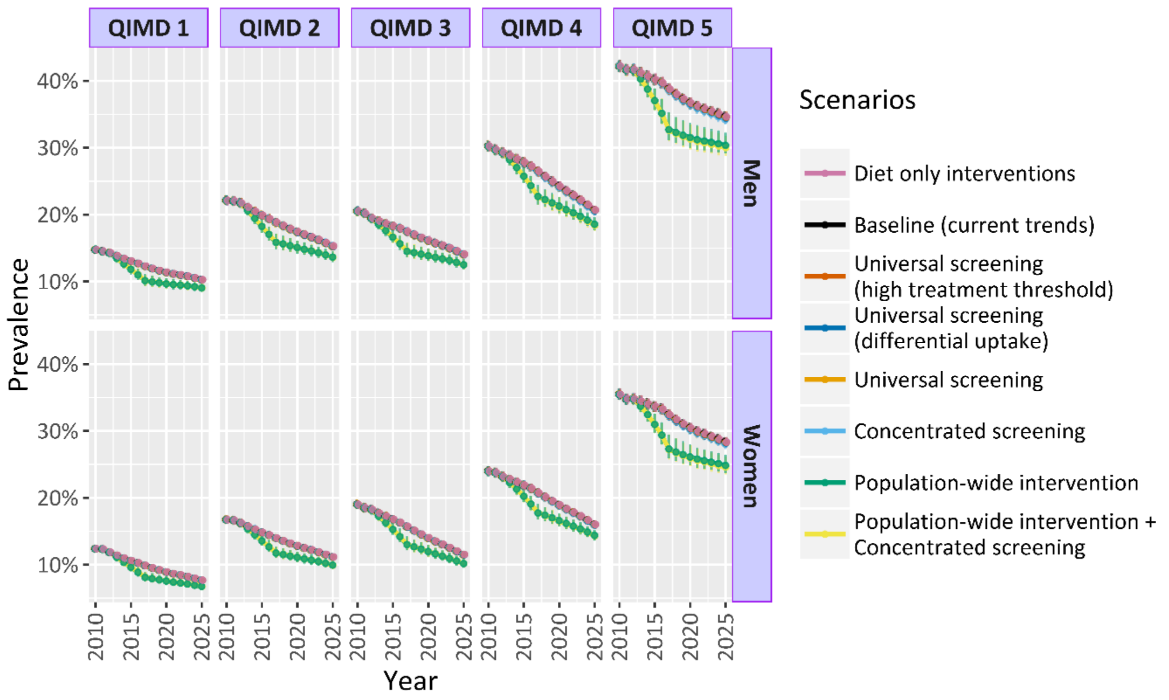

Figure S8 Smoking prevalence in the synthetic population for each scenario, by sex and quintile group of multiple deprivation (QIMD, 1=most affluent, 5= most deprived). Error bars represent 95% uncertainty intervals.

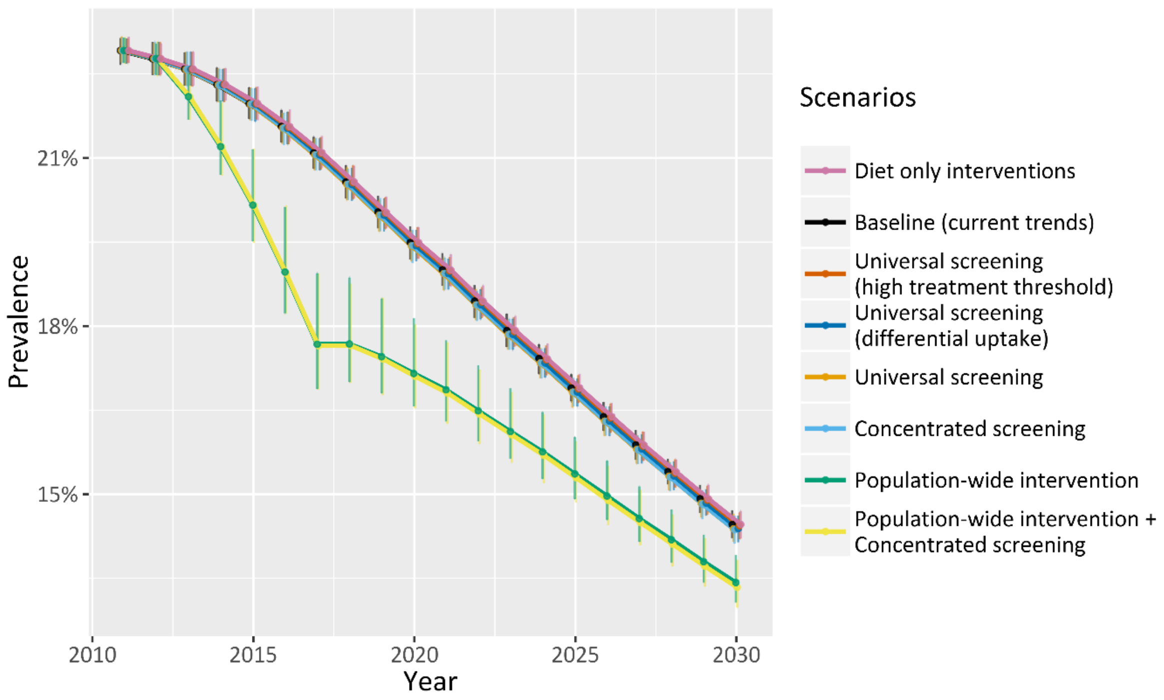

Figure S9 Environmental tobacco smoking prevalence in the synthetic population for each scenario. Error bars represent 95% uncertainty intervals.

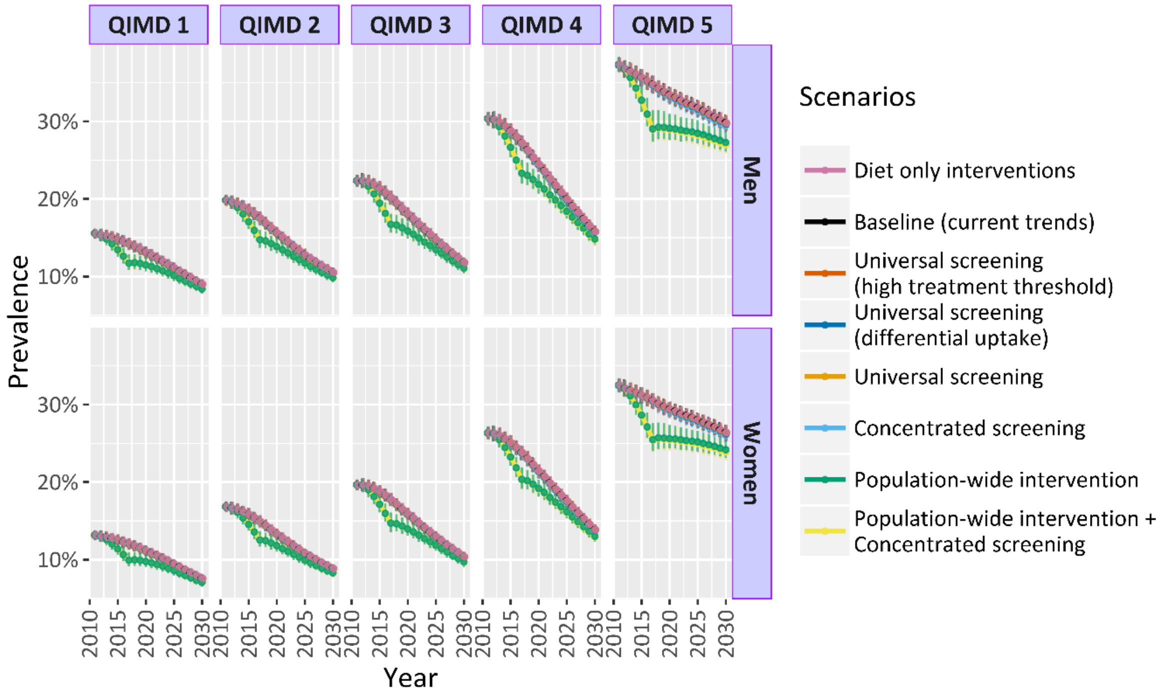

Figure S10 Environmental tobacco smoking prevalence in the synthetic population for each scenario, by sex and quintile group of multiple deprivation (QIMD, 1=most affluent, 5= most deprived). Error bars represent 95% uncertainty intervals.

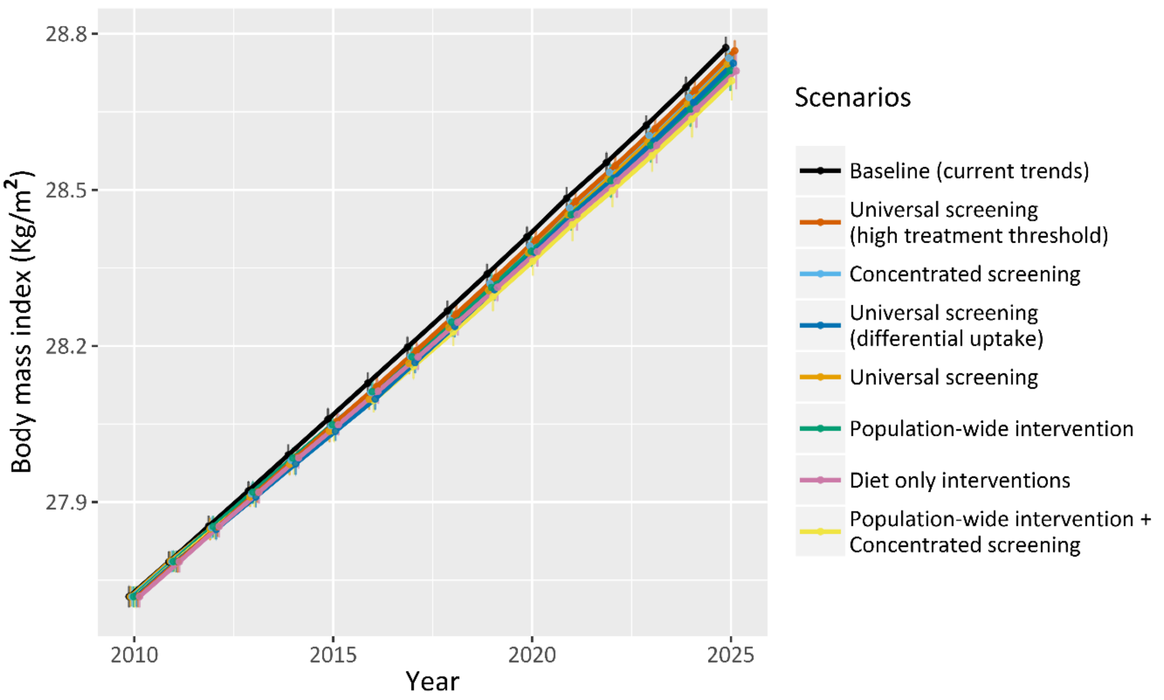

Figure S11 Mean body mass index in the synthetic population for each scenario. Error bars represent 95% uncertainty intervals.

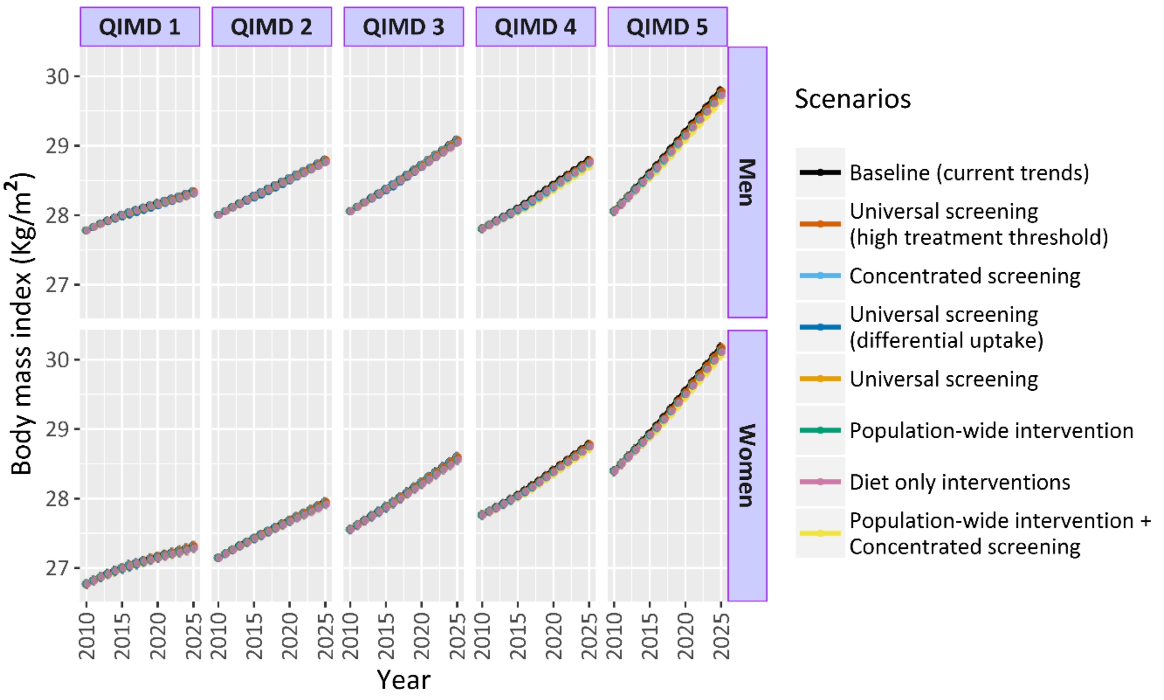

Figure S12 Mean body mass index in the synthetic population for each scenario, by sex and quintile group of multiple deprivation (QIMD, 1=most affluent, 5= most deprived). Error bars represent 95% uncertainty intervals.

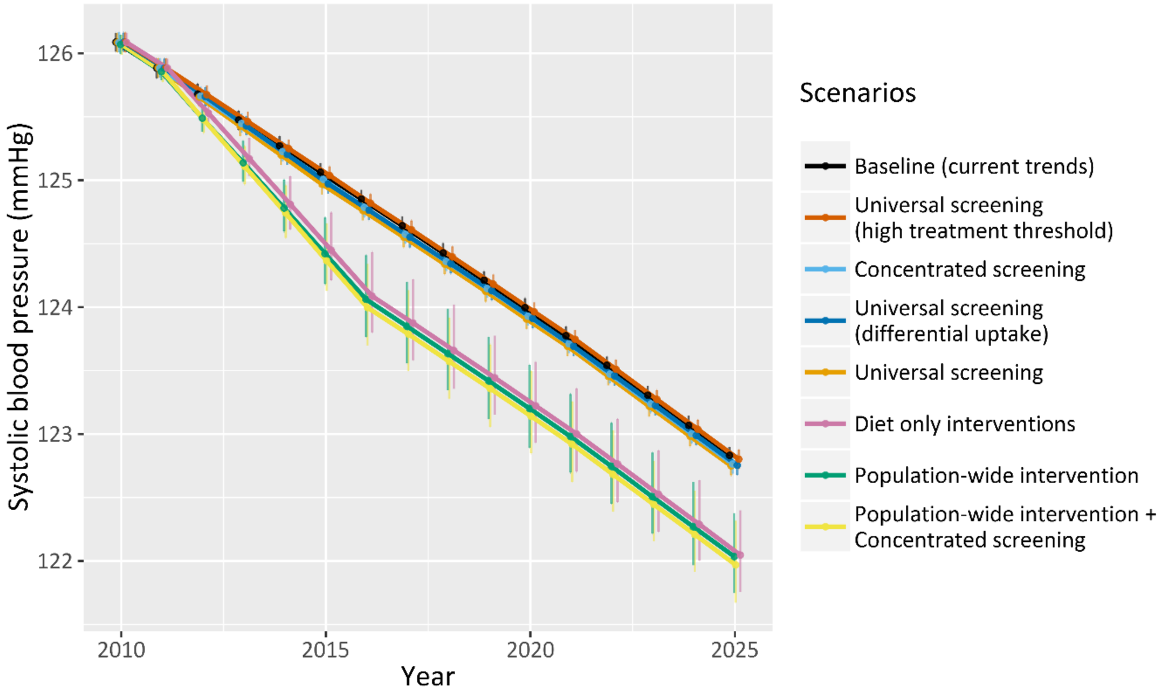

Figure S13 Mean systolic blood pressure in the synthetic population for each scenario. Error bars represent 95% uncertainty intervals.

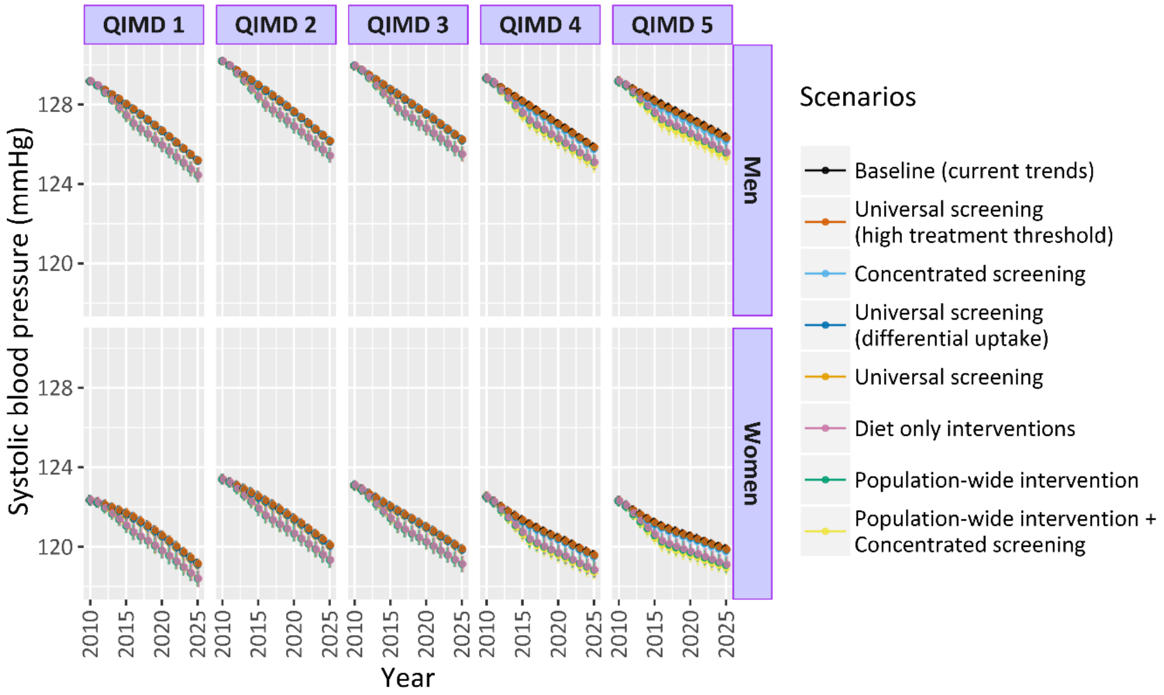

Figure S14 Mean systolic blood pressure in the synthetic population for each scenario, by sex and quintile group of multiple deprivation (QIMD, 1=most affluent, 5= most deprived). Error bars represent 95% uncertainty intervals.

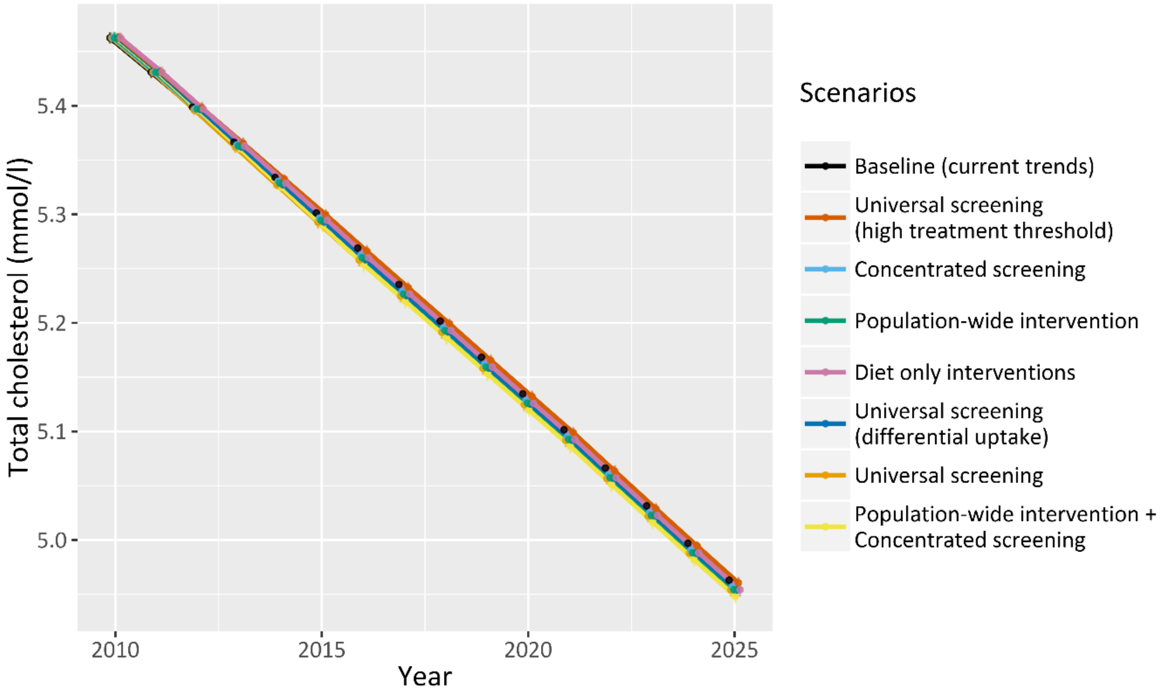

Figure S15 Mean total cholesterol in the synthetic population for each scenario. Error bars represent 95% uncertainty intervals.

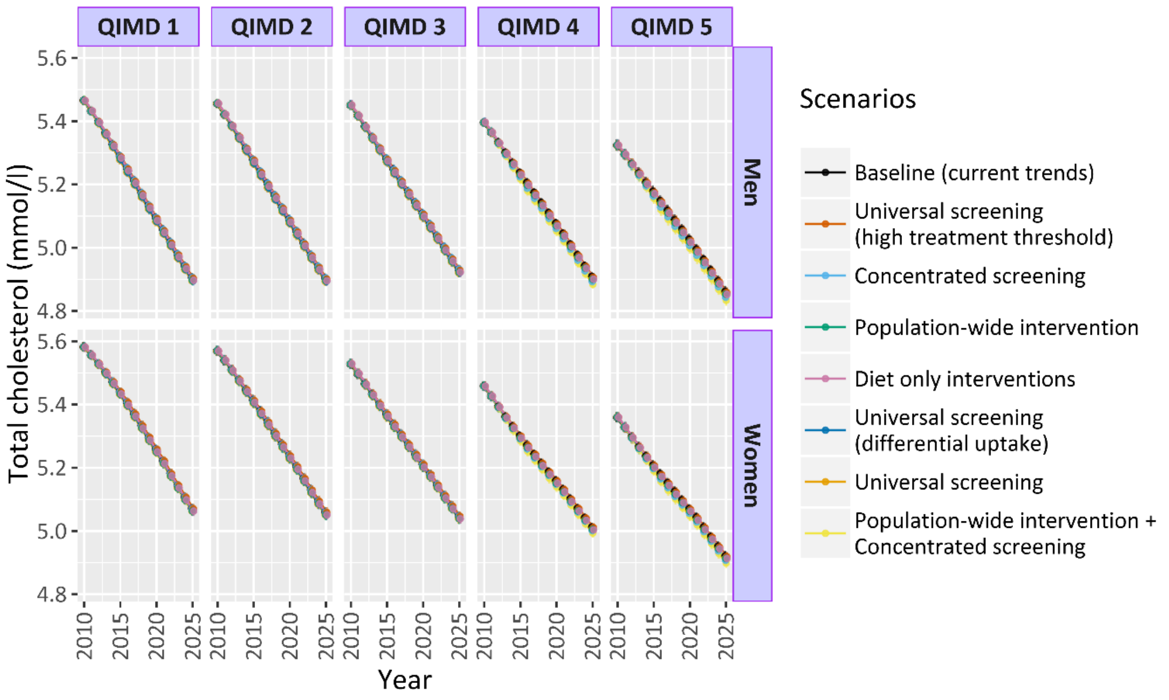

Figure S16 Mean total cholesterol in the synthetic population for each scenario, by sex and quintile group of multiple deprivation (QIMD, 1=most affluent, 5= most deprived). Error bars represent 95% uncertainty intervals.

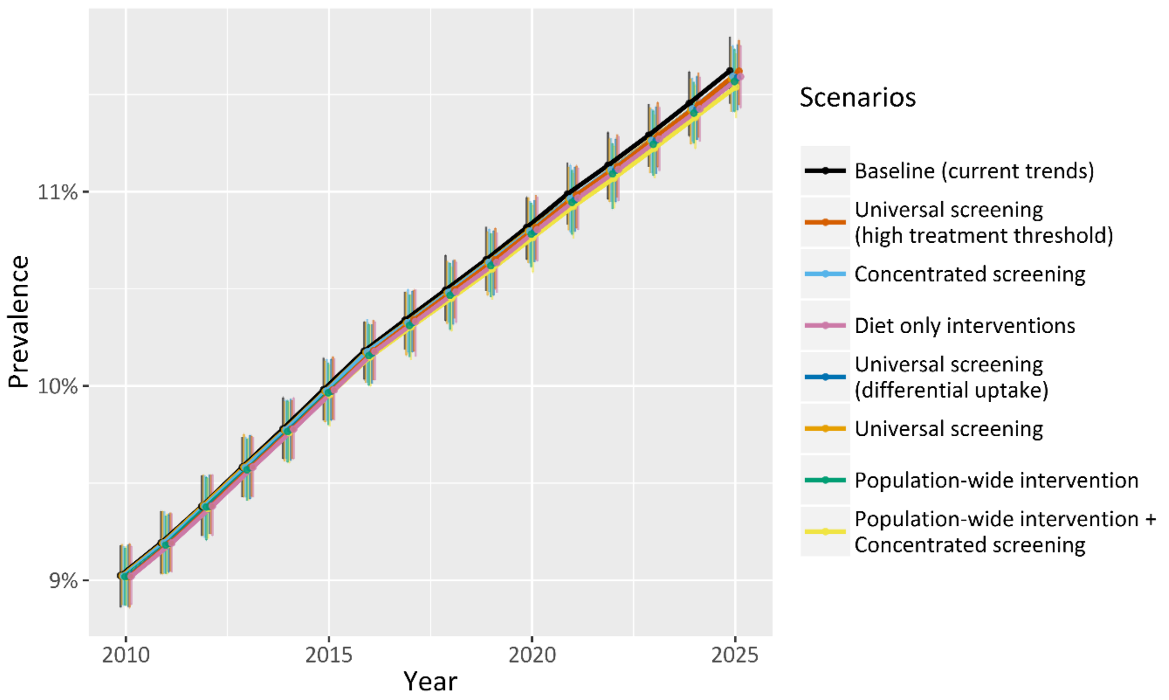

Figure S17 Diabetes prevalence in the synthetic population for each scenario. Error bars represent 95% uncertainty intervals.

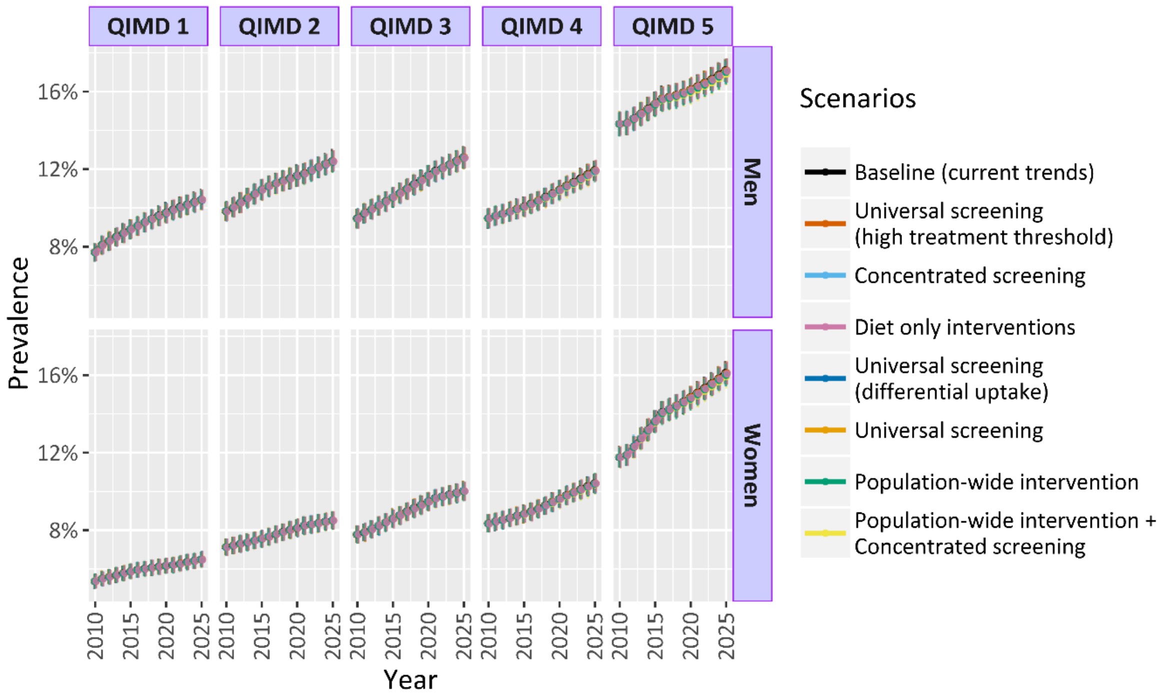

Figure S18 Diabetes prevalence in the synthetic population for each scenario, by sex and quintile group of multiple deprivation (QIMD, 1=most affluent, 5= most deprived). Error bars represent 95% uncertainty intervals.

CHAPTER 4. SENSITIVITY ANALYSIS

Here we present the full output of the three scenarios that were produced as variations of the main scenarios with modified assumptions; namely the ‘20% treatment threshold universal screening’, the ‘socioeconomic differential uptake universal screening’, and the ‘diet-only population-wide intervention’. Tables S11, S12, and S13 summarise the results.

CHAPTER 5. UNCERTAINTY

$IMPACT_{NCD}$ implements a 2nd order Monte Carlo approach to estimate uncertainty distributions of the outputs for each scenario.[60,61] Each simulation runs 1000 times. For each iteration, a different set of input parameters is used, by sampling from the respective distributions* of input parameters, and a different sample of the synthetic population is drawn. However, the scenarios are 'paired'. For instance, the *n*th iteration of all scenarios runs with the same set of input parameters and on the same initial synthetic population sample for all of them.† This explains why the uncertainty of in-between scenarios comparisons is significantly smaller than the uncertainty of isolated scenarios.

The framework allows stochastic uncertainty, parameter uncertainty and individual heterogeneity to be reflected in the reported uncertainty intervals (UI). The following example illustrates the different types of uncertainty that were considered in $IMPACT_{NCD}$. Let us assume that the annual risk for CHD is 5%. If we apply this risk to all individuals and randomly draw from a Bernoulli distribution with p = 5% to select those who will manifest CHD, we only consider stochastic uncertainty. If we allow the annual risk for CHD to be conditional on individual characteristics (i.e. age, sex, exposure to risk factors), then individual heterogeneity is considered. Finally, when the uncertainty of the relative risks due to sampling errors is considered in the estimation of the annual risk for CHD, the parameter uncertainty is considered. From these three types of uncertainty, only the parameter uncertainty can be reduced from better studies in the future.

Due to lack of information and for computational efficiency, not all three types of uncertainty are considered in every step (Figure S1) of $IMPACT_{NCD}$. Specifically, stochastic uncertainty is included in every step, individual heterogeneity in every step except 1 and 4 and parameter uncertainty in step 5. Of course, parameter uncertainty of scenario targets are also estimated in steps 2 and 3.

The structure of the model is grounded on fundamental epidemiological ideas and well-established causal pathways; therefore, we considered this type of uncertainty relatively small and did not study it. However, mortality from each of the modelled diseases and any-other cause (steps 6 and 7) is calculated serially, one modelled disease at a time. To avoid bias that this approach might introduce, the order of the modelled diseases in each mortality estimation is randomised.

---

* We assumed lognormal distributions for relative risks and hazard ratios, normal distributions for coefficients of regression equations, and PERT distributions for scenario-specific and other parameters. Specifically for relative risks and hazard ratios, the distributions were bounded above 1 when the mean was above 1 and vice versa.

† Individual life-course trajectories however, are not. The same normotensive individual may evolve and develop hypertension under scenario 'A' but not under scenario 'B' due to chance, and not as a direct effect of the scenarios.

From our experience in communicating our results to policy makers and researchers, we realised that they tend to misinterpret 95% UIs as 95% confidence intervals (CI) and overlapping UIs as ‘evidence against statistical significance’. This does not apply in our model because the scenarios share common sources of uncertainty as explained above; therefore, scenarios are not independent. We decided to present medians and interquartile ranges (IQRs) exactly to avoid this misunderstanding with UIs and CIs. We hope that readers will mentally visualise the distribution from medians and IQRs rather than attempt to apply frequentist statistical inference and hypothesis testing rules, which do not apply in this particular situation. In any case, all our output distributions were approximately normal and their standard deviation can be approximated by dividing IQR with 1.35. Then, z scores can be used to approximate any probability of UI.

CHAPTER 6. EQUITY METRICS

Absolute and relative equity slope index

The 'absolute equity slope index' and the 'relative equity slope index' are two regression-based metrics, to measure the impact of the modelled interventions on absolute and relative socioeconomic health inequalities. They are inspired by the slope index of inequality (SII) and the relative index of inequality (RII);[62] however, instead of directly measuring inequalities in a population, like SII and RII do, they measure the impact of an intervention to existing inequalities.

The basic principles of the metrics are illustrated in this simplified example. Let us consider the simple example of a population that consists of only two mutually exclusive and same-sized socioeconomic groups, the 'deprived' and the 'affluent'. The two groups experience different incidence of a disease; supposedly, 50 and 10 incident cases among the deprived and the affluent, respectively, every year. Hence, the absolute socioeconomic inequality for disease incidence is 50 – 10 = 40 cases and the relative socioeconomic inequality is 50 / 10 = 5. If a hypothetical intervention 'A' prevents the same number of cases in both groups, absolute inequality will remain stable. Similarly, if intervention 'A' prevents more cases in the affluent group, absolute inequality will increase and vice versa. For relative inequality to remain stable, the decrease in cases need to be proportional to the observed number of cases. For example, a hypothetical intervention 'B' that reduces 10% of cases in each group will have no effect on relative inequality. If the proportional reduction is higher in the affluent group compared to the deprived, then relative inequality will increase and vice versa.

As in many real-world examples, IMPACT$_{\textbf{NCD}}$ uses QIMD to classify population in five socioeconomic groups of unequal sizes. In this case, SII and RII can be used to measure absolute and relative socioeconomic inequalities in health, respectively. The same principles about intervention effectiveness and inequalities described in the previous paragraph, also apply here. If an intervention prevents equal number of cases in all QIMD groups SII will remain unchanged, while if the proportional reductions of cases in all QIMD groups are equal, RII will remain unchanged.[*] Inspired by SII and RII, the absolute equity slope index is the slope of the regression line fitted in the number of cases prevented or postponed by an intervention (dependent variable), on ridit scores[63] of QIMD (independent variable). Ridit scores reflect the average cumulative frequency of each QIMD group[†]. As in SII and RII they are used to account for the different sizes of each QIMD group (the distribution

---

[*] Assuming that the deaths prevented by the intervention does not change the relative size of the socioeconomic groups.

[†] So, if in QIMD 1,2,3,4 and 5 areas live 14%, 22%, 22%, 24% and 18% of the population respectively, the cumulative frequency is 14%, 36%, 58%, 82% and 100% and the rigid scores are 0+0.14/2 = 0.07, (0.14+0.36)/2 = 0.25, (0.36+0.58)/2 = 0.45, (0.58+0.82)/2 = 0.7 and (0.82+1)/2 = 0.91

of inequality), and allow for comparisons between populations. A positive slope means that the intervention prevents more cases in the more deprived QIMD groups and reduces absolute inequality in the population, and vice versa. The magnitude of the slope is proportional to the reduction in absolute inequality. The relative equity slope index is constructed and interpreted similarly, except that the proportion of cases prevented or postponed over the total cases in each socioeconomic group is the independent variable, and it measures the effect on relative inequality.

Equity summary chart

The equity summary chart presents, in a simple two-dimensional chart, the impact of the interventions on disease incidence, and absolute and relative socioeconomic inequality. The horizontal axis represents the number of cases prevented or postponed and the vertical axis represents the decrease (or increase) in absolute inequality. An 'equity' curve (or line) divides the graph in two parts. Interventions above the equity curve decrease relative inequality and interventions below it increase relative inequality. The underlying assumption of the equity summary chart and the equity curve is that for a given overall reduction of disease burden in the whole population, attributable to an intervention, there is one and only one way to distribute the reduction among the socioeconomic groups that can reduce absolute socioeconomic inequality and have no impact on relative socioeconomic inequality.

Let us consider the simple example of a population that consists of only two mutually exclusive socioeconomic groups, the 'deprived' and the 'affluent', with different disease incidence. Then the disease incident cases of the whole population $I = I_{deprived} + I_{affluent}$, where $I_{deprived}$ is the cases in the 'deprived' group and $I_{affluent}$ is the cases in the 'affluent' group. Also by definition, absolute inequality $E_{abs} = I_{deprived} - I_{affluent}$, and relative inequality $E_{rel} = I_{deprived} / I_{affluent}$.

For a given overall reduction in disease incident cases across groups (ΔI), the distribution of the reduction among the 2 groups can be described as $I_{deprived} - a$ and $I_{affluent} - b$, where $a + b = \Delta I$. The post intervention absolute and relative inequality is $E'_{abs} = I_{deprived} - I_{affluent} - a + b$ and $E'_{rel} = (I_{deprived} - a)/(I_{affluent} - b)$, respectively. Assuming that after the intervention the two subgroups remain approximately of equal size, for $E_{rel} = E'_{rel}$ then it can be shown that $a = E_{rel} * b$ and $E_{abs} - E'_{abs} = \Delta I * (E_{rel} - 1)/(E_{rel} + 1)$, which on the equity summary chart is a line that represents the 'equity line' for this population (Figure S19). Interventions above the equity line decrease relative socioeconomic inequalities and interventions bellow the line increase it. Moreover, the vertical distance from the equity line is proportional to the impact of the intervention on relative socioeconomic inequalities.

The generalisation of the previous example to populations with more than two levels of socioeconomic deprivation and unequal sizes of the socioeconomic groups is explained below. Complex measures to summarise absolute and relative socioeconomic inequalities already exist for this situation[62,64]. We will use slope index of inequality (SII) to summarise absolute inequality in the population and relative index of inequality (RII) to summarise relative inequality. They are regression-based approaches with the first, representing the health measure difference between the most and least deprived individuals and the second, the health measure ratio between the most and least deprived individuals.

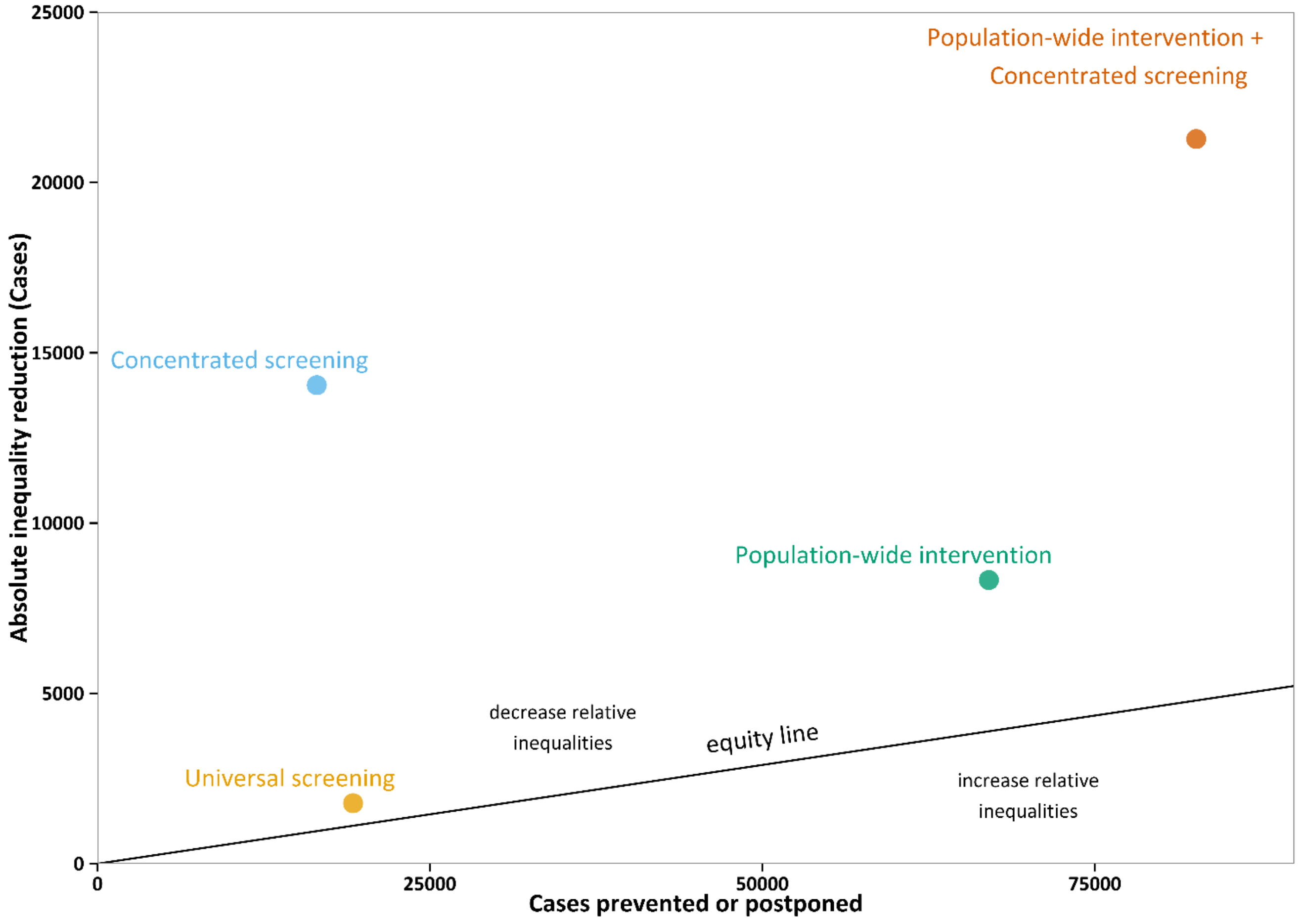

Figure S19 Simplified summary equity chart assuming only two mutually exclusive socioeconomic groups and assuming that interventions do not alter the size of each group.

Hence, using the notation of the previous example $SII = I_{deprived} - I_{affluent}$ and $RII = I_{deprived} / I_{affluent}$, where this time $I_{deprived}$ and $I_{affluent}$ are not directly observed but are extrapolated from the linear regression that was used for the SII. Therefore, incident cases I for the whole population are not equal to $I_{deprived} + I_{affluent}$. From these formulas it can be shown that

$I_{affluent} = SII/(RII - 1)$ and $I_{deprived} = RII * SII/(RII - 1)$. For the incident cases I of the whole population holds that $I = r * I_{deprived} + (1 - r) * I_{affluent}$, where $0 \leq r \leq 1$ and r depends on the distribution of inequality in the population. From the previous formulas it can be shown that $r = I/SII - 1/(RII - 1)$.

After the intervention, the new incidence $I' = r' * I'_{deprived} + (1 - r') * I'_{affluent}$ and will result in a new SII' and RII'. Because r' is dependent on the impact of intervention on the different socioeconomic groups, the 'equity line' cannot be defined as in the previous simplified example. However for a given intervention, r' can be estimated and assuming there is an SII'' for $RII = RII'$. It can be shown that $SII'' = I' * (RII - 1)/(r' * (RII - 1) + 1)$ and SII'' increases monotonically as RII increases. Therefore, $SII - SII''$ decreases monotonically as RII increases.

The horizontal axis of the equity summary chart represents $\Delta I = I - I' = Cases\ prevented\ or\ postponed$ and *the vertical axis* $\Delta \mathrm{SII} = \mathrm{SII} - \mathrm{SII}'$*. The graph in the main text was created by plotting* ΔI and $\Delta \mathrm{SII}$ *for each scenario (in year 2015). Then each scenario was plotted on the graph.* For each scenario ($\Delta \mathrm{I}$, $SII - SII''$) was also plotted. To improve readability of the graph and for presentation purposes only, a constrained b-spline curve was fitted to them to represent the 'equity' curve. Scenarios above the equity curve decrease relative inequality and scenarios below the equity curve increase it. The vertical distance from the curve, roughly represents the impact of the scenario on relative inequality. Consequently, the health equity impact chart presents on a two axes chart, the impact of the intervention on CVD incidence, absolute and relative inequality.

CHAPTER 7. VALIDATION

In this chapter, we first present the internal validation of the synthetic population and the risk factor trends, as an evidence that the synthetic population used in $IMPACT_{NCD}$ was similar to English population. Then, we present the predictive validation of $IMPACT_{NCD}$ by comparing observed to predicted mortality rates for years 2006 to 2013 by age group, sex, QIMD, and modelled disease. Specifically for the predictive validation, $IMPACT_{NCD}$ was calibrated to data up to 2006. The only exception was the regression models that were used in for individual predictions of exposure to risk factors (steps 2 and 3 in Figure S1). These models were fitted in data from 2001 to 2012 as described before. This is appropriate, as the main use of $IMPACT_{NCD}$ is to translate changes in risk factor into changes in CVD[*] incidence and mortality, and not forecasting. Finally, we present the comparison of $IMPACT_{NCD}$ baseline scenario with BAMP, a Bayesian age-period-cohort model.[65]

Synthetic population validation

The following graphs compare a random sample of 400,000 synthetic individuals from the synthetic population to the original sample of HSE11 (n = 10,617). Mosaic plots[†] were used for the categorical variables and cumulative distribution plots were used for the continuous variables. Specifically in this document, the area of each tile of the mosaic plots is proportional to the proportion of each subgroup in the respective population. Only graphs that were relevant to the analysis for this study are presented here.

The graphs support the argument that the final synthetic population is close to reality, at least as it was captured through the HSE11, and are useful for the internal validation of the method. Alfons et al. used a statistical simulation approach to validate the process and showed that this method produces synthetic populations very similar to the original survey.[9] Of course, the method cannot overcome any limitations of the original survey, such as selection bias, or misclassification.

---

[*] Other non-communicable diseases will be included in the future.

[†] Mosaic plots are graphical representations of a contingency table of two or more categorical variables, using tiles with areas proportional to the frequencies in each cell of the table.[66]

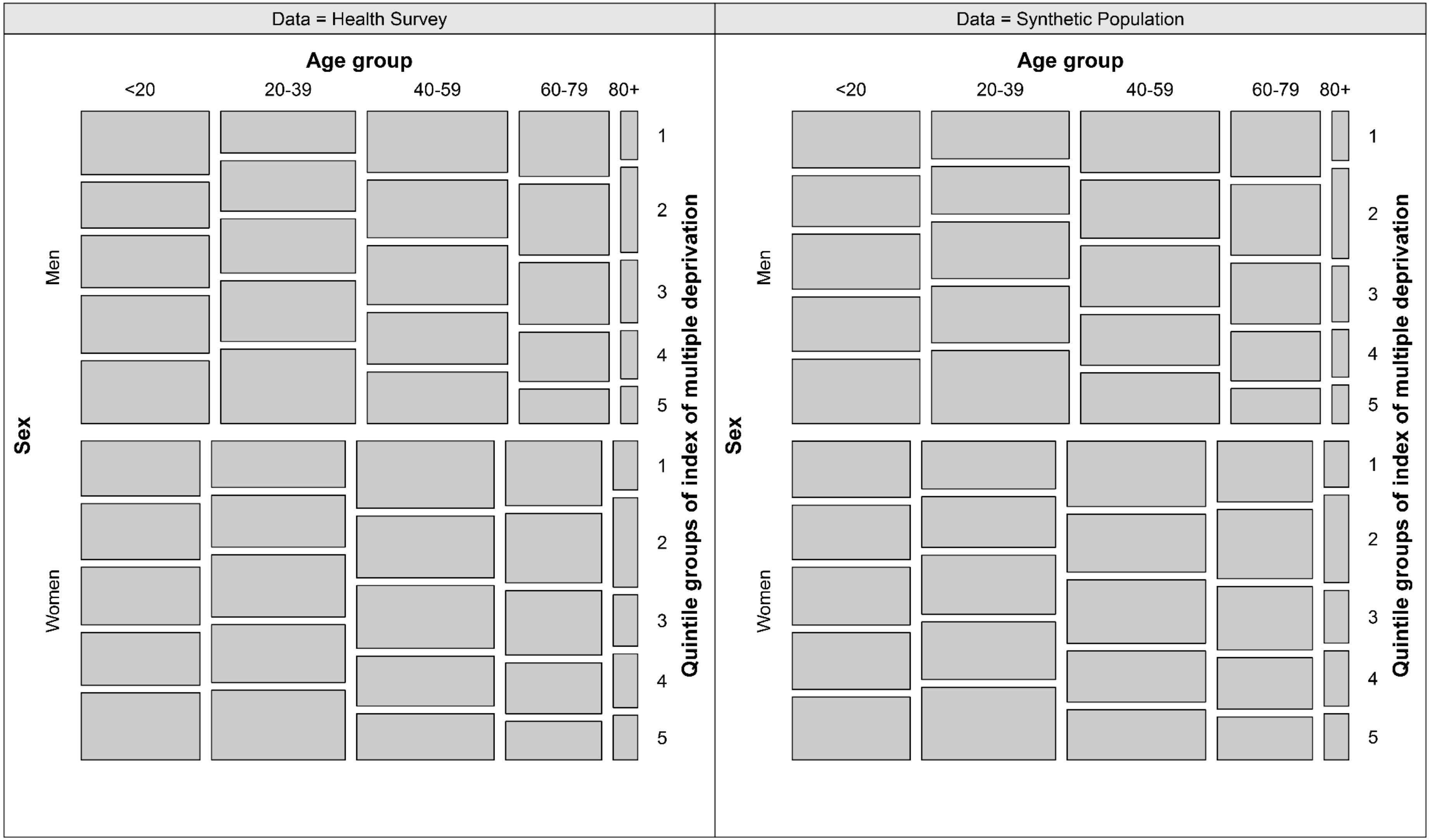

Figure S3 Comparison between the Health Survey for England 2011 (n = 10,617) and a random sample (n=400,000) from the synthetic population. Distribution of age group, sex and quintile groups of index of multiple deprivation (1=least deprived, 5=most deprived) is presented.

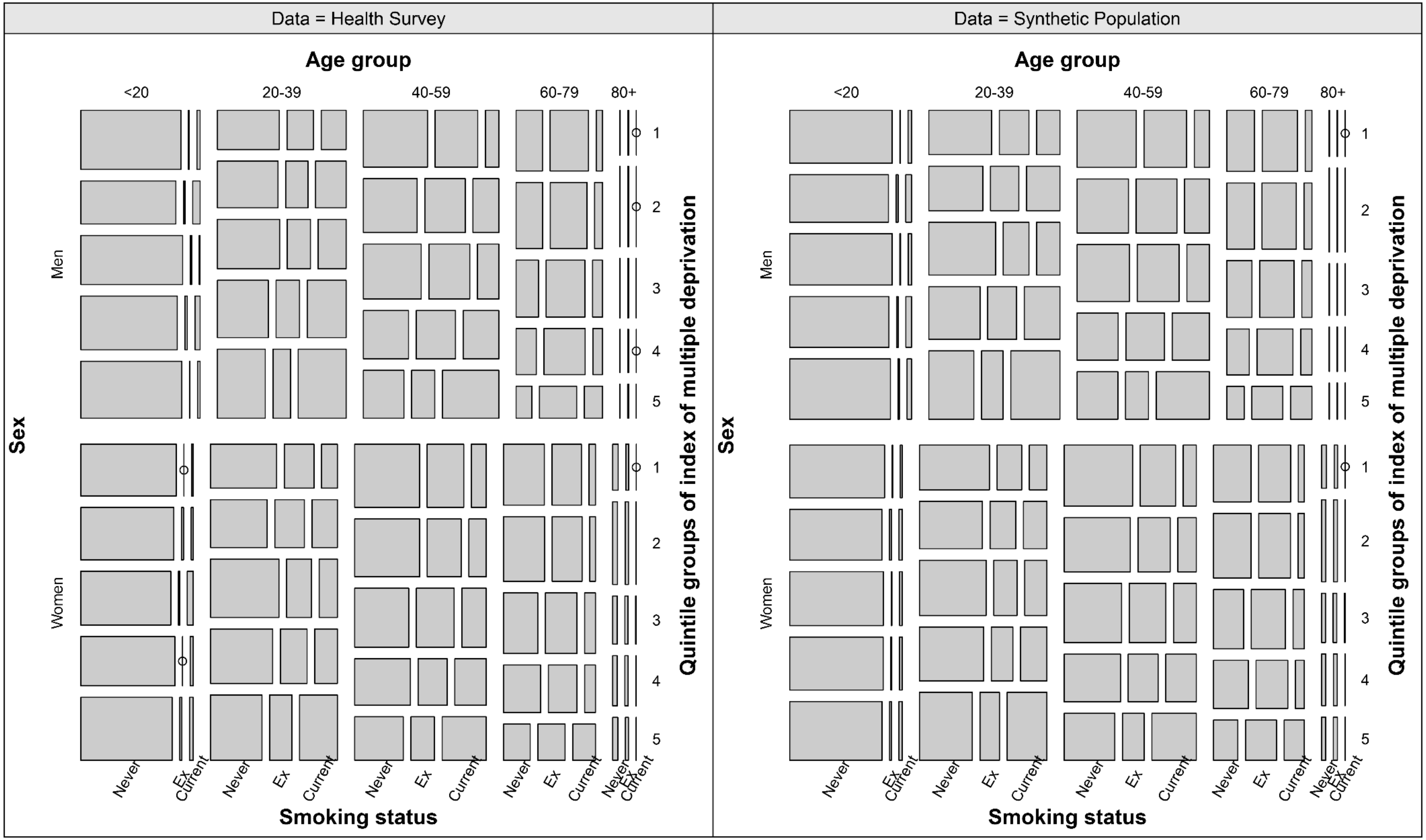

Figure S4 Comparison between the Health Survey for England 2011 (n = 10,617) and a random sample (n=400,000) from the synthetic population. Distribution of age group, sex, quintile groups of index of multiple deprivation (1=least deprived, 5=most deprived) and smoking status is presented. The small circles represent sub-groups with no participants. Their number was reduced in the synthetic population sample, highlighting the capability of the method to create individuals with traits not present in the original survey.

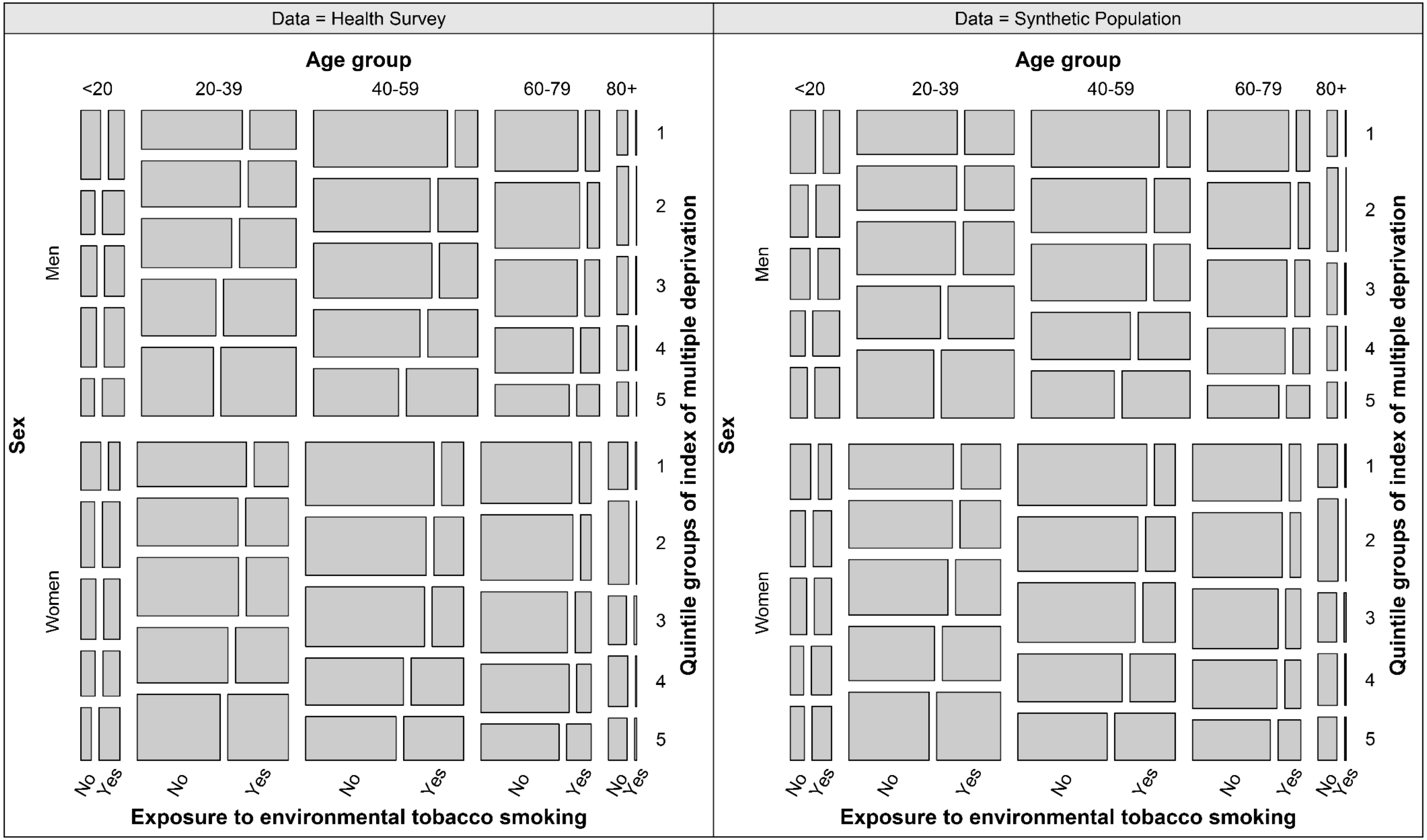

Figure S5 Comparison between the Health Survey for England 2011 (n = 10,617) and a random sample (n=400,000) from the synthetic population. Distribution of age group, sex, quintile groups of index of multiple deprivation (1=least deprived, 5=most deprived) and exposure to environmental tobacco is presented.

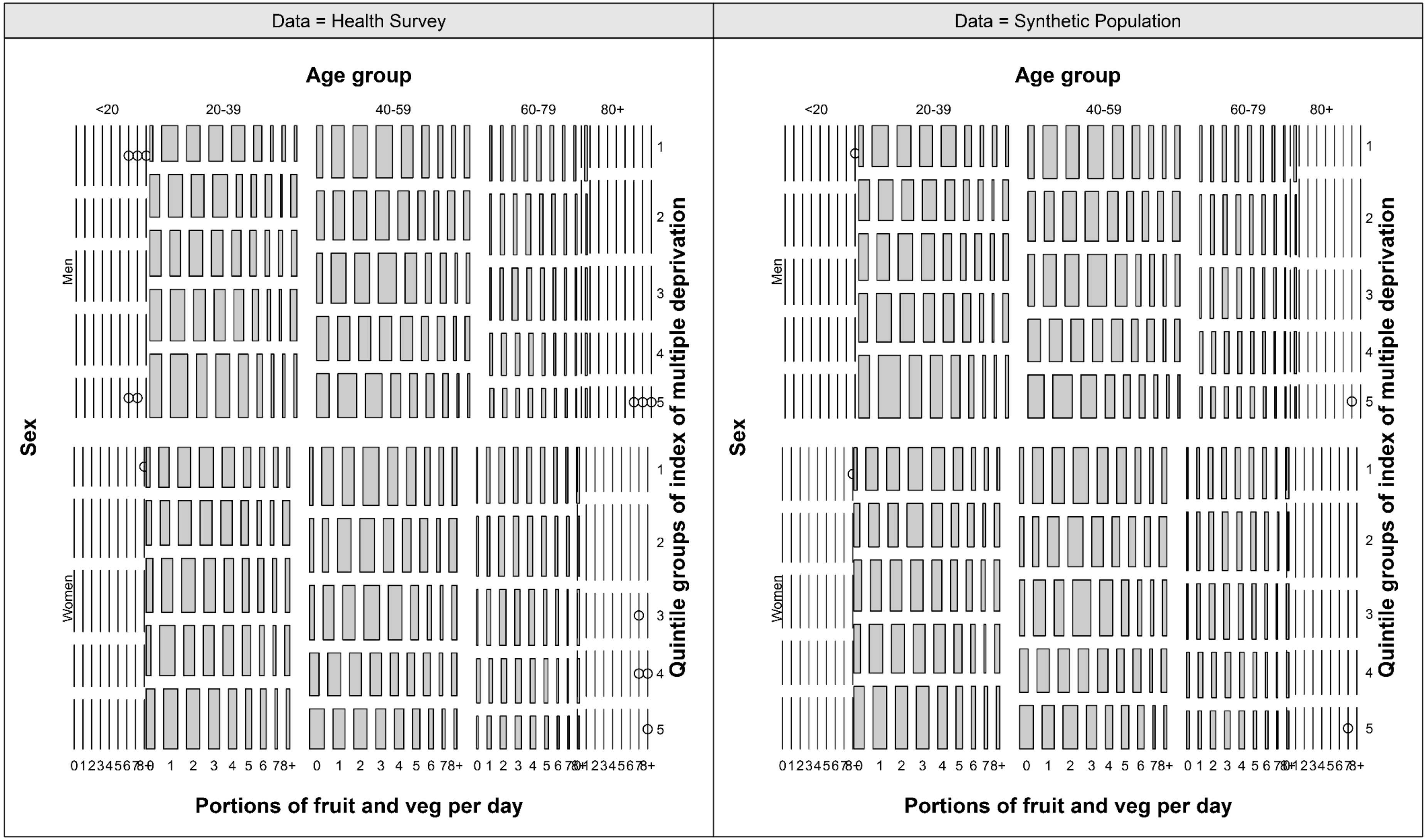

Figure S6 Comparison between the Health Survey for England 2011 (n = 10,617) and a random sample (n=400,000) from the synthetic population. Distribution of age group, sex, quintile groups of index of multiple deprivation (1=least deprived, 5=most deprived) and portions of fruit and vegetable consumed per day is presented.

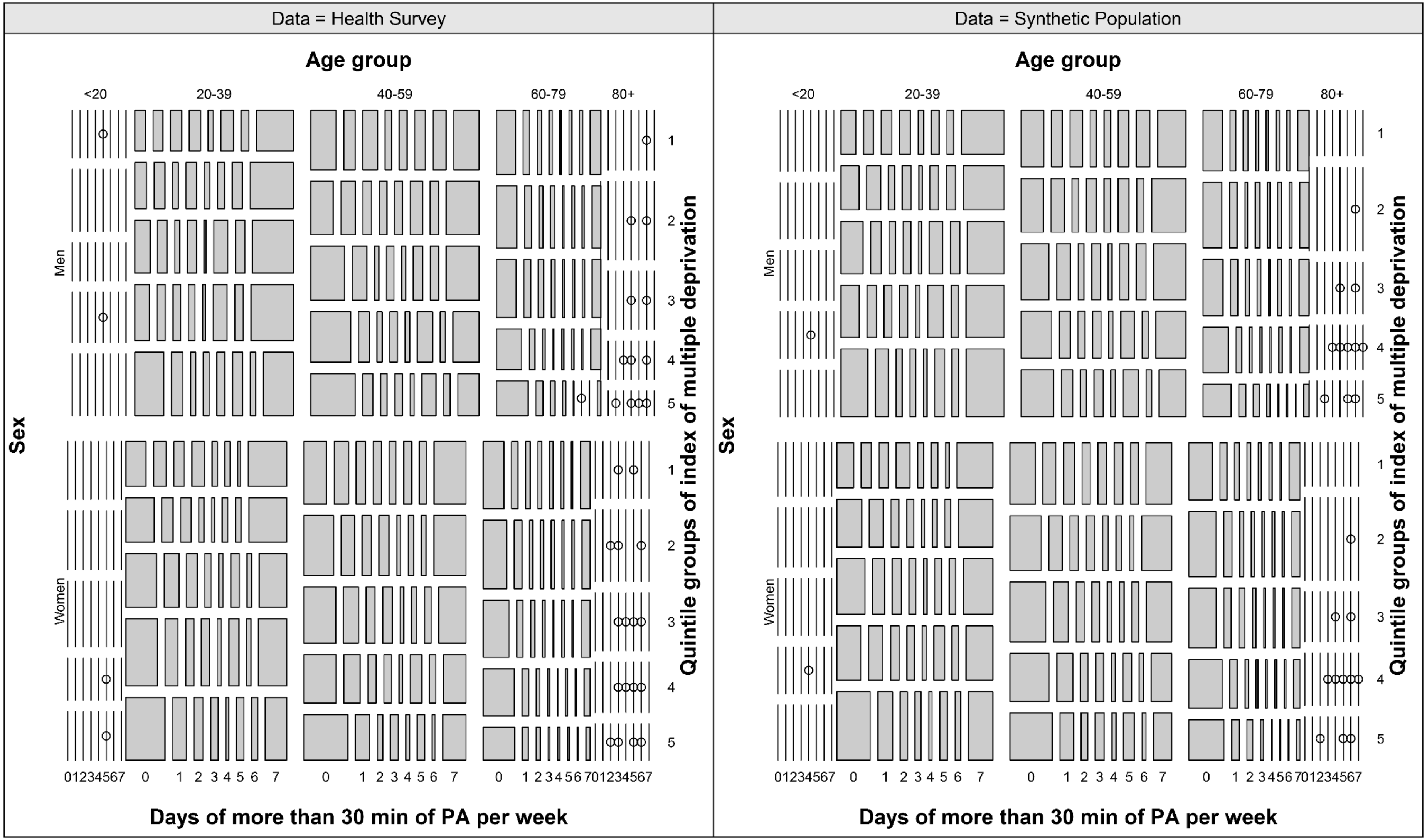

Figure S7 Comparison between the Health Survey for England 2012 (n = 10,333) and a random sample (n=400,000) from the synthetic population. Distribution of age group, sex, quintile groups of index of multiple deprivation (1=least deprived, 5=most deprived) and exposure to days of more than 30 min of physical activity (PA) per week is presented.

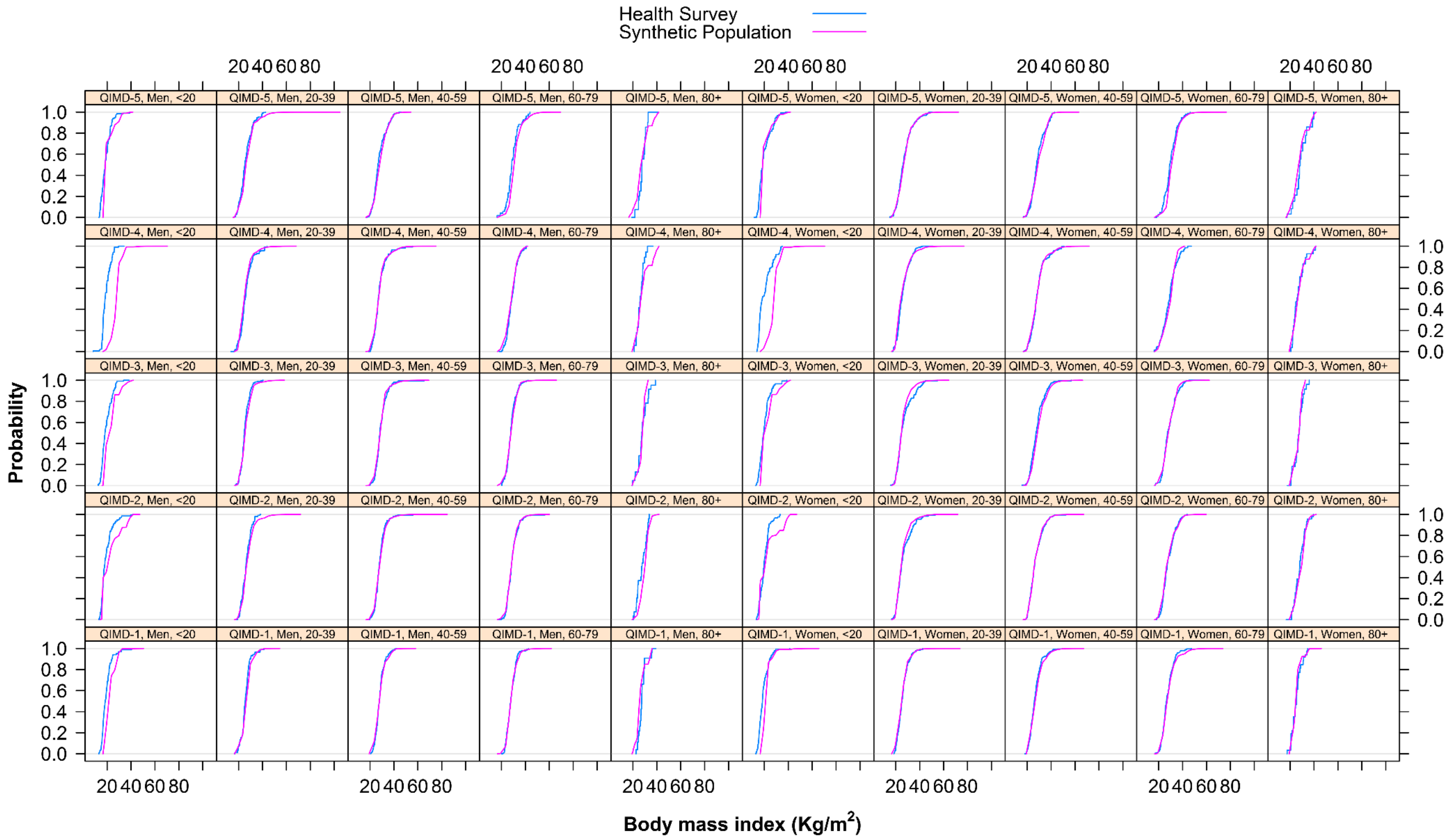

Figure S9 Comparison of body mass index cumulative distributions in Health Survey for England 2011 (n = 10,617) and a random sample (n=400,000) from the synthetic population. Each panel depicts a different subgroup of the population based on quintile groups of index of multiple deprivation (QIMD, 1=least deprived, 5=most deprived), sex and age group.

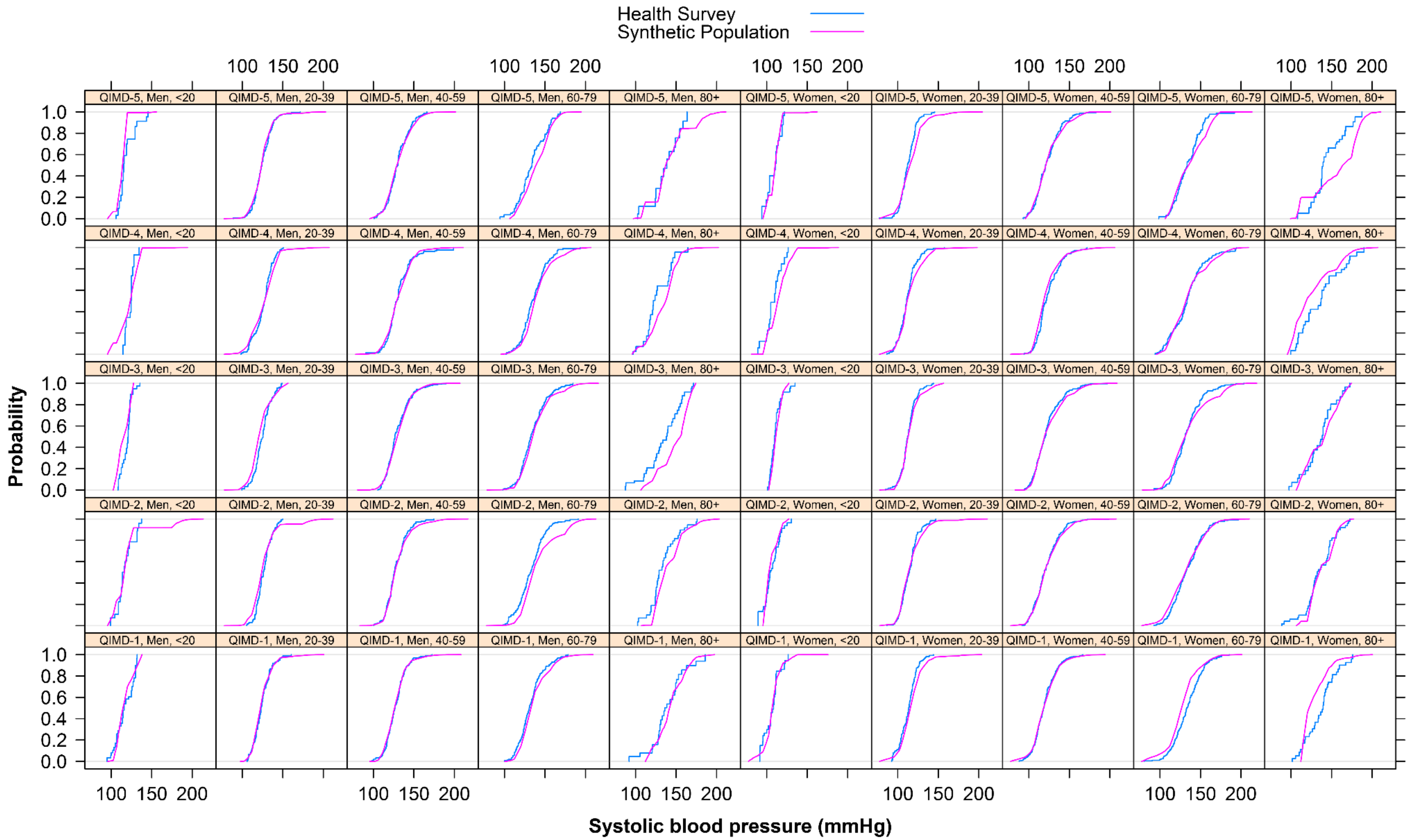

Figure S10 Comparison of systolic blood pressure cumulative distributions in Health Survey for England 2011 (n = 10,617) and a random sample (n=400,000) from the synthetic population. Each panel depicts a different subgroup of the population based on quintile groups of index of multiple deprivation (QIMD, 1=least deprived, 5=most deprived), sex and age group.

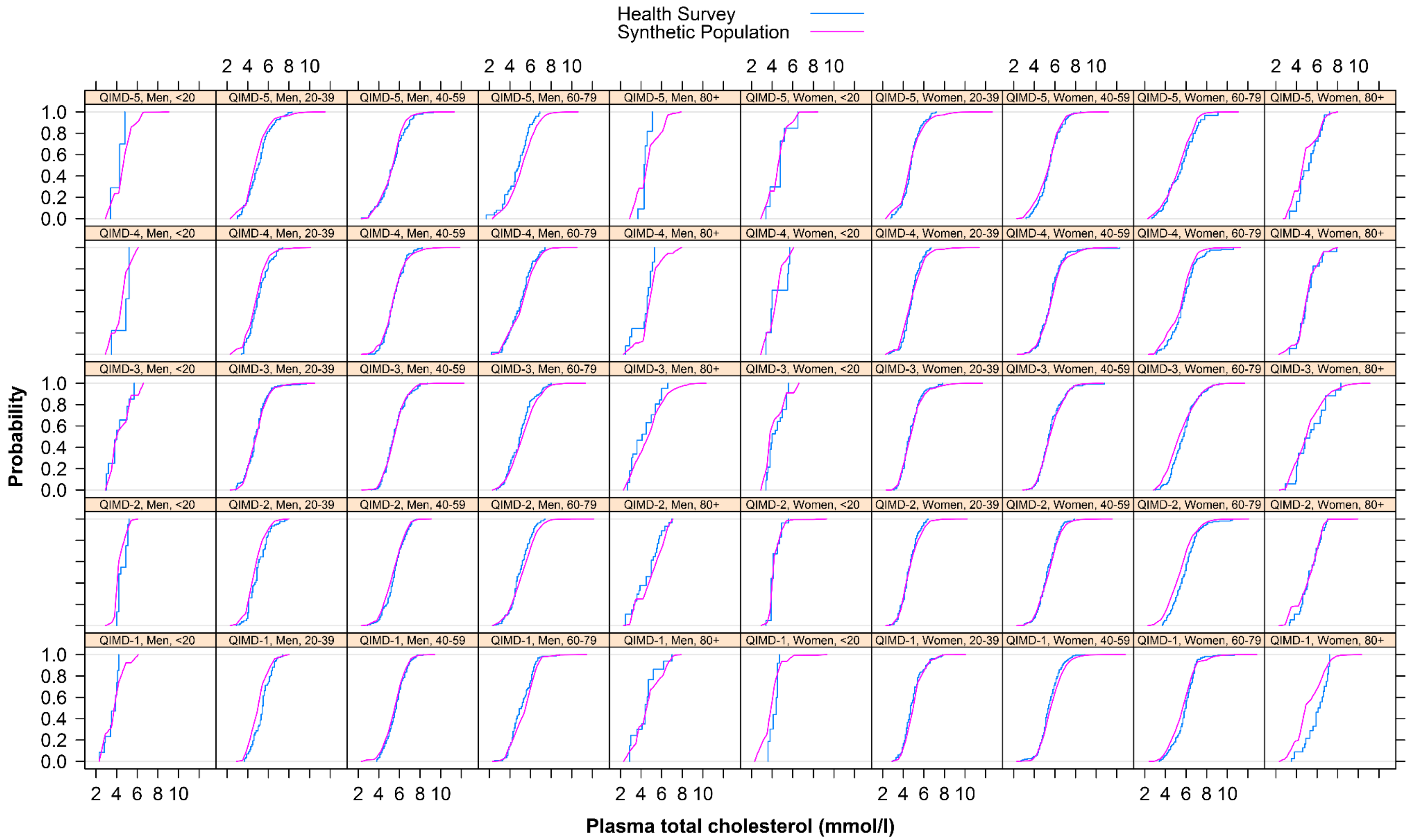

Figure S11 Comparison of plasma total cholesterol cumulative distributions in Health Survey for England 2011 (n = 10,617) and a random sample (n=400,000) from the synthetic population. Each panel depicts a different subgroup of the population based on quintile groups of index of multiple deprivation (QIMD, 1=least deprived, 5=most deprived), sex and age group.

Risk factor trends validation

Here we compare mean exposure of $\text{IMPACT}_{\text{NCD}}$ synthetic population to the observed exposure through relevant national representative surveys. We stratified by sex, age group and when data allowed by QIMD. Overall, the plots provide evidence that the regression models used (steps 2 and 3 Figure S1), have captured trends by age, sex and QIMD well enough.

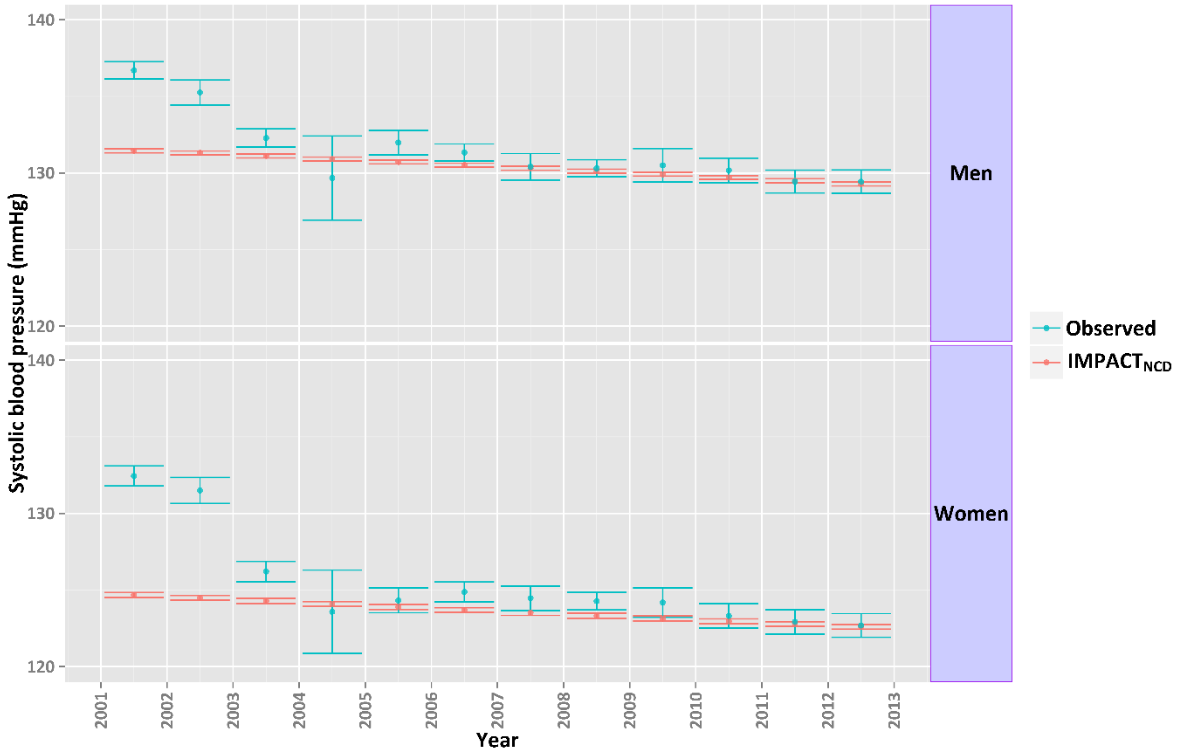

Figure S29 Mean systolic blood pressure for ages 30 – 84 between years 2001 and 2012. Observed in the population through Health Survey for England vs. $\text{IMPACT}_{\text{NCD}}$ synthetic population estimates. Error bars represent 95% confidence intervals of the mean.

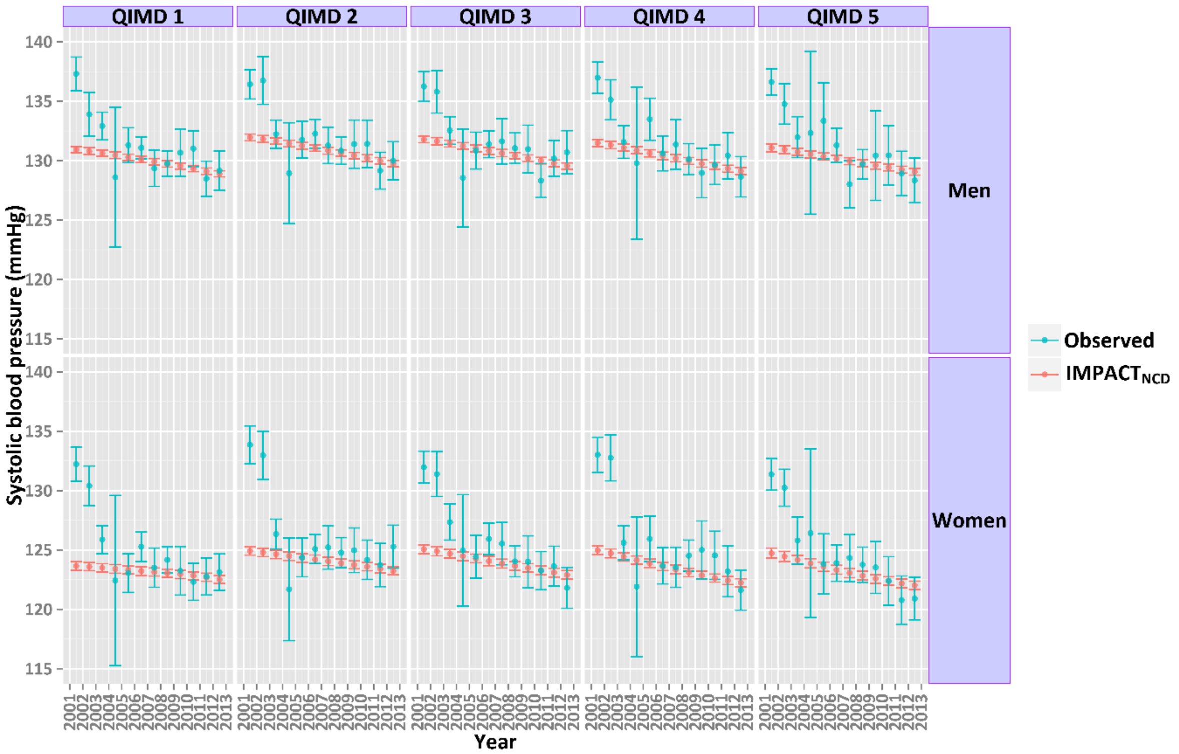

Figure S30 Mean systolic blood pressure for ages 30 – 84 by quintile group of index of multiple deprivation (QIMD, 1 = least deprived) between years 2001 and 2012. Observed in the population through Health Survey for England vs. IMPACT$_{NCD}$ synthetic population estimates. Error bars represent 95% confidence intervals of the mean.

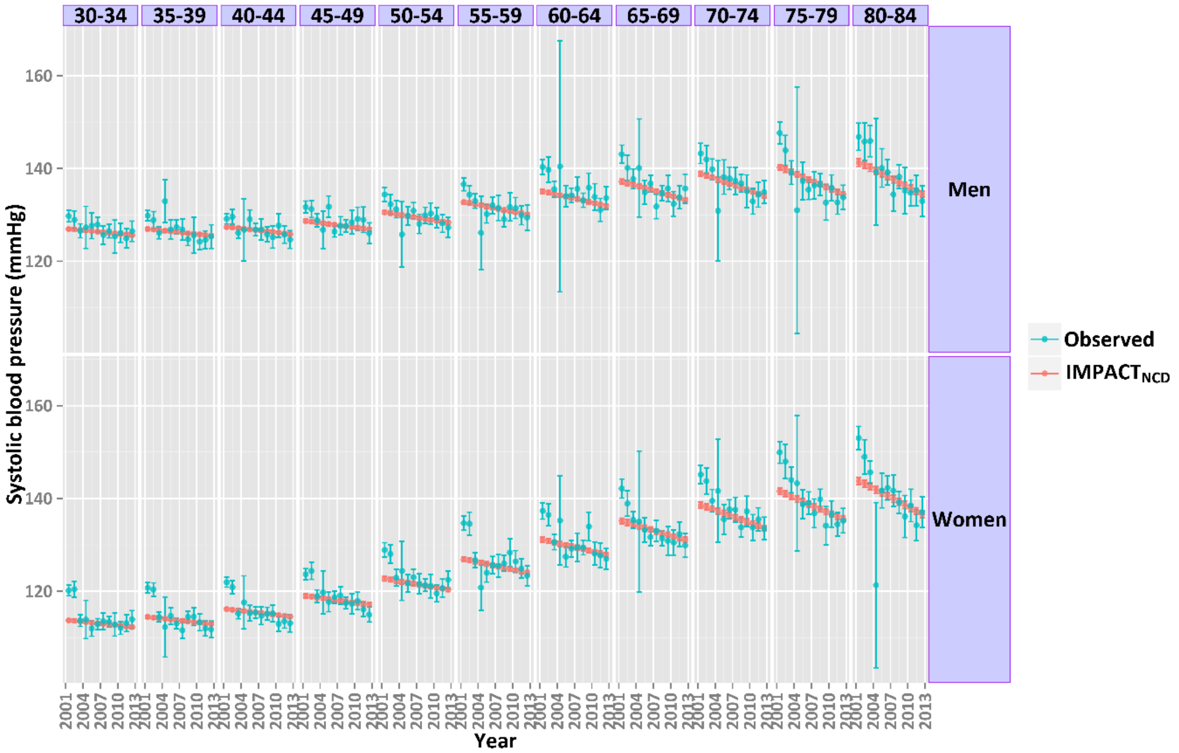

Figure S31 Mean systolic blood pressure for ages 30 – 84 by age group, between years 2001 and 2012. Observed in the population through Health Survey for England vs. IMPACT$_{NCD}$ synthetic population estimates. Error bars represent 95% confidence intervals of the mean.

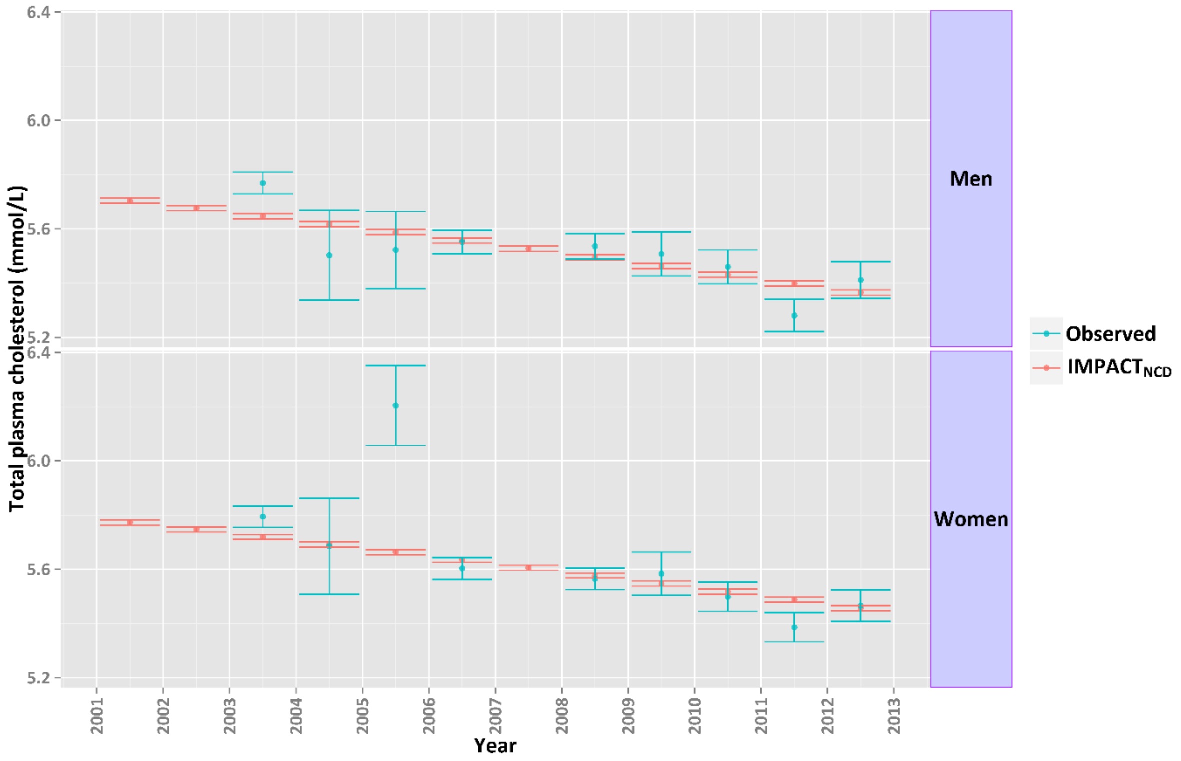

Figure S32 Mean total plasma cholesterol for ages 30 – 84 between years 2001 and 2012. Observed in the population through Health Survey for England vs. IMPACT$_{NCD}$ synthetic population estimates. Error bars represent 95% confidence intervals of the mean.

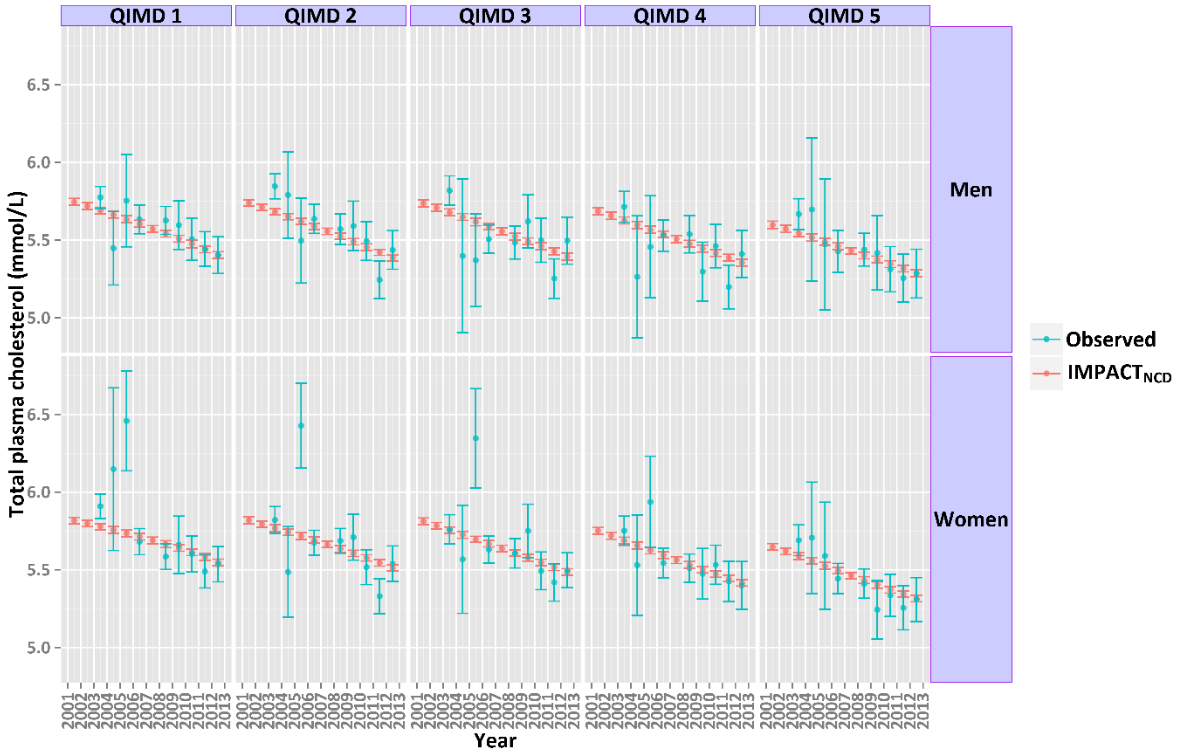

Figure S33 Mean total plasma cholesterol for ages 30 – 84 by quintile group of index of multiple deprivation (QIMD, 1 = least deprived) between years 2001 and 2012. Observed in the population through Health Survey for England vs. IMPACT$_{NCD}$ synthetic population estimates. Error bars represent 95% confidence intervals of the mean.

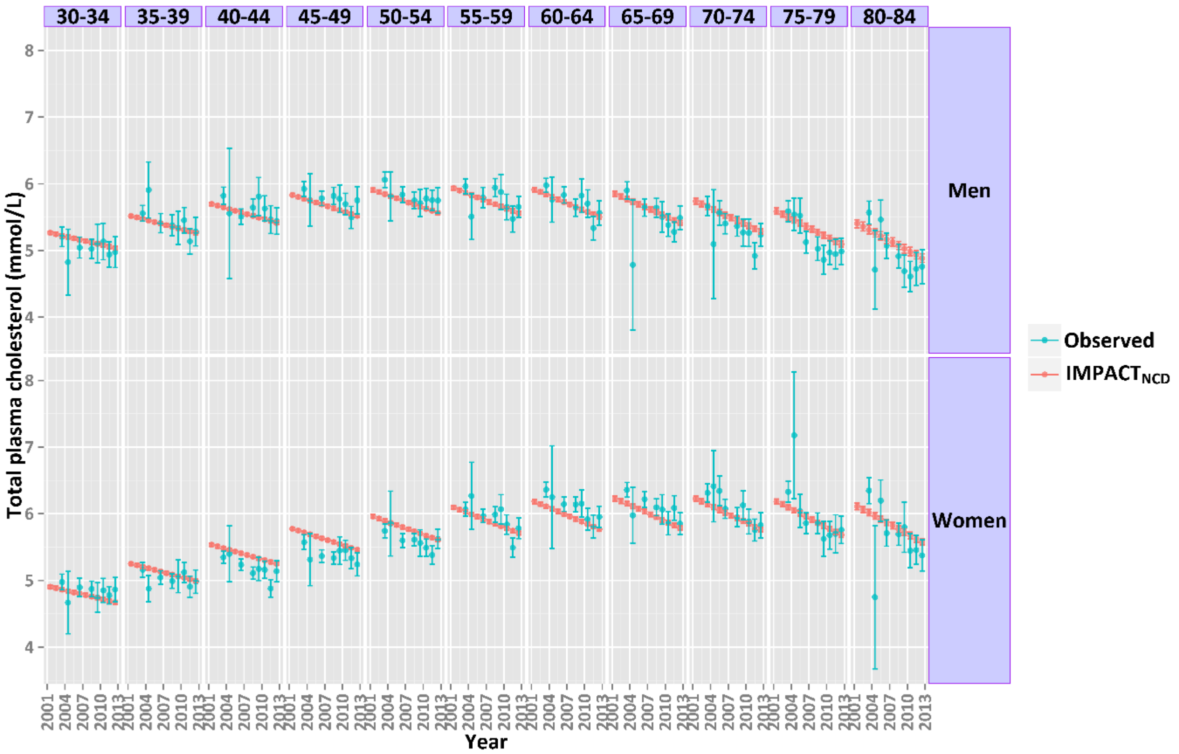

Figure S34 Mean total plasma cholesterol for ages 30 – 84 by age group, between years 2001 and 2012. Observed in the population through Health Survey for England vs. IMPACT$_{NCD}$ synthetic population estimates. Error bars represent 95% confidence intervals of the mean.

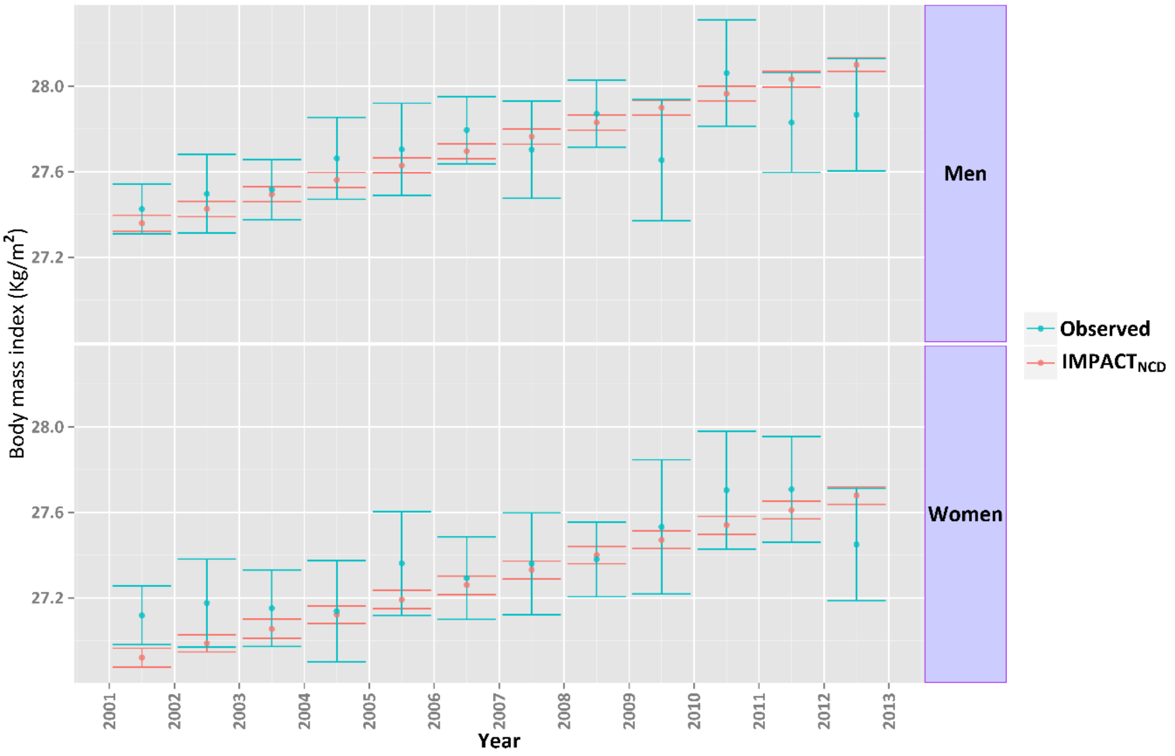

Figure S35 Mean body mass index for ages 30 – 84 between years 2001 and 2012. Observed in the population through Health Survey for England vs. IMPACT$_{NCD}$ synthetic population estimates. Error bars represent 95% confidence intervals of the mean.

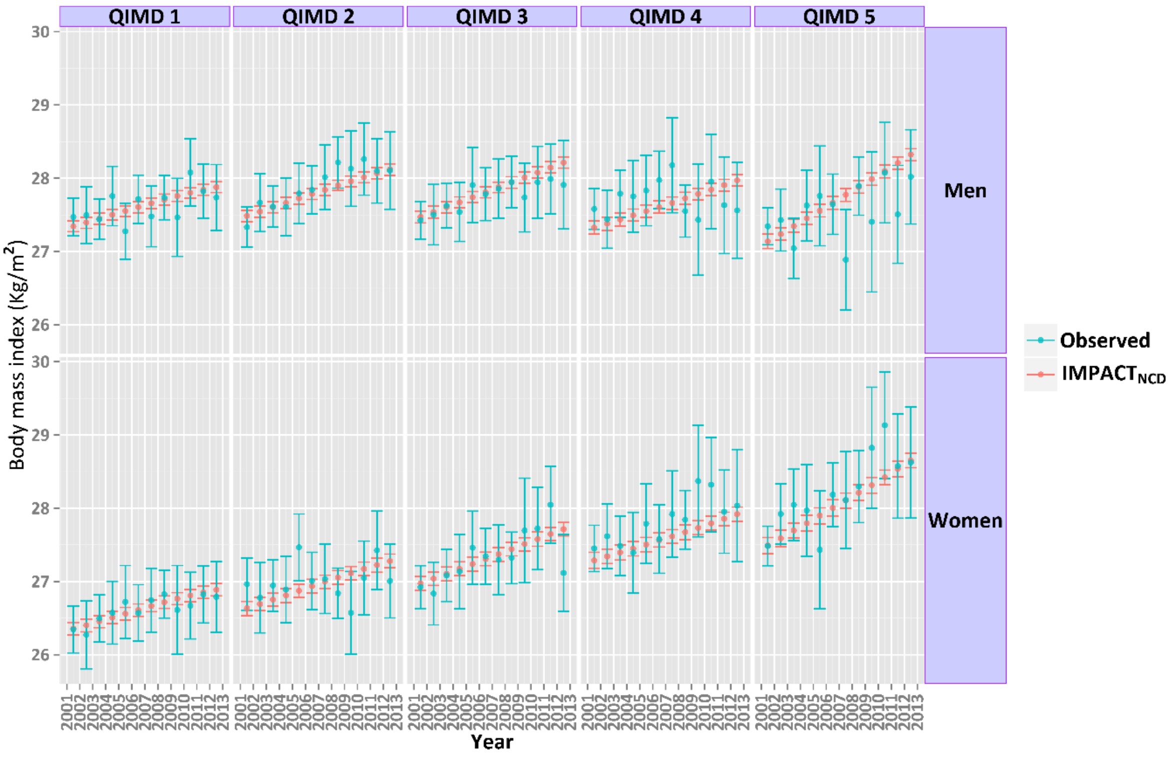

Figure S36 Mean body mass index for ages 30 – 84 by quintile group of index of multiple deprivation (QIMD, 1 = least deprived) between years 2001 and 2012. Observed in the population through Health Survey for England vs. $IMPACT_{NCD}$ synthetic population estimates. Error bars represent 95% confidence intervals of the mean.

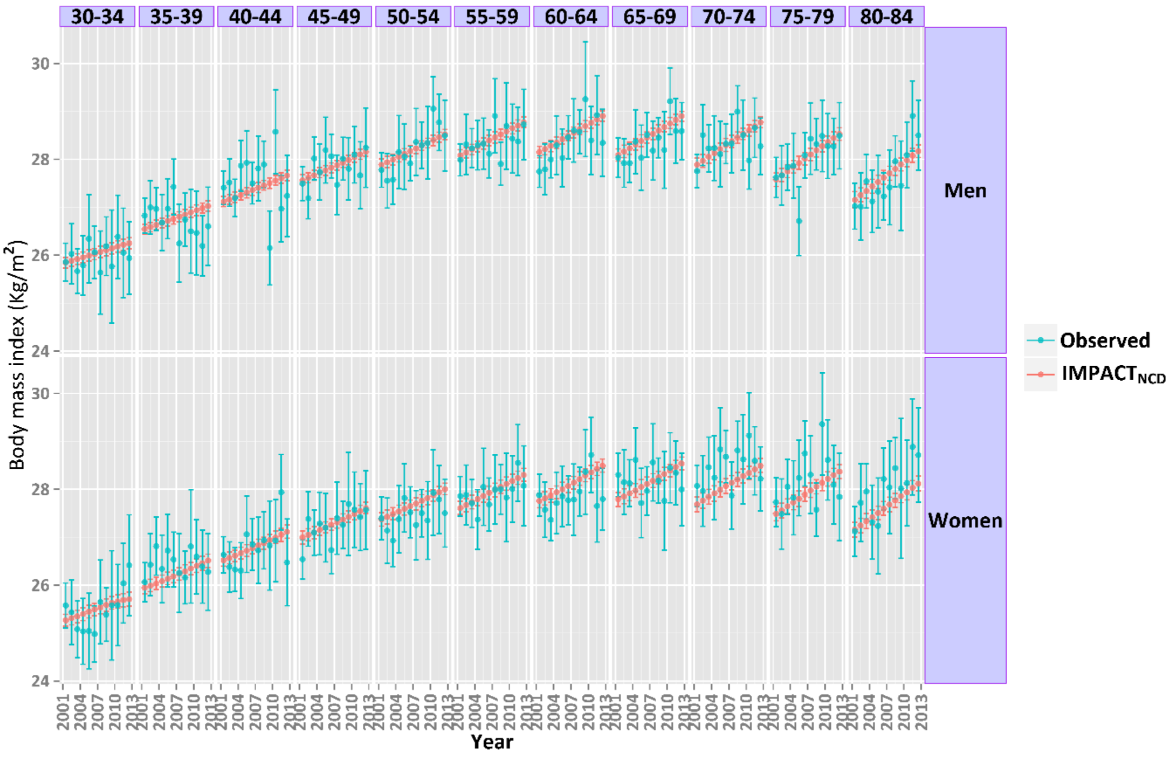

Figure S37 Mean body mass index for ages 30 – 84 by age group between years 2001 and 2012. Observed in the population through Health Survey for England vs. $IMPACT_{NCD}$ synthetic population estimates. Error bars represent 95% confidence intervals of the mean.

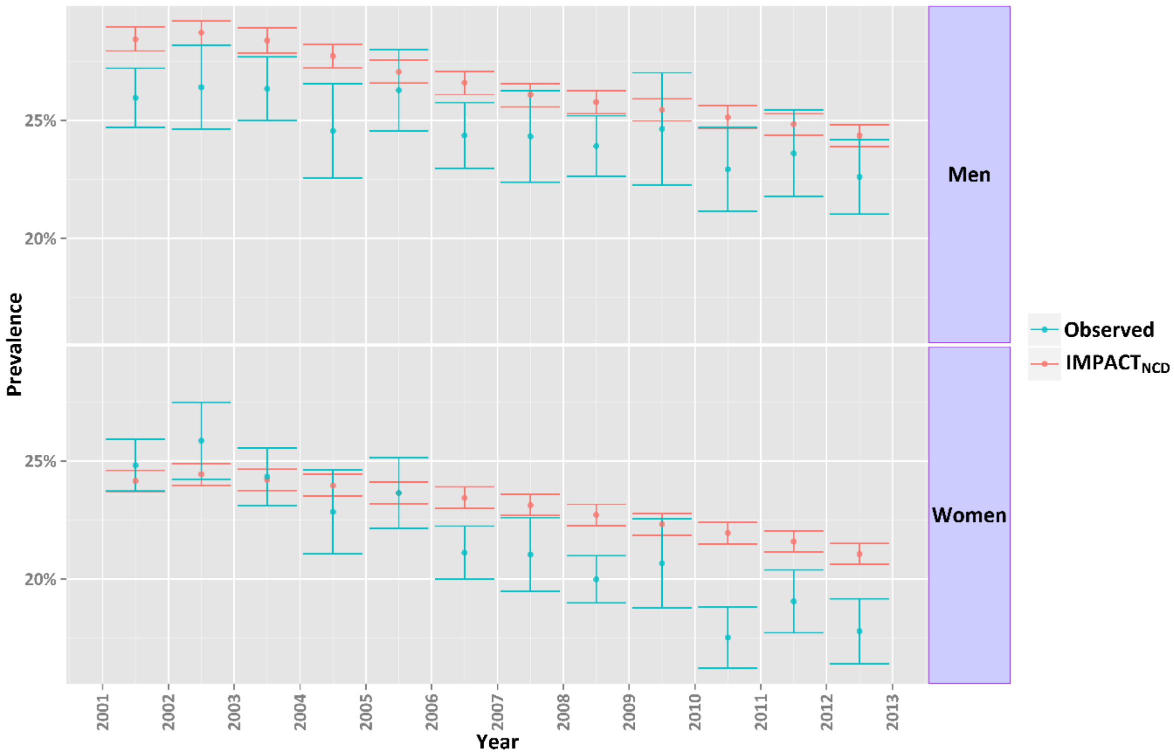

Figure S38 Smoking prevalence for ages 30 – 84 between years 2001 and 2012. Observed in the population through Health Survey for England vs. $IMPACT_{NCD}$ synthetic population estimates. Error bars represent 95% confidence intervals of the mean.

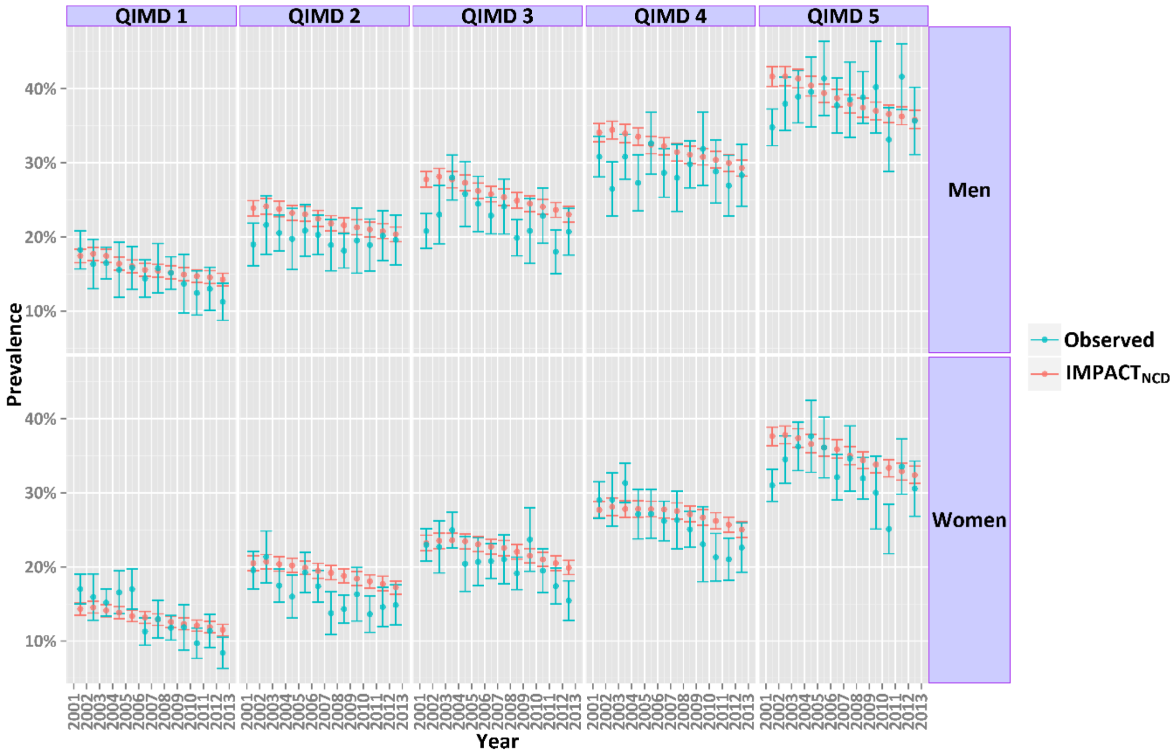

Figure S39 Smoking prevalence for ages 30 – 84 by quintile group of index of multiple deprivation (QIMD, 1 = least deprived) between years 2001 and 2012. Observed in the population through Health Survey for England vs. $IMPACT_{NCD}$ synthetic population estimates. Error bars represent 95% confidence intervals of the mean.

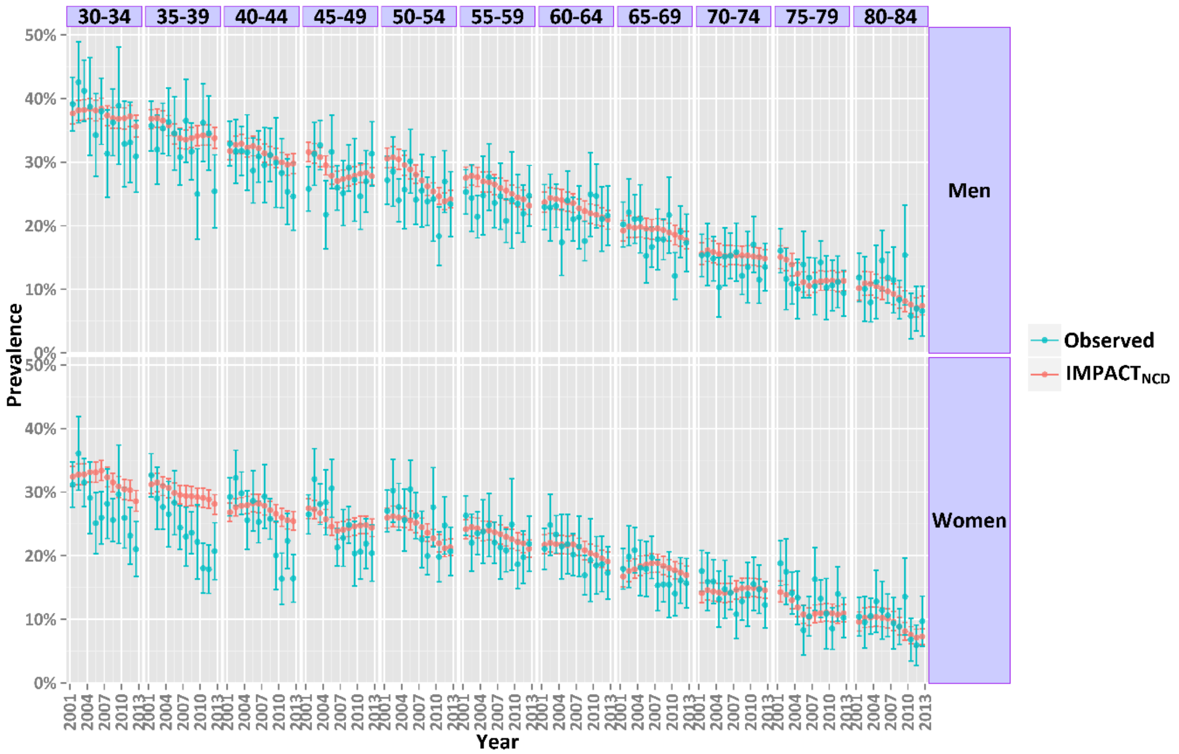

Figure S40 Smoking prevalence for ages 30 – 84 by age group between years 2001 and 2012. Observed in the population through Health Survey for England vs. IMPACT$_{NCD}$ synthetic population estimates. Error bars represent 95% confidence intervals of the mean.

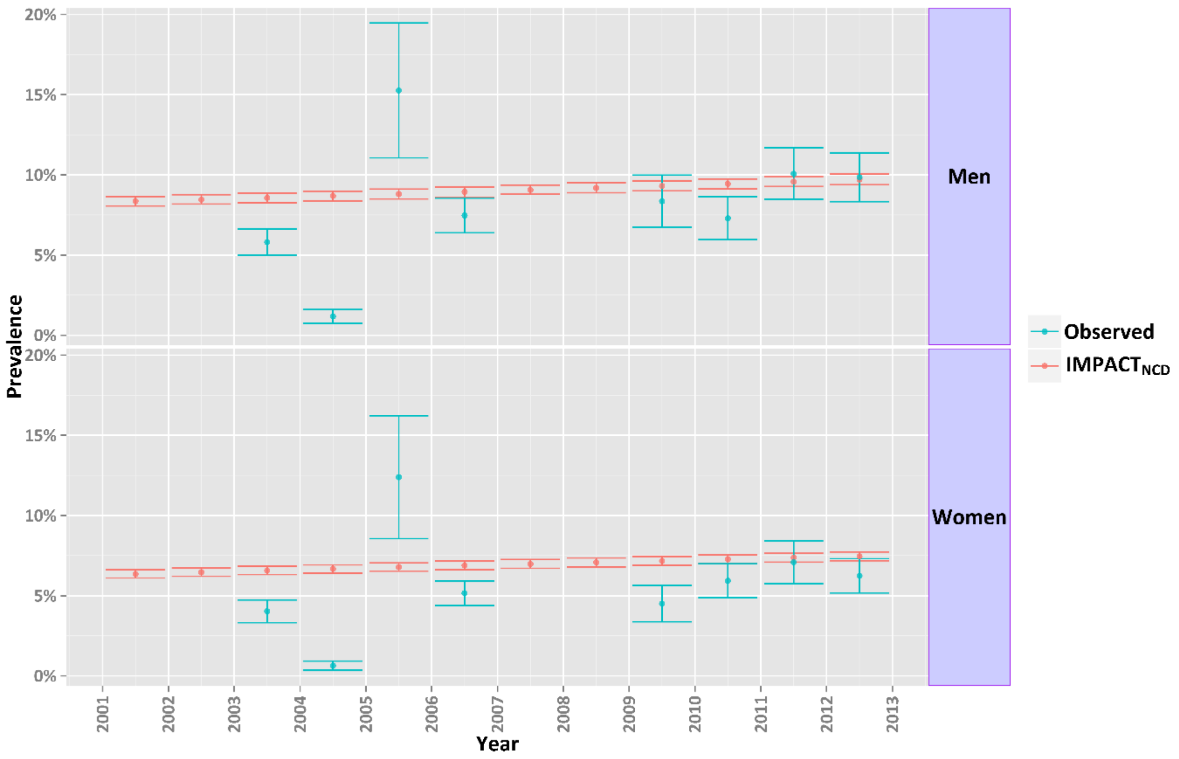

Figure S41 Diabetes mellitus prevalence for ages 30 – 84 between years 2001 and 2012. Observed in the population through Health Survey for England vs. IMPACT$_{NCD}$ synthetic population estimates. Error bars represent 95% confidence intervals of the mean.

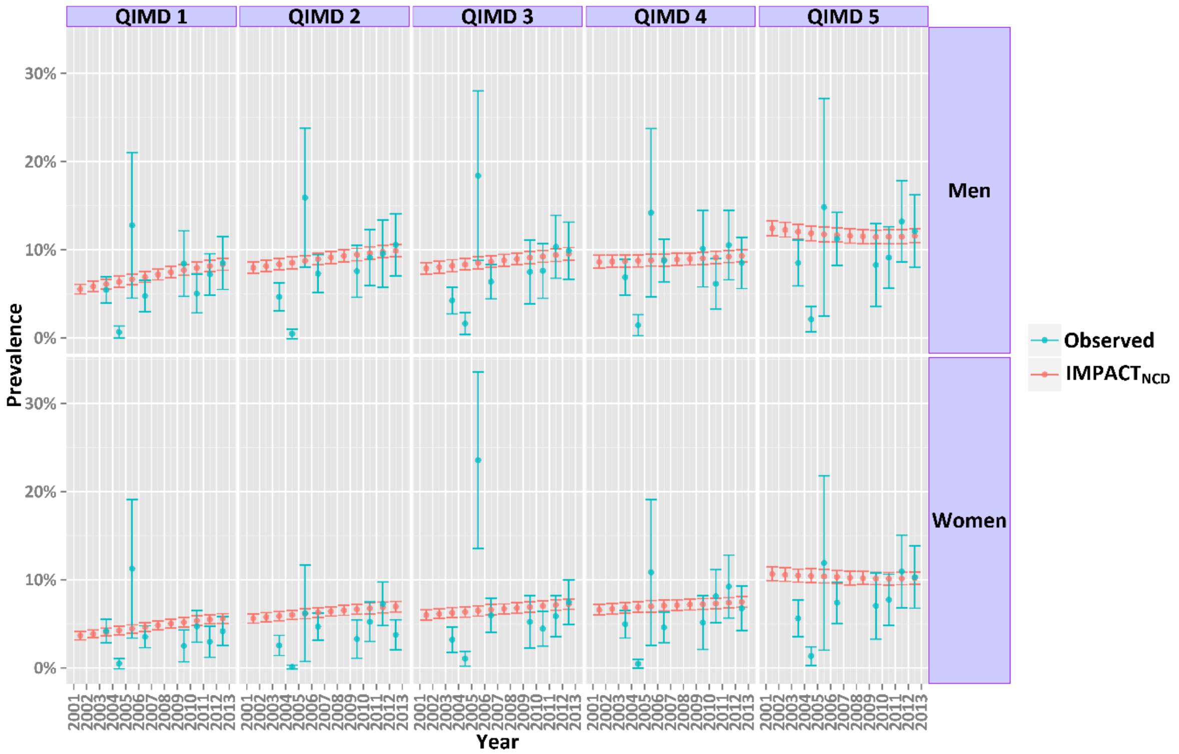

Figure S42 Diabetes mellitus prevalence for ages 30 – 84 by quintile group of index of multiple deprivation (QIMD, 1 = least deprived) between years 2001 and 2012. Observed in the population through Health Survey for England vs. IMPACT$_{NCD}$ synthetic population estimates. Error bars represent 95% confidence intervals of the mean.

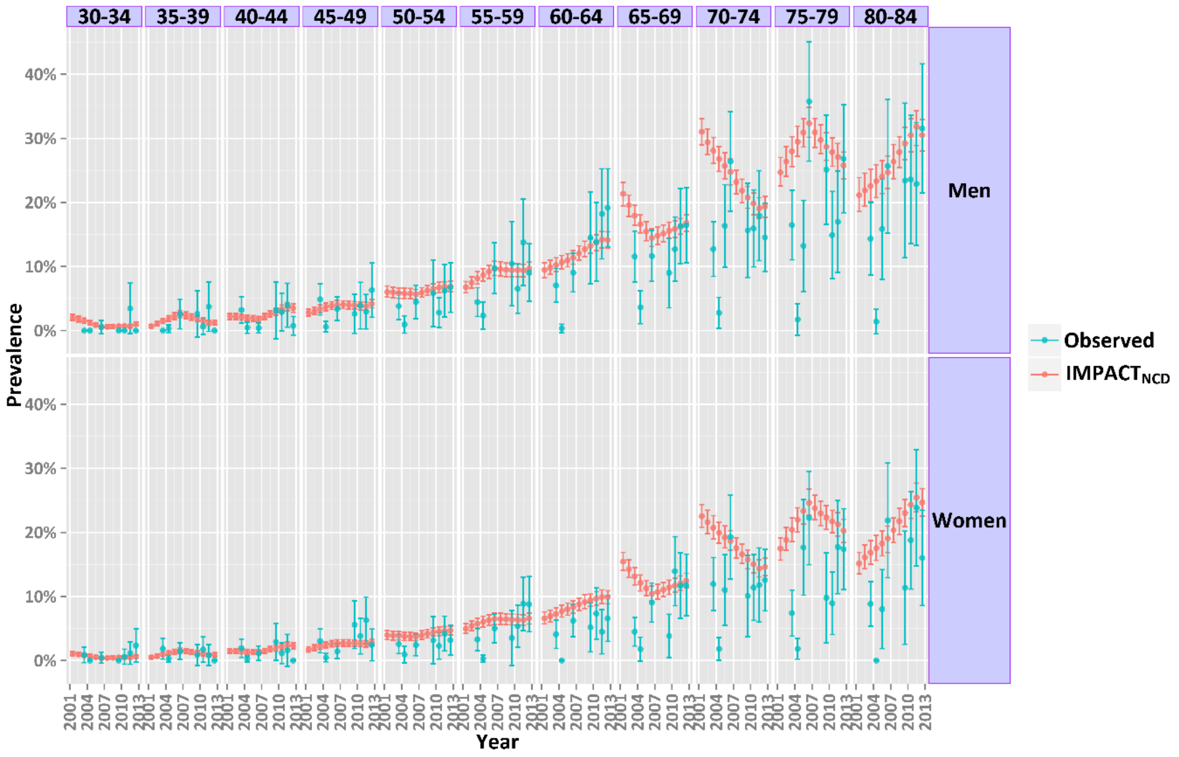

Figure S43 Diabetes mellitus prevalence for ages 30 – 84 by age group between years 2001 and 2012. Observed in the population through Health Survey for England vs. IMPACT$_{NCD}$ synthetic population estimates. Error bars represent 95% confidence intervals of the mean.

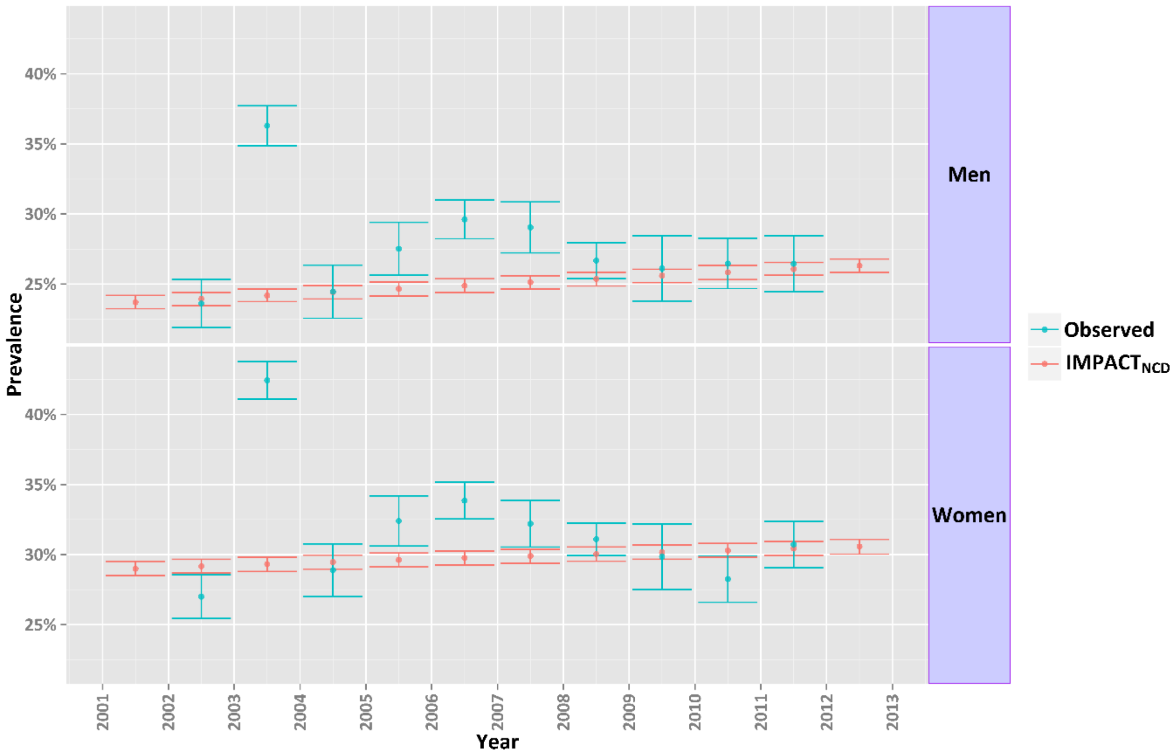

Figure S44 Five or more portions of fruit & veg per day prevalence for ages 30 – 84 between years 2001 and 2012. Observed in the population through Health Survey for England vs. IMPACT$_{NCD}$ synthetic population estimates. Error bars represent 95% confidence intervals of the mean.

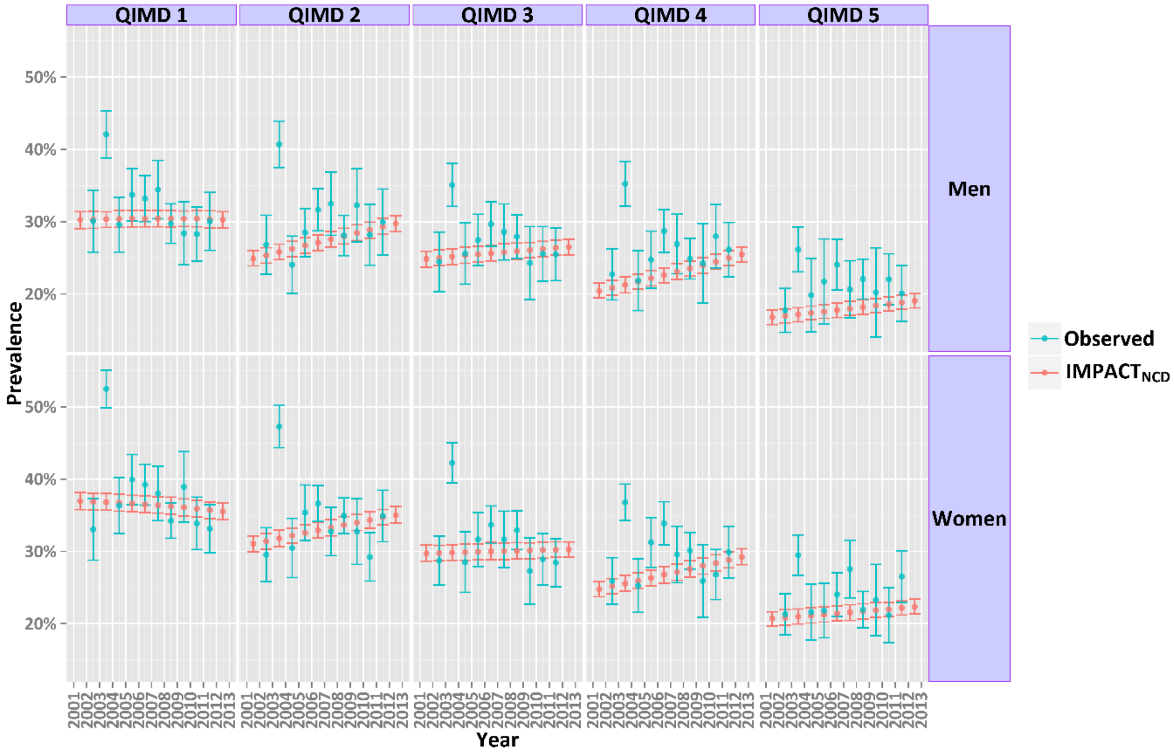

Figure S45 Five or more portions of fruit & veg per day prevalence for ages 30 – 84 by quintile group of index of multiple deprivation (QIMD, 1 = least deprived) between years 2001 and 2012. Observed in the population through Health Survey for England vs. IMPACT$_{NCD}$ synthetic population estimates. Error bars represent 95% confidence intervals of the mean.

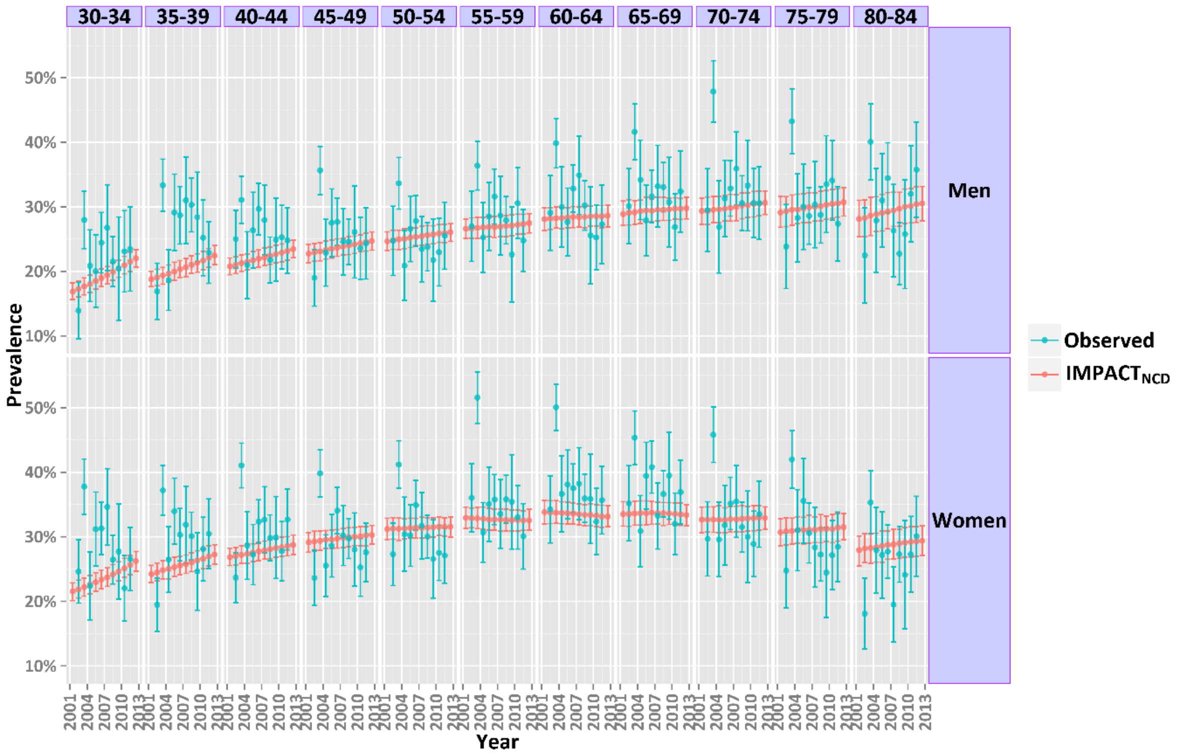

Figure S46 Five or more portions of fruit & veg per day prevalence for ages 30 – 84 by age group between years 2001 and 2012. Observed in the population through Health Survey for England vs. IMPACT$_{NCD}$ synthetic population estimates. Error bars represent 95% confidence intervals of the mean.

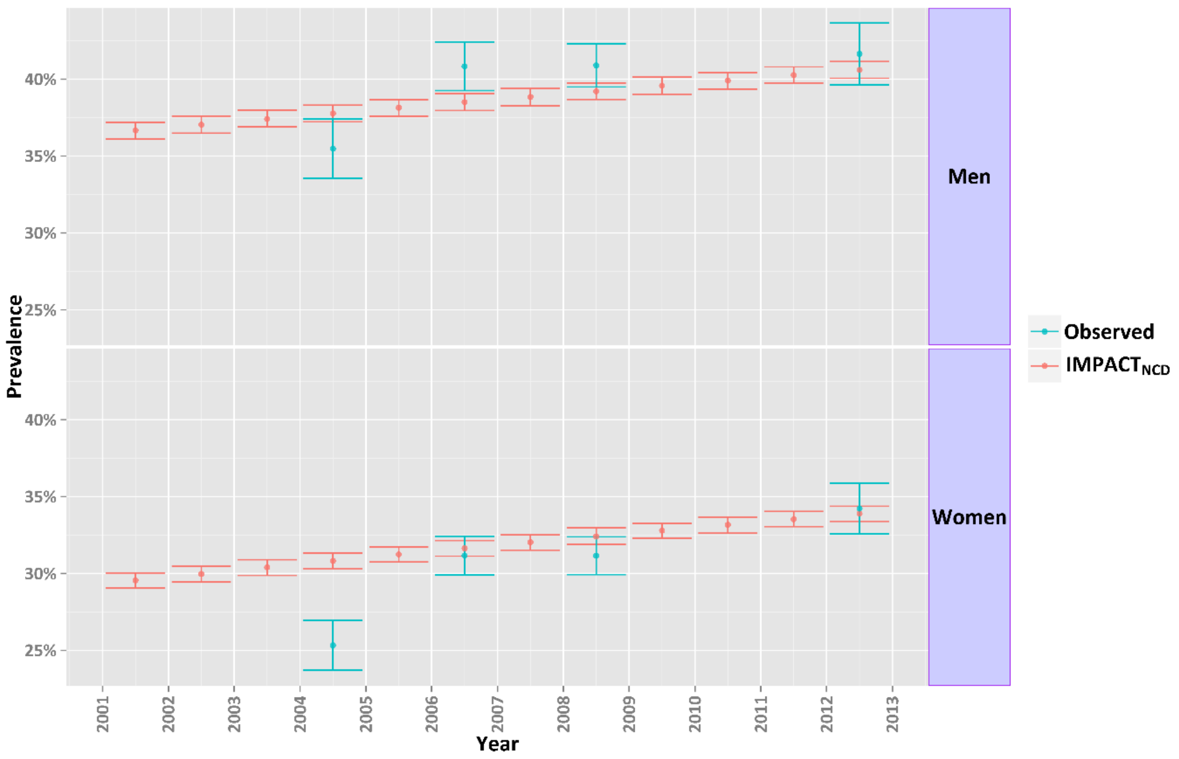

Figure S47 Five or more active days per week prevalence for ages 30 – 84 between years 2001 and 2012. Observed in the population through Health Survey for England vs. IMPACT$_{NCD}$ synthetic population estimates. Error bars represent 95% confidence intervals of the mean.

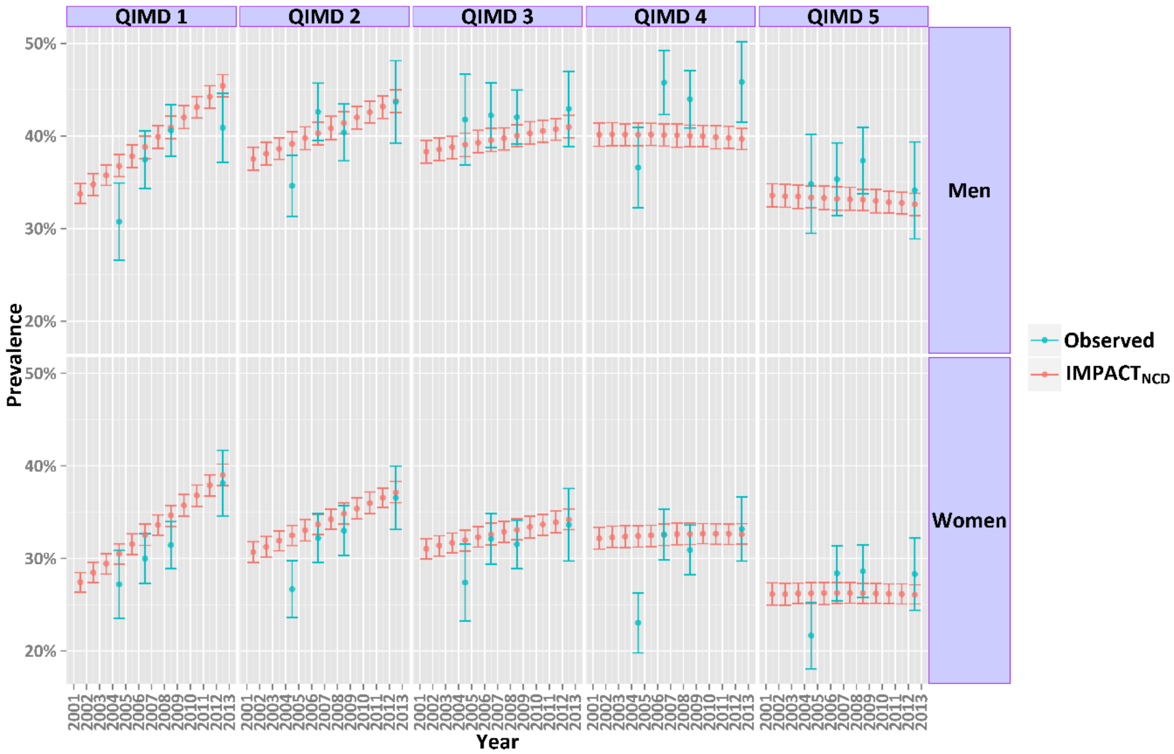

Figure S48 Five or more active days per week prevalence for ages 30 – 84 by quintile group of index of multiple deprivation (QIMD, 1 = least deprived) between years 2001 and 2012. Observed in the population through Health Survey for England vs. IMPACT$_{NCD}$ synthetic population estimates. Error bars represent 95% confidence intervals of the mean.

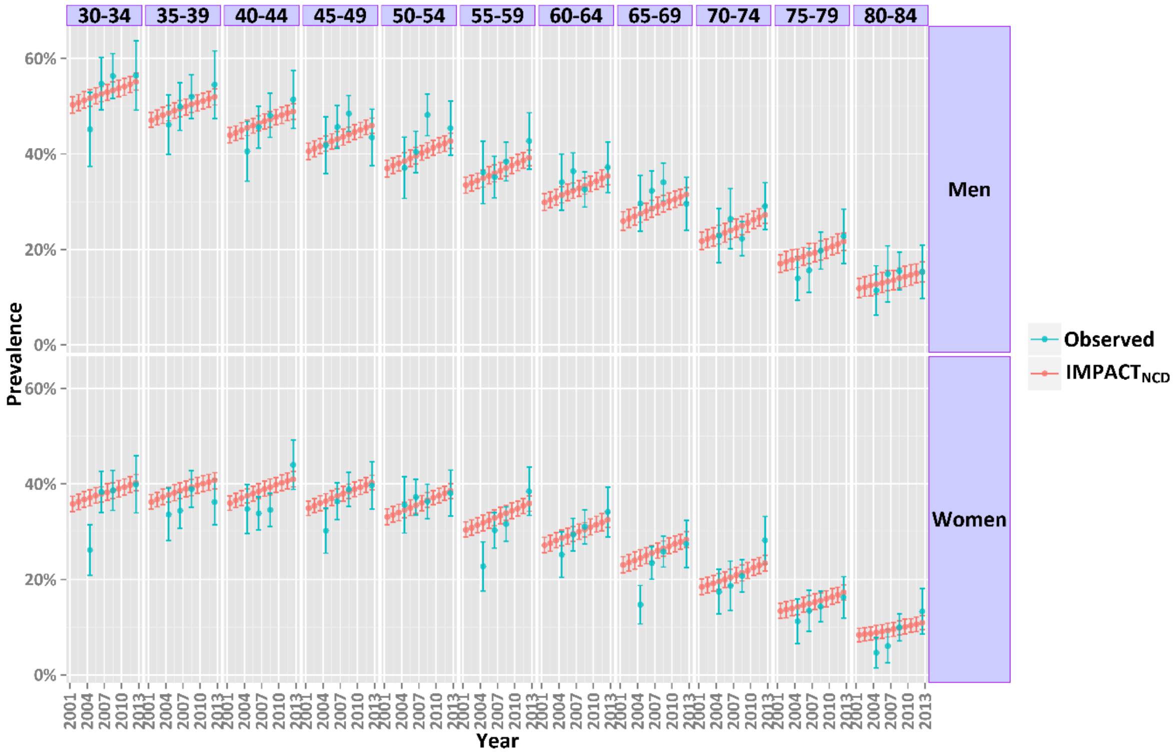

Figure S49 Five or more active days per week prevalence for ages 30 – 84 by age group between years 2001 and 2012. Observed in the population through Health Survey for England vs. IMPACT$_{NCD}$ synthetic population estimates. Error bars represent 95% confidence intervals of the mean.

Mortality concurrent and predictive validation

Here we validate the IMPACT$_{NCD}$ estimated mortality against the observed mortality in England between 2006 and 2013. Furthermore, we present the comparison of IMPACT$_{NCD}$ predicted CVD mortality rates, with forecasts from BAMP v1.3.0.1.[65] BAMP is a software that performs Bayesian-age-period-cohort forecasting and we used it to fit a model, with 2nd order random walks priors, to the observed CVD mortality in England for years 2002 to 2013 and then project it up to 2030. A more detailed description of the BAMP forecasting can be found elsewhere.[67]

Overall, the plots support the argument that IMPACT$_{NCD}$ is capable to translate changes in risk factors prevalence into changes in disease mortality, rather accurately.

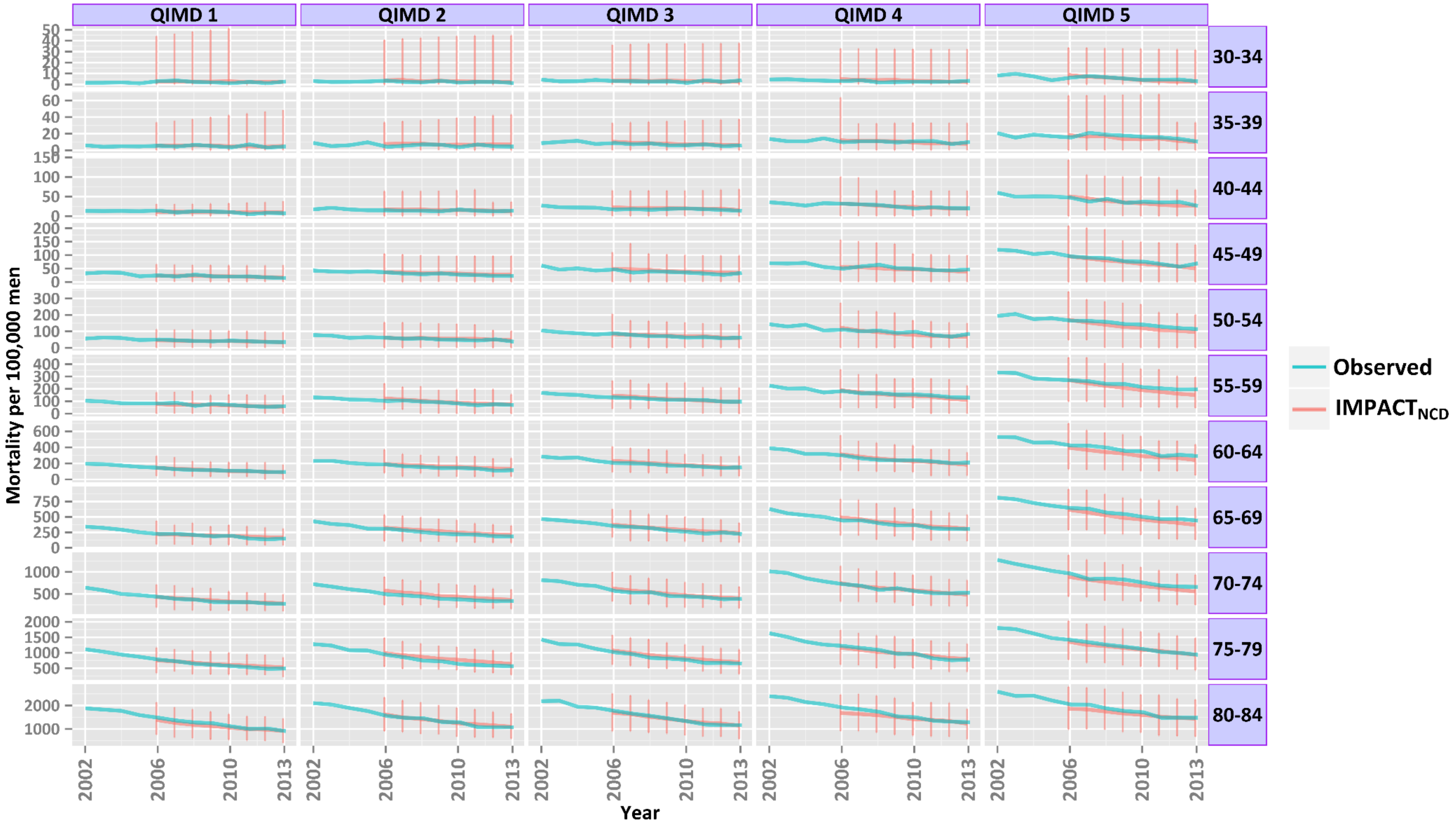

Figure S12 Coronary heart disease mortality (ICD10: I20 – I25) for men by age group and quintile group of index of multiple deprivation (QIMD, 1 = least deprived) between years 2002 and 2013. Observed in the population through mortality registries vs. $IMPACT_{NCD}$ synthetic population estimates. Error bars represent 95% uncertainty intervals.

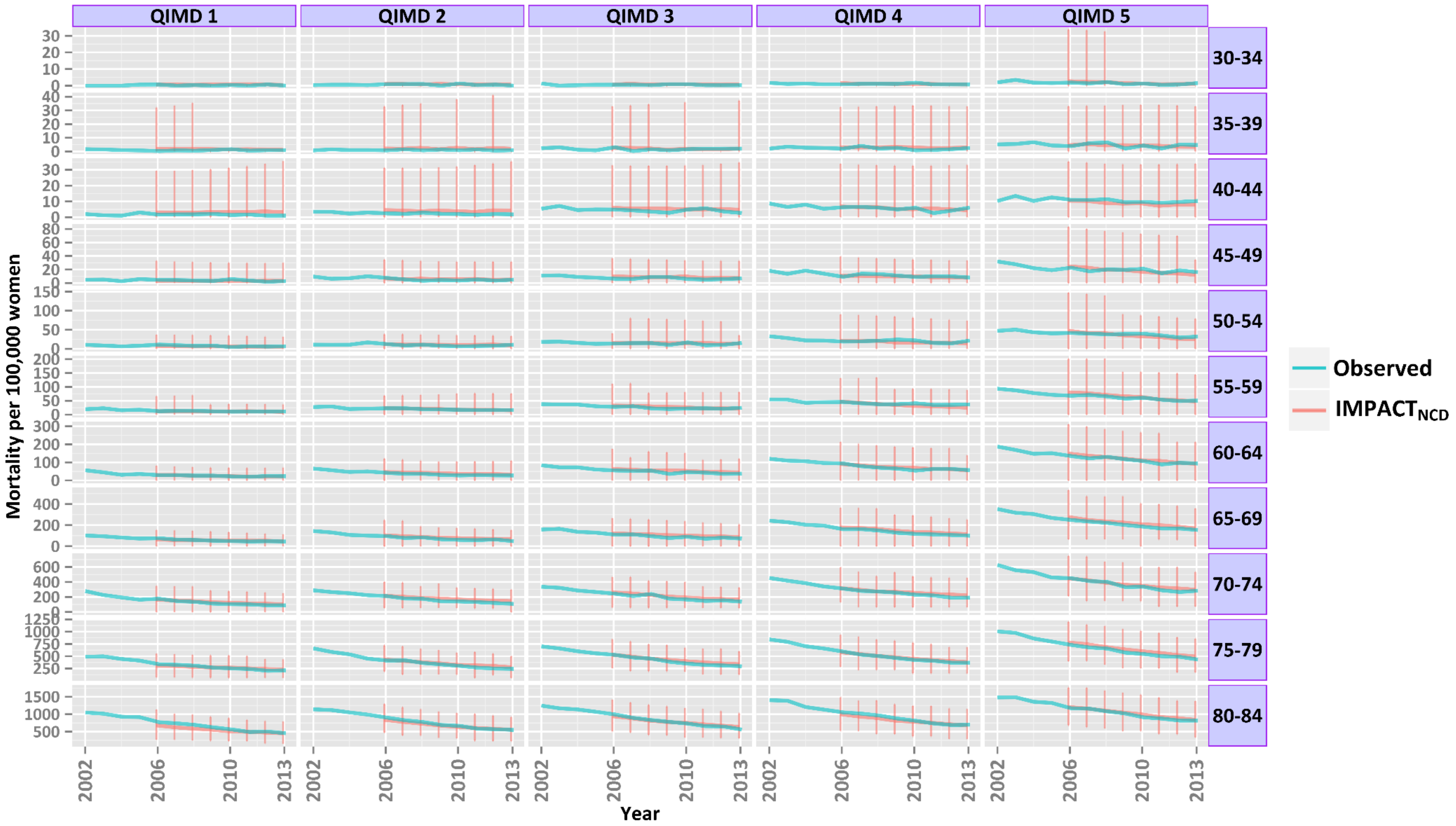

Figure S13 Coronary heart disease mortality (ICD10: I20 – I25) for women by age group and quintile group of index of multiple deprivation (QIMD, 1 = least deprived) between years 2002 and 2013. Observed in the population through mortality registries vs. IMPACT$_{NCD}$ synthetic population estimates. Error bars represent 95% uncertainty intervals.

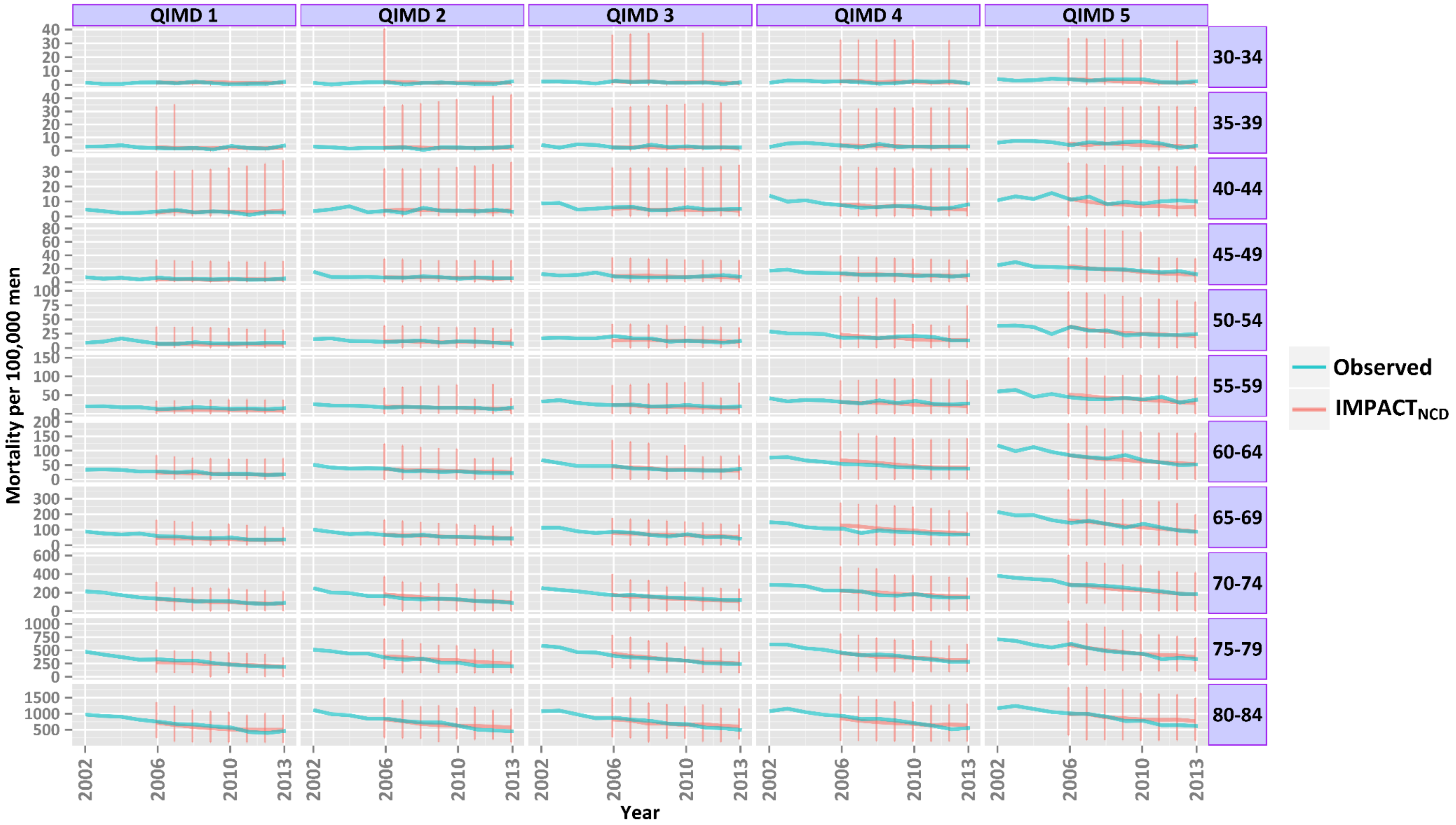

Figure S14 Stroke mortality (ICD10: I60 – I69) for men by age group and quintile group of index of multiple deprivation (QIMD, 1 = least deprived) between years 2002 and 2013. Observed in the population through mortality registries vs. IMPACT$_{NCD}$ synthetic population estimates. Error bars represent 95% uncertainty intervals.

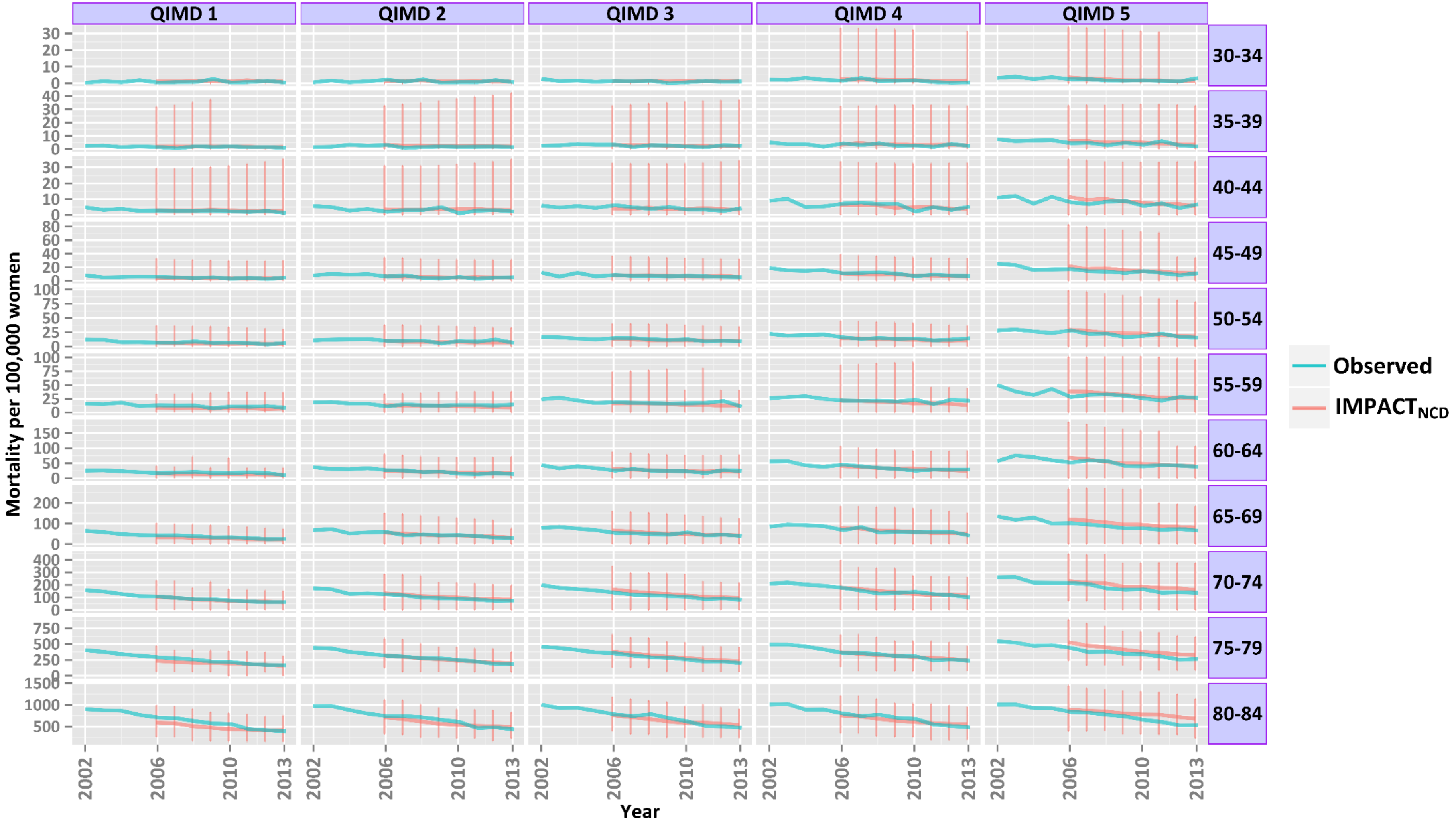

Figure S15 Stroke mortality (ICD10: I60 – I69) for women by age group and quintile group of index of multiple deprivation (QIMD, 1 = least deprived) between years 2002 and 2013. Observed in the population through mortality registries vs. IMPACT$_{NCD}$ synthetic population estimates. Error bars represent 95% uncertainty intervals.

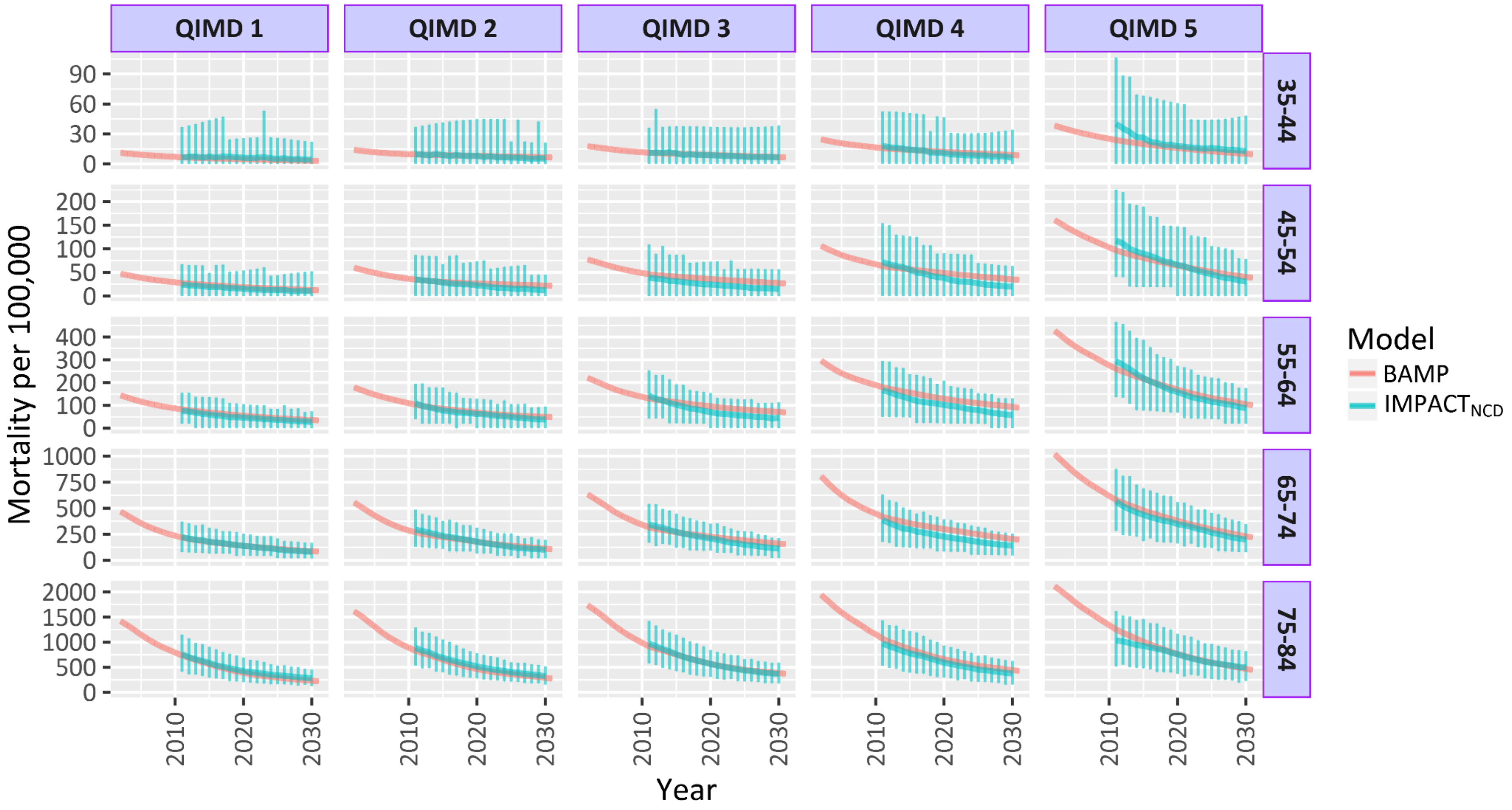

Figure S16 Coronary heart disease (ICD10: I20 – I25) mortality rates for men by age group and quintile group of index of multiple deprivation (QIMD, 1 = least deprived) between years 2002 and 2030. BAMP vs. IMPACT$_{NCD}$ estimates. Error bars represent 95% uncertainty intervals for IMPACT$_{NCD}$.

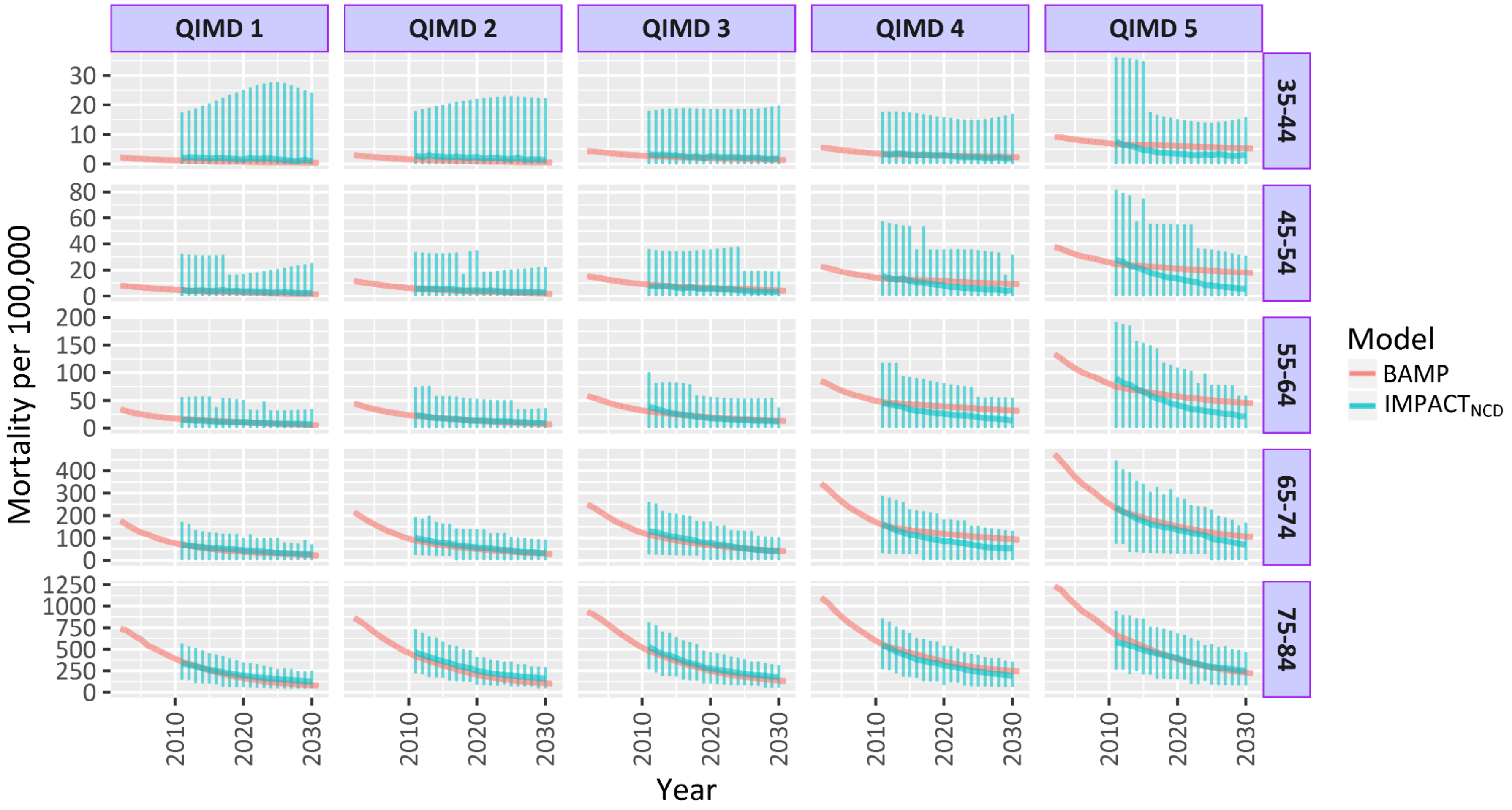

Figure S17 Coronary heart disease (ICD10: I20 – I25) mortality rates for women by age group and quintile group of index of multiple deprivation (QIMD, 1 = least deprived) between years 2002 and 2030. BAMP vs. IMPACT$_{NCD}$ estimates. Error bars represent 95% uncertainty intervals for IMPACT$_{NCD}$.

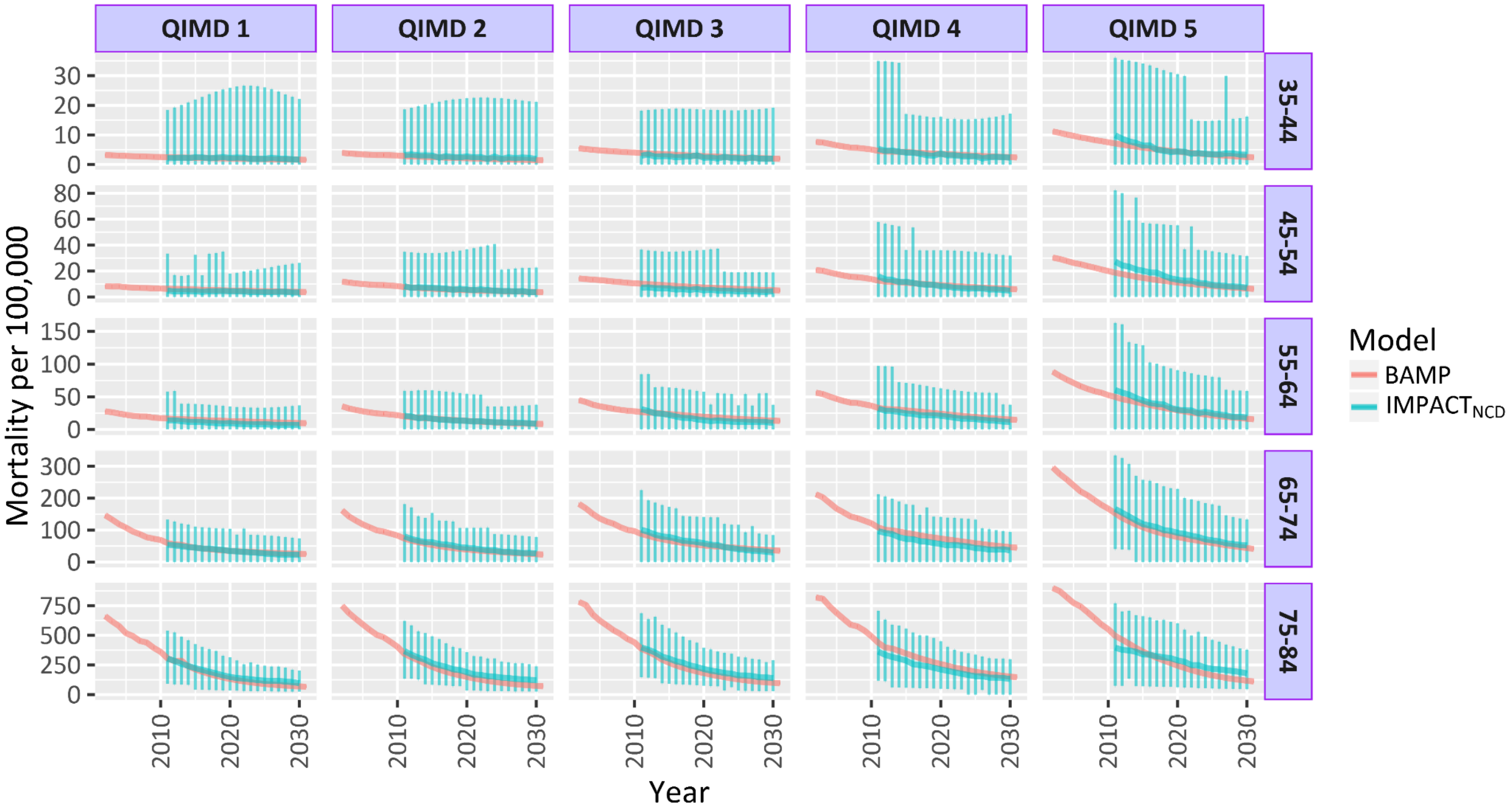

Figure S18 Stroke (ICD10: I60 – I69) mortality rates for men by age group and quintile group of index of multiple deprivation (QIMD, 1 = least deprived) between years 2002 and 2030. BAMP vs. IMPACT$_{NCD}$ estimates. Error bars represent 95% uncertainty intervals for IMPACT$_{NCD}$.

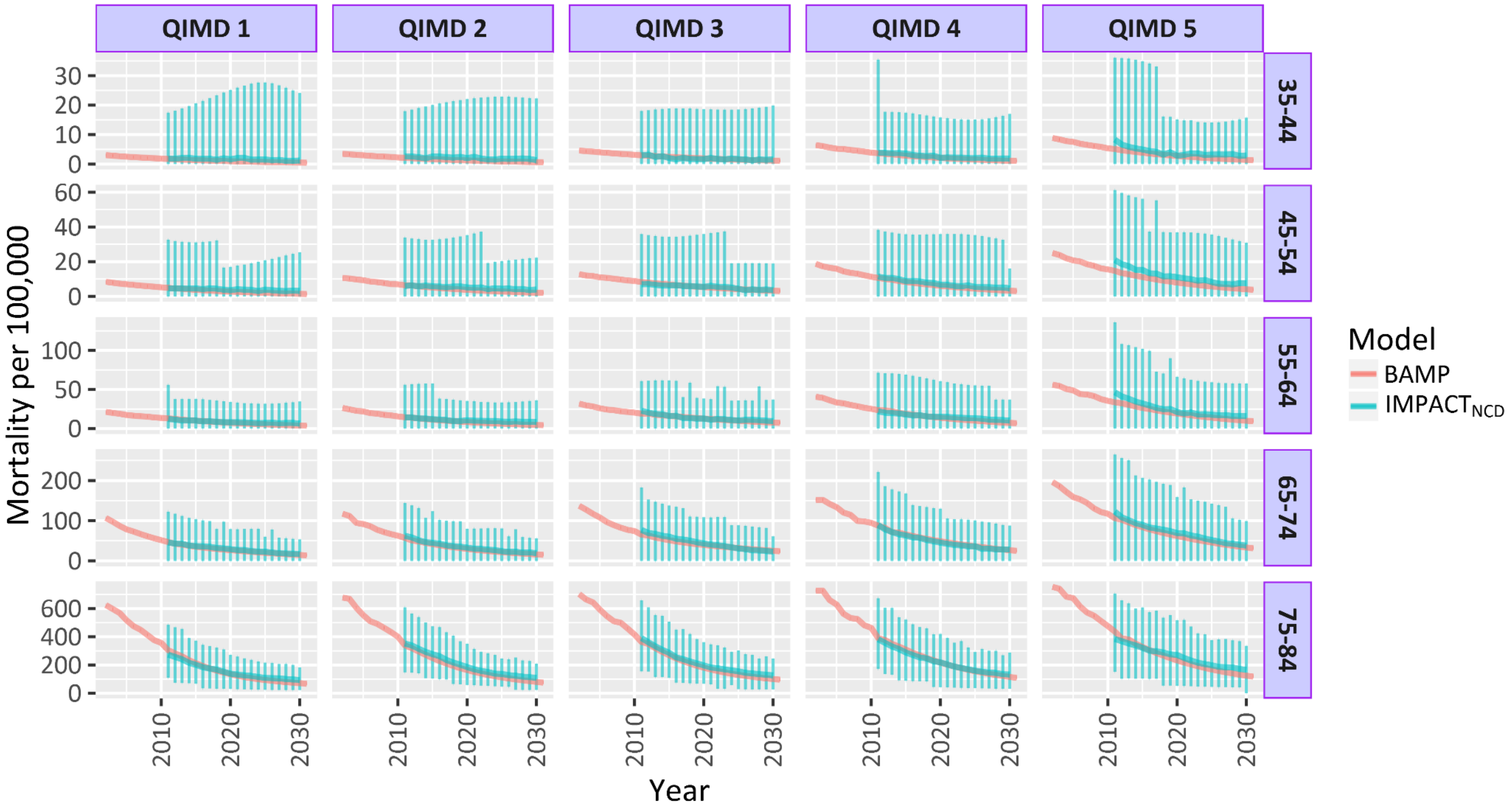

Figure S19 Stroke (ICD10: I60 – I69) mortality rates for women by age group and quintile group of index of multiple deprivation (QIMD, 1 = least deprived) between years 2002 and 2030. BAMP vs. $IMPACT_{NCD}$ estimates. Error bars represent 95% uncertainty intervals for $IMPACT_{NCD}$.

TABLES

Table S1 IMPACT$_{NCD}$ data sources.

Parameter	Outcome	Details	Comments	Source
Fertility rates	Births	Principal-assumption fertility projections for England	Stratified by age	National Population Projections, 2012-based Statistical Bulletin [Internet]. Office for National Statistics; 2013 [cited 2014 Nov 11]. Available from: http://www.ons.gov.uk/ons/rel/npp/national-population-projections/2012-based-projections/index.html
Mortality rates	Deaths from non-modelled causes	Mortality and mid-year population estimates for England	Stratified by age, sex, QIMD and cause of death. Years 2002-2013.	Data requested and obtained by the Office for National Statistics. Available from: http://www.ons.gov.uk/ons/about-ons/business-transparency/freedom-of-information/what-can-i-request/published-ad-hoc-data/health/december-2014/number-of-registered-deaths-by-sex--cause--year--the-adjusted-index.xls
Exposure to risk factors	Exposure of individuals	Health survey for England	Anonymised, individual-level datasets. Years 2001-2012.	Health survey for England 2001-2012. Data available to researchers from http://ukdataservice.ac.uk/
Relative risk for active smoking	CHD and stroke (ICD10: I20 – I25 and I60 – I69)	Re-analysis of American Cancer Society's Cancer Prevention Study II. Prospective cohort study, 6 years of follow up	Stratified by age and sex. Adjusted for age, race, education, marital status, "blue collar" employment in most recent or current job, weekly consumption of vegetables and citrus fruit, vitamin (A, C, and E) use, alcohol use, aspirin use, body mass index, exercise, dietary fat consumption, hypertension and diabetes at baseline.	Ezzati M, Henley SJ, Thun MJ, Lopez AD. Role of smoking in global and regional cardiovascular mortality. Circulation 2005;112:489–97. (Table 1 Model B)
	Other mortality (except CHD and stroke)	Male British doctors prospective cohort study	Age-standardised	Doll R, Peto R, Boreham J, Sutherland I. Mortality in relation to smoking: 50 years' observations on male British doctors. BMJ 2004;328:1519. (Table 1)

Parameter	Outcome	Details	Comments	Source
Relative risk for ex-smoking	CHD (ICD10: I20 – I25)	Meta- analysis. Multiple-adjusted pooled estimates from 19 prospective studies	Multiply-adjusted	Huxley RR, Woodward M. Cigarette smoking as a risk factor for coronary heart disease in women compared with men: a systematic review and meta-analysis of prospective cohort studies. The Lancet 2011;378:1297–305. (Web-figure 8)
	Stroke (ICD10 I60 – I69)	The Framingham study. Prospective cohort study	Stroke risk decreased significantly by two years and was at the level of non-smokers by five years after cessation of cigarette smoking. Hence, we considered no risk for ex-smokers.	Wolf PA, D'Agostino RB, Kannel WB, Bonita R, Belanger AJ. Cigarette smoking as a risk factor for stroke: The Framingham study. JAMA 1988;259:1025–9.
Relative risk for environmental tobacco smoking	CHD (ICD10: I20 – I25)	Meta-analysis of 10 cohort and case-control studies	Adjusted for important CHD risk factors.	He J, Vupputuri S, Allen K, Prerost MR, Hughes J, Whelton PK. Passive Smoking and the Risk of Coronary Heart Disease — A Meta-Analysis of Epidemiologic Studies. N Engl J Med 1999;340:920–6. (Table 3. Adjusted RR)
	Stroke (ICD10 I60 – I69)	Meta-analysis of 20 prospective, case-control and cross-sectional studies	13 studies adjusted for important CHD risk factors. The overall effect from all 20 studies was used.	Oono IP, Mackay DF, Pell JP. Meta-analysis of the association between second hand smoke exposure and stroke. J Public Health 2011;33:496–502. (Figure 1)
Relative risk for systolic blood pressure	CHD and stroke (ICD10: I20 – I25 and I60 – I69)	Meta-analysis of individual data from 61 prospective studies	Stratified by age and sex. Adjusted for regression dilution and total blood cholesterol and, where available, lipid fractions (HDL and non-HDL cholesterol), diabetes, weight, alcohol consumption, and smoking at baseline.	Age-specific relevance of usual blood pressure to vascular mortality: a meta-analysis of individual data for one million adults in 61 prospective studies. The Lancet 2002;360:1903–13. (Figures 3 and 5)
Relative risk for total cholesterol	CHD and stroke (ICD10: I20 – I25 and I60 – I69)	Meta-analysis of individual data from 61 prospective studies	Stratified by age and sex. Adjusted for regression dilution and age, sex, study, systolic blood pressure and smoking.	Prospective Studies Collaboration. Blood cholesterol and vascular mortality by age, sex, and blood pressure: a meta-analysis of individual data from 61 prospective studies with 55 000 vascular deaths. The Lancet 2007;370:1829–39. (Web-table 6 fully adjusted and Figure 3)

Parameter	Outcome	Details	Comments	Source
Relative risk for body mass index	CHD and stroke (ICD10: I20 – I25 and I60 – I69)	Meta-analysis of 58 prospective studies	Stratified by age. Adjusted for age, sex, smoking status, systolic blood pressure, history of diabetes, and total and HDL cholesterol. We used the age gradient from the adjusted only for age, sex, and smoking status reported estimates.	The Emerging Risk Factors Collaboration. Separate and combined associations of body-mass index and abdominal adiposity with cardiovascular disease: collaborative analysis of 58 prospective studies. The Lancet 2011;377:1085–95. (Table 1 and Figure 2)
Relative risk for diabetes mellitus	CHD and stroke (ICD10: I20 – I25 and I60 – I69)	Meta-analysis of 102 prospective studies	Stratified by age. Adjusted for age, smoking status, body-mass index, and systolic blood pressure.	The Emerging Risk Factors Collaboration. Diabetes mellitus, fasting blood glucose concentration, and risk of vascular disease: a collaborative meta-analysis of 102 prospective studies. The Lancet 2010;375:2215–22. (Figure 2)
	Other mortality (except CHD and stroke)	DECODE. A collaborative prospective study of 22 cohorts in Europe	Adjusted for BMI, blood pressure, smoking and serum cholesterol.	The DECODE Study Group. Is the current definition for diabetes relevant to mortality risk from all causes and cardiovascular and noncardiovascular diseases? Diabetes Care 2003;26:688–96.
Relative risk for physical activity	CHD and stroke (ICD10: I20 – I25 and I60 – I69)	Meta-analysis of 18 cohort studies for CHD and 8 cohort studies for ischaemic stroke	Stratified by age and sex. Adjusted for measurement error, age, sex, smoking, blood pressure and cholesterol.	Bull FC, Armstrong TP, Dixon T, Ham S, Neiman A, Pratt M. Comparative quantification of health risks. Chapter 10: physical inactivity. Geneva: World Health Organisation; 2004. (Tables 10.19 and 10.20)
Relative risk for fruit and vegetable consumption	CHD (ICD10: I20 – I25)	Meta-analysis of 9 cohort studies	RR per portion of F&V. Multiply-adjusted.	Dauchet L, Amouyel P, Hercberg S, Dallongeville J. Fruit and Vegetable Consumption and Risk of Coronary Heart Disease: A Meta-Analysis of Cohort Studies. J Nutr 2006;136:2588–93.
	Stroke (ICD10: I60 – I69)	Meta-analysis of 7 cohort studies	RR per portion of F&V. Multiply-adjusted.	Dauchet L, Amouyel P, Dallongeville J. Fruit and vegetable consumption and risk of stroke A meta-analysis of cohort studies. Neurology 2005;65:1193–7.

Table S2 IMPACT$_{NCD}$ main assumptions and limitations

Population module	Immigration is not considered.<br>Social mobility is not considered.<br>Quintile groups of index of multiple deprivation (QIMD) is a relative marker of (area) deprivation with several versions since 2003. We considered all version of QIMD identical.<br>We assume that the surveys used, are truly representative of the population. For example, the adjustments for selection bias in the Health Surveys for England are perfect.
Disease module	We assume multiplicative risk effects.<br>We assume log-linear dose-response for the continuous risk factors.<br>We assume that the effects of the risk factors on incidence and mortality are equal and risk factors are not modifying survival.<br>We assume all stroke types have common risk factors.<br>We assume 5-year lag time for CVD.<br>We assume 100% risk reversibility.<br>We assume that trends in disease incidence are attributable only to trends of the relevant modelled risk factors.<br>Only well accepted associations between upstream and downstream risk factors that have been observed in longitudinal studies are considered. However, the magnitudes of the associations are extracted from a series of nationally representative cross-sectional surveys (Health Survey for England).

Table S3 Coefficients of the model for fruit and vegetable consumption per day predictions. Brackets contain 95% confidence intervals for the coefficients

	**Dependent variable: Portions of fruit and veg per day**
Year	0.1*** (0.1, 0.1)
Age	-0.03*** (-0.05, -0.01)
Age^2	0.001 (-0.01, 0.01)
Sex = Woman	-0.1*** (-0.1, -0.1)
QIMD.L	0.03* (-0.01, 0.1)
QIMD.Q	-0.2*** (-0.2, -0.2)
QIMD.C	-0.2*** (-0.2, -0.1)
QIMD.4	-0.2*** (-0.2, -0.2)
Year : Age	-0.003 (-0.01, 0.01)
Year : Age^2	0.000 (-0.01, 0.01)
Year : Sex = Woman	-0.01 (-0.03, 0.02)
Year : QIMD.L	0.01 (-0.01, 0.03)
Year : QIMD.Q	-0.01 (-0.03, 0.01)
Year : QIMD.C	0.002 (-0.02, 0.03)
Year : QIMD.4	-0.02 (-0.04, 0.01)
Age : Age^2	-0.000 (-0.01, 0.01)
Age : Sex = Woman	0.02** (0.002, 0.04)
Age : QIMD.L	-0.01 (-0.03, 0.004)
Age : QIMD.Q	0.002 (-0.02, 0.02)
Age : QIMD.C	0.002 (-0.02, 0.02)
Age : QIMD.4	0.01 (-0.01, 0.02)
Age^2 : Sex = Woman	-0.000 (-0.02, 0.02)
Age^2 : QIMD.L	0.000 (-0.02, 0.02)
Age^2 : QIMD.Q	-0.000 (-0.02, 0.02)
Age^2 : QIMD.C	0.000 (-0.02, 0.02)
Age^2 : QIMD.4	-0.000 (-0.02, 0.02)
Sex = Woman : QIMD.L	-0.1*** (-0.1, -0.02)
Sex = Woman : QIMD.Q	0.02 (-0.02, 0.1)
Sex = Woman : QIMD.C	0.02 (-0.01, 0.1)
Sex = Woman : QIMD.4	-0.02 (-0.1, 0.02)
Notes:	*p<0.1; **p<0.05; ***p<0.01<br>: denotes interaction<br>QIMD was used as an ordered factor and its contrasts are based on orthogonal polynomials. L, C, Q, and 4 represent the linear, quadratic, cubic, and in the power of 4 effects, respectively.

Table S4 Coefficients of the model for active days per week predictions. Brackets contain 95% confidence intervals for the coefficients

	**Dependent variable: Active days per week**
Year	0.02^{**} (0.001, 0.04)
Age	-0.2^{***} (-0.3, -0.1)
Age^2	0.01 (-0.1, 0.1)
Age^3	-0.000 (-0.1, 0.1)
Sex = Woman	-1.9^{***} (-1.9, -1.8)
QIMD.L	0.2^{***} (0.1, 0.2)
QIMD.Q	0.1^{***} (0.03, 0.1)
QIMD.C	-0.01 (-0.1, 0.04)
QIMD.4	-0.2^{***} (-0.3, -0.2)
Year : QIMD.L	-0.05^{**} (-0.1, -0.001)
Year : QIMD.Q	0.01 (-0.03, 0.1)
Year : QIMD.C	0.001 (-0.04, 0.04)
Year : QIMD.4	0.001 (-0.04, 0.04)
Age : Age^3	0.000 (-0.02, 0.02)
Age : Sex = Woman	0.1^{***} (0.02, 0.1)
Age : QIMD.L	-0.02 (-0.1, 0.1)
Age : QIMD.Q	-0.01 (-0.1, 0.1)
Age : QIMD.C	-0.002 (-0.1, 0.1)
Age : QIMD.4	0.004 (-0.1, 0.1)
Age^2 : Age^3	-0.0 (-0.02, 0.02)
Age^2 : Sex = Woman	-0.001 (-0.04, 0.04)
Age^3 : QIMD.L	0.000 (-0.05, 0.05)
Age^3 : QIMD.Q	0.000 (-0.05, 0.05)
Age^3 : QIMD.C	0.000 (-0.05, 0.05)
Age^3 : QIMD.4	0.000 (-0.04, 0.04)
Notes:	$^{*}p<0.1$; $^{**}p<0.05$; $^{***}p<0.01$<br>: denotes interaction<br>QIMD was used as an ordered factor and its contrasts are based on orthogonal polynomials. L, C, Q, and 4 represent the linear, quadratic, cubic, and in the power of 4 effects, respectively.

Table S5 Coefficients of the model for smoking initiation predictions. Brackets contain 95% confidence intervals for the coefficients

	**Dependent variable: Smoking initiation**
Year	-0.02 (-0.1, 0.05)
Sex = Woman	-0.3*** (-0.5, -0.1)
Age	-0.1*** (-0.1, -0.1)
QIMD > 1	0.3*** (0.1, 0.5)
Constant	-1.0*** (-1.4, -0.5)
Notes:	*p<0.1; **p<0.05; ***p<0.01<br>QIMD was used as binary variable with levels: 1 (least deprived quintile) and 2 (all other more deprived quintiles)

Table S6 Coefficients of the model for smoking cessation predictions. Brackets contain 95% confidence intervals for the coefficients

	**Dependent variable: Smoking cessation**
Year	0.03*** (0.02, 0.05)
Age	2.8*** (2.2, 3.5)
Sex = Woman	1.0*** (0.3, 1.7)
QIMD.L	-0.4*** (-0.6, -0.3)
QIMD.Q	-0.2** (-0.4, -0.1)
QIMD.C	-0.1 (-0.3, 0.1)
QIMD.4	-0.1 (-0.3, 0.1)
Age2	-0.1*** (-0.2, -0.1)
Age3	0.002*** (0.002, 0.003)
Age4	-0.000*** (-0.000, -0.000)
Year : QIMD.L	-0.02 (-0.05, 0.02)
Year : QIMD.Q	-0.03** (-0.1, -0.003)
Year : QIMD.C	-0.02 (-0.1, 0.01)
Year : QIMD.4	-0.01 (-0.04, 0.02)
Age : Sex = Woman	-0.04** (-0.1, -0.005)
Age2 : Age3	0.000*** (0.000, 0.000)
Sex = Woman : Age2	0.000* (-0.000, 0.001)
Constant	-26.8*** (-32.0, -21.6)
Notes:	*p<0.1; **p<0.05; ***p<0.01<br>: denotes interaction<br>QIMD was used as an ordered factor and its contrasts are based on orthogonal polynomials. L, C, Q, and 4 represent the linear, quadratic, cubic, and in the power of 4 effects, respectively.

Table S7 Coefficients of the model for smoking relapse predictions. Brackets contain 95% confidence intervals for the coefficients

	**Dependent variable: Smoking relapse**
(years since cessation + 1)$^{-2}$	4.3*** (3.5, 5.1)
Sex = Woman	0.04 (-0.2, 0.3)
QIMD.L	-0.5*** (-0.8, -0.2)
QIMD.Q	0.02 (-0.3, 0.3)
QIMD.C	0.002 (-0.3, 0.3)
QIMD.4	0.3* (0.01, 0.5)
Constant	-0.4*** (-0.6, -0.2)
Notes:	*p<0.1; **p<0.05; ***p<0.01<br>: denotes interaction<br>QIMD was used as an ordered factor and its contrasts are based on orthogonal polynomials. L, C, Q, and 4 represent the linear, quadratic, cubic, and in the power of 4 effects, respectively

Table S8 Coefficients of the model for body mass index predictions. Brackets contain 95% confidence intervals for the coefficients

	**Dependent variable: Body mass index (kg/m^2)**
Year	0.01 (-0.04, 0.1)
Age	0.3*** (0.3, 0.3)
Sex = Woman	-0.8*** (-1.3, -0.2)
QIMD.L	0.3*** (0.1, 0.6)
QIMD.Q	-0.1 (-0.3, 0.2)
QIMD.C	0.2 (-0.04, 0.4)
QIMD.4	0.1 (-0.1, 0.3)
Active days per week	-0.1*** (-0.1, -0.1)
Age^2	-0.003*** (-0.003, -0.002)
Year : Age	0.001*** (0.000, 0.002)
Year : QIMD.L	0.04** (0.01, 0.1)
Year : QIMD.Q	0.02 (-0.02, 0.1)
Year : QIMD.C	0.02 (-0.02, 0.1)
Year : QIMD.4	0.01 (-0.02, 0.04)
Age : Sex = Woman	0.01** (0.002, 0.02)
Sex = Woman : QIMD.L	1.1*** (0.8, 1.4)
Sex = Woman : QIMD.Q	0.1 (-0.1, 0.4)
Sex = Woman : QIMD.C	-0.1 (-0.4, 0.2)
Sex = Woman : QIMD.4	-0.02 (-0.3, 0.3)
Sex = Woman : Active days per week	-0.1*** (-0.1, -0.03)
Constant	20.4*** (19.6, 21.1)
Notes:	*p<0.1; **p<0.05; ***p<0.01<br>: denotes interaction<br>QIMD was used as an ordered factor and its contrasts are based on orthogonal polynomials. L, C, Q, and 4 represent the linear, quadratic, cubic, and in the power of 4 effects, respectively

Table S9 Coefficients of the model for systolic blood pressure predictions. Brackets contain 95% confidence intervals for the coefficients

	**Dependent variable: Systolic blood pressure (mmHg)**
Year	-0.1^{**} (-0.2, -0.03)
Age	23.3^{***} (8.7, 37.9)
Age^2	-0.5^{***} (-0.6, -0.3)
Age^3	0.01^{***} (0.002, 0.01)
Sex = Woman	134.1^{***} (47.0, 221.3)
QIMD.L	-3.5^{***} (-5.6, -1.4)
QIMD.Q	0.6 (-1.5, 2.7)
QIMD.C	1.4 (-0.7, 3.5)
QIMD.4	-0.02 (-2.1, 2.1)
ln(BMI)	-534.9^{***} (-939.2, -130.5)
$(\ln(BMI))^2$	219.8^{***} (99.8, 339.7)
Active smoker = Yes	11.8^{***} (3.7, 19.8)
Active days per week	0.1^{***} (0.05, 0.2)
Age : Sex = Woman	-0.6^{***} (-1.1, -0.2)
Age : QIMD.L	0.1^{***} (0.04, 0.2)
Age : QIMD.Q	-0.04 (-0.1, 0.03)
Age : QIMD.C	-0.04 (-0.1, 0.03)
Age : QIMD.4	-0.01 (-0.1, 0.1)
Age : ln(BMI)	-13.1^{***} (-21.8, -4.3)
Age : $(\ln(BMI))^2$	2.2^{***} (0.9, 3.5)
Age^2 : Sex = Woman	0.02^{***} (0.01, 0.03)
Age^2 : ln(BMI)	0.2^{***} (0.1, 0.2)
Age^2 : $(\ln(BMI))^2$	-0.03^{***} (-0.04, -0.01)
Age^2 : Active smoker = Yes	0.001^{***} (0.000, 0.001)
Age^3 : Age^2	0.000^{***} (0.000, 0.000)
Age^3 : Year	-0.000^{***} (-0.000, -0.000)
Age^3 : Age	-0.000^{***} (-0.000, -0.000)
Age^3 : Sex = Woman	-0.000^{***} (-0.000, -0.000)
Age^3 : QIMD.L	-0.000^{**} (-0.000, -0.000)
Age^3 : QIMD.Q	0.000^{*} (-0.000, 0.000)
Age^3 : QIMD.C	0.000 (-0.000, 0.000)
Age^3 : QIMD.4	0.000 (-0.000, 0.000)
Sex = Woman : ln(BMI)	-83.6^{***} (-136.6, -30.7)
Sex = Woman : $(\ln(BMI))^2$	12.3^{***} (4.3, 20.3)
Sex = Woman : Active smoker = Yes	0.7 (-0.2, 1.6)
ln(BMI) : $(\ln(BMI))^2$	-28.3^{***} (-40.8, -15.7)
ln(BMI) : Active smoker = Yes	-3.7^{***} (-6.2, -1.2)
Constant	487.6^{**} (10.6, 964.7)
Note:	$^{*}p<0.1$; $^{**}p<0.05$; $^{***}p<0.01$<br>: denotes interaction<br>QIMD was used as an ordered factor and its contrasts are based on orthogonal polynomials. L, C, Q, and 4 represent the linear, quadratic, cubic, and in the power of 4 effects, respectively

Table S10 Coefficients of the model for total cholesterol predictions. Brackets contain 95% confidence intervals for the coefficients

	Dependent variable: Total cholesterol mmol/l
Year	-0.01 (-0.03, 0.01)
Age	0.1*** (0.1, 0.1)
Sex = Woman	-0.5*** (-0.7, -0.3)
QIMD.L	0.2*** (0.1, 0.3)
QIMD.Q	-0.01 (-0.1, 0.1)
QIMD.C	0.02 (-0.1, 0.1)
QIMD.4	0.1 (-0.02, 0.2)
Active days per week	-0.02*** (-0.04, -0.01)
BMI	0.02** (0.01, 0.04)
F&V portions per day	-0.02*** (-0.02, -0.01)
I(Age2)	-0.001*** (-0.001, -0.001)
BMI^{-1}	-47.5*** (-57.8, -37.1)
Year : Age	-0.001** (-0.001, -0.000)
Age : Sex = Woman	0.02*** (0.02, 0.02)
Age : QIMD.L	-0.01*** (-0.01, -0.004)
Age : QIMD.Q	-0.001 (-0.003, 0.001)
Age : QIMD.C	-0.001 (-0.003, 0.001)
Age : QIMD.4	-0.002* (-0.004, 0.000)
Age : Active days per week	0.001*** (0.000, 0.001)
Age : BMI	-0.001*** (-0.001, -0.001)
Sex = Woman : BMI	-0.02*** (-0.02, -0.01)
Constant	4.7*** (3.9, 5.6)
Note :	*p<0.1; **p<0.05; ***p<0.01<br>: denotes interaction<br>QIMD was used as an ordered factor and its contrasts are based on orthogonal polynomials. L, C, Q, and 4 represent the linear, quadratic, cubic, and in the power of 4 effects, respectively

Table S11 Estimated cases and deaths prevented or postponed under each scenario, by 2030. Brackets contain the respective interquartile ranges (IQR).

Scenarios	Cases prevented or postponed by 2030 (IQR)	Deaths prevented or postponed by 2030 (IQR)
20% treatment threshold universal screening	7,000 (-2,000 to 15,000)	2,300 (-1,200 to 5,600)
Socioeconomic differential uptake universal screening	19,000 (10,000 to 27,000)	700 (-2,400 to 4,000)
Diet-only population-wide intervention	47,000 (37,000 to 56,000)	5,600 (2,700 to 8,700)

Table S12 Cases prevented or postponed per quintile groups of the index of multiple deprivation (QIMD), by 2030. The absolute equity slope index for each scenario is also presented. Brackets contain the respective interquartile ranges (IQR).

QIMD	20% treatment threshold universal screening	Socioeconomic differential uptake universal screening	Diet-only population-wide intervention
1 (least deprived)	1,400 (-3,600 to 6,200)	3,200 (-1,800 to 7,800)	8,600 (4,100 to 13,400)
2	700 (-4,800 to 5,500)	3,900 (-900 to 9,000)	9,100 (4,000 to 13,900)
3	1,100 (-4,100 to 6,700)	4,400 (-1,500 to 9,400)	9,400 (4,700 to 14,600)
4	1,100 (-3,500 to 6,400)	3,400 (-1,300 to 8,900)	9,100 (4,200 to 13,700)
5 (most deprived)	2,900 (-2,800 to 8,400)	4,300 (-1,300 to 9,600)	10,400 (5,500 to 15,800)
Absolute equity slope index	2,000 (-6,700 to 10,600)	300 (-6900 to 9,000)	2,200 (-5,300 to 9,900)

Table S13 Relative percentage reduction in cardiovascular disease cases per quintile groups of the index of multiple deprivation (QIMD), by 2030. The relative equity slope index for each scenario is also presented. Brackets contain the respective interquartile ranges (IQR).

QIMD	20% treatment threshold universal screening	Socioeconomic differential uptake universal screening	Diet-only population-wide intervention
1 (least deprived)	0.6% (-1.4 to 2.4%)	1.2% (-0.7 to 3.0%)	3.3% (1.6 to 5.1%)
2	0.2% (-1.6 to 1.9%)	1.4% (-0.4 to 3.1%)	3.1% (1.4 to 4.8%)
3	0.4% (-1.4 to 2.3%)	1.5% (-0.5 to 3.2%)	3.3% (1.6 to 5.0%)
4	0.4% (-1.3 to 2.3%)	1.2% (-0.5 to 3.2%)	3.3% (1.5 to 5.0%)
5 (most deprived)	1.0% (-0.9 to 2.7%)	1.4% (-0.4 to 3.1%)	3.4% (1.8 to 5.3%)
Relative equity slope index	0.6 (-2.4 to 3.8)	0.0 (-2.6 to 3.0)	0.4 (-2.2 to 3.0)

Table S14 Distributions that were used as inputs for the simulations. Numbers are rounded

Variable	Sex	Ages	Distribution
**Relative risks of relevant risk factors for CHD**			
Active smoking[68 table 1 model B]	Men	30 - 44	Log-Normal (mean = ln(5.51), sd = ln(12.3 / 5.51) / 1.96)
		45 - 59	Log-Normal (mean = ln(3.04), sd = ln(3.48 / 3.04) / 1.96)
		60 - 69	Log-Normal (mean = ln(1.88), sd = ln(2.08 / 1.88) / 1.96)
		70 - 79	Log-Normal (mean = ln(1.44), sd = ln(1.63 / 1.44) / 1.96)
	Women	30 - 44	Log-Normal (mean = ln(2.26), sd = ln(6.14 / 2.26) / 1.96)
		45 - 59	Log-Normal (mean = ln(3.78), sd = ln(4.62 / 3.78) / 1.96)
		60 - 69	Log-Normal (mean = ln(2.53), sd = ln(2.87 / 2.53) / 1.96)
		70 - 79	Log-Normal (mean = ln(1.68), sd = ln(1.93 / 1.68) / 1.96)
		80 - 84	Log-Normal (mean = ln(1.38), sd = ln(1.77 / 1.38) / 1.96)
Ex-Smoking[69 web-figure 8]	Men	30 - 84	Log-Normal (mean = ln(1.25), sd = ln(1.32 / 1.25) / 1.96)
	Women	30 - 84	Log-Normal (mean = ln(1.2), sd = ln(1.34 / 1.2) / 1.96)
ETS[70 table 3 adjusted RR]	Both	30 - 84	Log-Normal (mean = ln(1.26), sd = ln(1.38 / 1.26) / 1.96)
SBP[71 figure 5]	Men	30 - 49	Log-Normal (mean = ln(0.5), sd = ln(0.54 / 0.5) / 1.96)
		50 - 59	Log-Normal (mean = ln(0.5), sd = ln(0.52 / 0.5) / 1.96)
		60 - 69	Log-Normal (mean = ln(0.55), sd = ln(0.57 / 0.55) / 1.96)

Variable	Sex	Ages	Distribution
		70 - 74	Log-Normal (mean = ln(0.62), sd = ln(0.64 / 0.62) / 1.96)
		80 - 84	Log-Normal (mean = ln(0.69), sd = ln(0.73 / 0.69) / 1.96)
	Women	30 - 49	Log-Normal (mean = ln(0.4), sd = ln(0.49 / 0.4) / 1.96)
		50 - 59	Log-Normal (mean = ln(0.49), sd = ln(0.54 / 0.49) / 1.96)
		60 - 69	Log-Normal (mean = ln(0.5), sd = ln(0.61 / 0.5) / 1.96)
		70 - 74	Log-Normal (mean = ln(0.55), sd = ln(0.58 / 0.55) / 1.96)
		80 - 84	Log-Normal (mean = ln(0.64), sd = ln(0.68 / 0.64) / 1.96)
TC[72 web-table 6]	Both	30 - 49	Log-Normal (mean = ln(0.49), sd = ln(0.52 / 0.49) / 1.96)
		50 - 59	Log-Normal (mean = ln(0.62), sd = ln(0.65 / 0.62) / 1.96)
		60 - 69	Log-Normal (mean = ln(0.74), sd = ln(0.76 / 0.74) / 1.96)
		70 - 74	Log-Normal (mean = ln(0.84), sd = ln(0.86 / 0.84) / 1.96)
		80 - 84	Log-Normal (mean = ln(0.87), sd = ln(0.9 / 0.87) / 1.96)
BMI[73 table 1 and figure 2]	Both	30 - 59	Log-Normal (mean = ln(1.21), sd = ln(1.28 / 1.21) / 1.96)
		60 - 69	Log-Normal (mean = ln(1.06), sd = ln(1.12 / 1.06) / 1.96)
Diabetes[74 figure 2]	Both	40 - 59	Log-Normal (mean = ln(2.51), sd = ln(2.8/ 2.51) / 1.96)
		60 - 69	Log-Normal (mean = ln(2.01), sd = ln(2.26/ 2.01) / 1.96)
		70 - 84	Log-Normal (mean = ln(1.78), sd = ln(2.05/ 1.78) / 1.96)

Variable	Sex	Ages	Distribution
PA[75 table 10.19]	Both	30 - 69	No active days: Log-Normal (mean = ln(1.71), sd = ln(1.85/ 1.71) / 1.96)
			1 – 4 active days: Log-Normal (mean = ln(1.44), sd = ln(1.62/ 1.44) / 1.96)
		70 - 79	No active days: Log-Normal (mean = ln(1.5), sd = ln(1.61/ 1.5) / 1.96)
			1 – 4 active days: Log-Normal (mean = ln(1.31), sd = ln(1.48/ 1.31) / 1.96)
		80 - 84	No active days: Log-Normal (mean = ln(1.4), sd = ln(1.41/ 1.4) / 1.96)
			1 – 4 active days: Log-Normal (mean = ln(1.2), sd = ln(1.35/ 1.2) / 1.96)
F&V[76]			Log-Normal (mean = ln(0.96), sd = ln(1.0.99/ 0.96) / 1.96)
**Relative risks of relevant risk factors for stroke**			
Active smoking[68 table 1 model B]	Men	30 - 59	Log-Normal (mean = ln(3.12), sd = ln(4.64 / 3.12) / 1.96)
		60 - 69	Log-Normal (mean = ln(1.87), sd = ln(2.44 / 1.87) / 1.96)
		70 - 79	Log-Normal (mean = ln(1.39), sd = ln(1.77 / 1.39) / 1.96)
	Women	30 - 59	Log-Normal (mean = ln(4.61), sd = ln(6.37 / 4.61) / 1.96)
		60 - 69	Log-Normal (mean = ln(2.81), sd = ln(3.58 / 2.81) / 1.96)
		70 - 79	Log-Normal (mean = ln(1.95), sd = ln(2.45 / 1.95) / 1.96)
ETS[77 figure 1]	Both	30 - 84	Log-Normal (mean = ln(1.25), sd = ln(1.38 / 1.25) / 1.96)
SBP[71 figure 3]	Men	30 - 49	Log-Normal (mean = ln(0.33), sd = ln(0.38 / 0.33) / 1.96)
		50 - 59	Log-Normal (mean = ln(0.34), sd = ln(0.37 / 0.34) / 1.96)

Variable	Sex	Ages	Distribution
		60 - 69	Log-Normal (mean = ln(0.41), sd = ln(0.44 / 0.41) / 1.96)
		70 - 74	Log-Normal (mean = ln(0.48), sd = ln(0.51 / 0.48) / 1.96)
		80 - 84	Log-Normal (mean = ln(0.68), sd = ln(0.75 / 0.68) / 1.96)
	Women	30 - 49	Log-Normal (mean = ln(0.41), sd = ln(0.49 / 0.41) / 1.96)
		50 - 59	Log-Normal (mean = ln(0.45), sd = ln(0.5 / 0.45) / 1.96)
		60 - 69	Log-Normal (mean = ln(0.47), sd = ln(0.51 / 0.47) / 1.96)
		70 - 74	Log-Normal (mean = ln(0.53), sd = ln(0.56 / 0.53) / 1.96)
		80 - 84	Log-Normal (mean = ln(0.65), sd = ln(0.71 / 0.65) / 1.96)
TC[72 figure 3]	Both	40 - 49	Log-Normal (mean = ln(0.87), sd = ln(1 / 0.87) / 1.96)
		50 - 59	Log-Normal (mean = ln(0.91), sd = ln(0.97 / 0.91) / 1.96)
		60 - 69	Log-Normal (mean = ln(0.93), sd = ln(0.97 / 0.93) / 1.96)
BMI[73 table 1 and figure 2]	Both	30 - 59	Log-Normal (mean = ln(1.18), sd = ln(1.26 / 1.18) / 1.96)
		60 - 69	Log-Normal (mean = ln(1.08), sd = ln(1.15 / 1.08) / 1.96)
Diabetes[74 figure 2]	Both	40 - 59	Log-Normal (mean = ln(3.74), sd = ln(4.58/ 3.74) / 1.96)
		60 - 69	Log-Normal (mean = ln(2.06), sd = ln(2.58/ 2.06) / 1.96)
		70 - 84	Log-Normal (mean = ln(1.8), sd = ln(2.27/ 1.8) / 1.96)
PA[75 table 10.20]	Both	30 - 69	No active days: Log-Normal (mean = ln(1.53), sd = ln(1.79/ 1.53 / 1.96)

Variable	Sex	Ages	Distribution
		70 - 79	No active days: Log-Normal (mean = ln(1.38), sd = ln(1.6/ 1.38) / 1.96)
		80 - 84	No active days: Log-Normal (mean = ln(1.24), sd = ln(1.45/ 1.24) / 1.96)
F&V[78]			Log-Normal (mean = ln(0.95), sd = ln(0.97/ 0.95) / 1.96)
**Universal screening and variations**			
Proportion of participants with a QRISK2 score between 10% and 20%[79]	Both	40 - 74	PERT (min = 0.2, mode = 0.25, max = 0.3, shape = 4)
Proportion of participants with a QRISK2 score higher than 20%[79]	Both	40 - 74	PERT (min = 0.04, mode = 0.05, max = 0.1, shape = 4)
Atorvastatin 20mg relative reduction on TC[56,57]	Both	40 - 74	Normal (mean = 0.32, sd = 0.14)
Atorvastatin prescription uptake[58] (QRISK2: 10% - 20%)	Both	40 - 74	PERT (min = 0.07, mode = 0.17, max = 0.24, shape = 4)
Atorvastatin prescription uptake[58] (QRISK2: > 20%)	Both	40 - 74	PERT (min = 0.2, mode = 0.24, max = 0.3, shape = 4)
Antihypertensive medication prescription uptake[58] (QRISK2: 10% - 20%)	Both	40 - 74	PERT (min = 0.05, mode = 0.13, max = 0.2, shape = 4)
Antihypertensive medication prescription uptake[58] (QRISK2: > 20%)	Both	40 - 74	PERT (min = 0.15, mode = 0.23, max = 0.3, shape = 4)
Persistence with medication[80]	Both	40 - 74	PERT (min = 0.5, mode = 0.8, max = 1, shape = 4)
Adherence to medication[80]	Both	40 - 74	PERT (min = 0.3, mode = 0.7, max = 1, shape = 4)
1st year smoking cessation success rate[81,82]	Both	40 - 74	Bernoulli (probability = 0.1)

Variable	Sex	Ages	Distribution
Relative BMI reduction	Both	40 - 74	1 - PERT (min = 0.97, mode = 0.99, max = 1, shape = 4)
Proportion of high-risk participants to increase F&V consumption by a portion per day	Both	40 - 74	Bernoulli (probability = 0.5)
Proportion of high-risk participants to increase PA by a day per week	Both	40 - 74	Bernoulli (probability = 0.5)
**Universal screening (20% treatment threshold)**			
Atorvastatin prescription uptake[58] (QRISK2: 10% - 20%)	Both	40 - 74	PERT (min = 0.01, mode = 0.07, max = 0.10, shape = 4)
**Population-wide intervention**			
Smoking prevalence relative reduction[83]	Both	30 - 84	PERT (min = 0.05, mode = 0.13, max = 0.14, shape = 4)
BMI rate of increase relative reduction[84–86]	Both	30 - 84	1 - PERT (min = 0.98, mode = 0.99, max = 1, shape = 4)[*]
SBP absolute decrease (mmHg)[87,88]	Both	30 - 84	PERT (min = 0.18, mode = 0.81, max = 1.10, shape = 4)
Proportion of the population to increase their F&V consumption by 1 portion[89,90]	Both	30 - 84	PERT (min = 0.2, mode = 0.5, max = 0.8, shape = 4)

[*] The reduction is applied for five consecutive year so the overall reduction is the distribution to the fifth power.

REFERENCES

1. Williamson P. The Role of the International Journal of Microsimulation. Int J Microsimulation 2007;1:1–2.

2. Zucchelli E, Jones AM, Rice N. The evaluation of health policies through dynamic microsimulation methods. Int J Microsimulation 2012;5:2–20.

3. Department for Communities and Local Government. English indices of deprivation 2010 - Publications - GOV.UK [Internet]. 2011 [cited 2014 Aug 26];Available from: https://www.gov.uk/government/statistics/english-indices-of-deprivation-2010

4. R Core Team. R: a language and environment for statistical computing [Internet]. R Foundation for Statistical Computing; 2014. Available from: http://www.R-project.org/

5. Dowle M, Short T, Lianoglou S, Srinivasan A. data.table: Extension of data.frame [Internet]. 2015. Available from: https://github.com/Rdatatable/data.table/

6. Revolution Analytics, Weston S. foreach: Foreach looping construct for R [Internet]. 2014. Available from: http://CRAN.R-project.org/package=foreach

7. Gaujoux R. doRNG: Generic Reproducible Parallel Backend for foreach Loops [Internet]. 2014. Available from: http://CRAN.R-project.org/package=doRNG

8. L'Ecuyer P. Good Parameters and Implementations for Combined Multiple Recursive Random Number Generators. Oper Res 1999;47:159–64.

9. Alfons A, Kraft S, Templ M, Filzmoser P. Simulation of close-to-reality population data for household surveys with application to EU-SILC. Stat Methods Appl 2011;20:383–407.

10. Dahlgren G, Whitehead M. Policies and strategies to promote social equity in health [Internet]. Copenhagen: World Health Organisation; 1992 [cited 2015 May 5]. Available from: http://core.ac.uk/download/pdf/6472456.pdf

11. Boniface S, Bridges S, Craig R, Darton R, Fuller E, Hancock R, et al. Health Survey for England 2011. Methods and documentation [Internet]. Health and Social Care Information Centre; 2012. Available from: http://www.hscic.gov.uk/catalogue/PUB09300/HSE2011-Methods-and-docs.pdf

12. NatCen Social Research, University College London. Department of Epidemiology and Public Health. Health Survey for England, 2011 [computer file]. Colchester, Essex: UK Data Archive [distributor] [Internet]. 2013 [cited 2014 May 1];Available from: http://dx.doi.org/10.5255/UKDA-SN-7260-1

13. Alfons A, Kraft S. simPopulation: simulation of synthetic populations for surveys based on sample data [Internet]. 2013. Available from: http://CRAN.R-project.org/package=simPopulation

14. Giles Horsfield SSD. Chapter 3: Equivalised Income [Internet]. Off. Natl. Stat.2012 [cited 2015 May 4];Available from: http://www.ons.gov.uk/ons/rel/family-spending/family-spending/family-spending-2012-edition/art-chapter-3--equivalised-income.html

15. Economic Labour and Social Analysis Office for National Statistics. SOC2010 volume 3: the National Statistics socio-economic classification (NS-SEC rebased on the SOC2010) [Internet]. Off.

Natl. Stat.2010 [cited 2015 Jan 6];Available from: http://www.ons.gov.uk/ons/guide-method/classifications/current-standard-classifications/soc2010/soc2010-volume-3-ns-sec--rebased-on-soc2010--user-manual/index.html

16. Mulrow CD, Chiquette E, Angel L, Cornell J, Summerbell C, Anagnostelis B, et al. Dieting to reduce body weight for controlling hypertension in adults. Cochrane Database Syst Rev 2000;CD000484.

17. Goldstein DJ. Beneficial health effects of modest weight loss. Int J Obes Relat Metab Disord J Int Assoc Study Obes 1992;16:397–415.

18. MacMahon S, Cutler J, Brittain E, Higgins M. Obesity and hypertension: epidemiological and clinical issues. Eur Heart J 1987;8 Suppl B:57–70.

19. Dyer AR, Elliott P. The INTERSALT study: relations of body mass index to blood pressure. INTERSALT Co-operative Research Group. J Hum Hypertens 1989;3:299–308.

20. Dattilo AM, Kris-Etherton PM. Effects of weight reduction on blood lipids and lipoproteins: a meta-analysis. Am J Clin Nutr 1992;56:320–8.

21. Kahn SE, Hull RL, Utzschneider KM. Mechanisms linking obesity to insulin resistance and type 2 diabetes. Nature 2006;444:840–6.

22. Lahti-Koski M, Pietinen P, Heliövaara M, Vartiainen E. Associations of body mass index and obesity with physical activity, food choices, alcohol intake, and smoking in the 1982–1997 FINRISK Studies. Am J Clin Nutr 2002;75:809–17.

23. DiPietro L. Physical activity in the prevention of obesity: current evidence and research issues. Med Sci Sports Exerc 1999;31:S542–6.

24. Fogelholm M, Kukkonen-Harjula K. Does physical activity prevent weight gain--a systematic review. Obes Rev Off J Int Assoc Study Obes 2000;1:95–111.

25. Dochi M, Sakata K, Oishi M, Tanaka K, Kobayashi E, Suwazono Y. Smoking as an independent risk factor for hypertension: a 14-year longitudinal study in male Japanese workers. Tohoku J Exp Med 2009;217:37–43.

26. Bowman TS, Gaziano JM, Buring JE, Sesso HD. A Prospective Study of Cigarette Smoking and Risk of Incident Hypertension in Women. J Am Coll Cardiol 2007;50:2085–92.

27. National Centre for Social Research, University College London. Department of Epidemiology and Public Health. Health Survey for England, 2001 [computer file]. 3rd Edition. Colchester, Essex: UK Data Archive [distributor] [Internet]. 2010 [cited 2014 May 1];Available from: http://dx.doi.org/10.5255/UKDA-SN-4628-1

28. National Centre for Social Research, University College London. Department of Epidemiology and Public Health. Health Survey for England, 2002 [computer file]. 2nd Edition. Colchester, Essex: UK Data Archive [distributor] [Internet]. 2010 [cited 2014 May 1];Available from: http://dx.doi.org/10.5255/UKDA-SN-4912-1

29. National Centre for Social Research, University College London. Department of Epidemiology and Public Health. Health Survey for England, 2003 [computer file]. 2nd Edition. Colchester, Essex: UK Data Archive [distributor] [Internet]. 2010 [cited 2014 May 1];Available from: http://dx.doi.org/10.5255/UKDA-SN-5098-1

30. National Centre for Social Research, University College London. Department of Epidemiology and Public Health. Health Survey for England, 2004 [computer file]. 2nd Edition. Colchester, Essex: UK Data Archive [distributor] [Internet]. 2010 [cited 2014 May 1];Available from: http://dx.doi.org/10.5255/UKDA-SN-5439-1

31. National Centre for Social Research, University College London. Department of Epidemiology and Public Health. Health Survey for England, 2005 [computer file]. 3rd Edition. Colchester, Essex: UK Data Archive [distributor] [Internet]. 2011 [cited 2014 May 1];Available from: http://dx.doi.org/10.5255/UKDA-SN-5675-1

32. National Centre for Social Research, University College London. Department of Epidemiology and Public Health. Health Survey for England, 2006 [computer file]. 4th Edition. Colchester, Essex: UK Data Archive [distributor] [Internet]. 2011 [cited 2014 May 1];Available from: http://dx.doi.org/10.5255/UKDA-SN-5809-1

33. National Centre for Social Research, University College London. Department of Epidemiology and Public Health. Health Survey for England, 2007 [computer file]. 2nd Edition. Colchester, Essex: UK Data Archive [distributor] [Internet]. 2010 [cited 2014 May 1];Available from: http://dx.doi.org/10.5255/UKDA-SN-6112-1

34. National Centre for Social Research, University College London. Department of Epidemiology and Public Health. Health Survey for England, 2008 [computer file]. 4th Edition. Colchester, Essex: UK Data Archive [distributor] [Internet]. 2013 [cited 2014 May 1];Available from: http://dx.doi.org/10.5255/UKDA-SN-6397-2

35. National Centre for Social Research, University College London. Department of Epidemiology and Public Health. Health Survey for England, 2009 [computer file]. 2nd Edition. Colchester, Essex: UK Data Archive [distributor] [Internet]. 2011 [cited 2014 May 1];Available from: http://dx.doi.org/10.5255/UKDA-SN-6732-1

36. NatCen Social Research, Free Royal, University College London. Department of Epidemiology and Public Health. Health Survey for England, 2010 [computer file]. 2nd Edition. Colchester, Essex: UK Data Archive [distributor] [Internet]. 2012 [cited 2014 May 1];Available from: http://dx.doi.org/10.5255/UKDA-SN-6986-2

37. NatCen Social Research, University College London. Department of Epidemiology and Public Health. Health Survey for England, 2012 [computer file]. Colchester, Essex: UK Data Archive [distributor] [Internet]. 2014 [cited 2014 May 1];Available from: http://dx.doi.org/10.5255/UKDA-SN-7480-1

38. Hippisley-Cox J, Coupland C, Robson J, Sheikh A, Brindle P. Predicting risk of type 2 diabetes in England and Wales: prospective derivation and validation of QDScore. BMJ 2009;338:b880.

39. Capewell S, O'Flaherty M. Rapid mortality falls after risk-factor changes in populations. The Lancet 2011;378:752–3.

40. Capewell S, O'Flaherty M. Can dietary changes rapidly decrease cardiovascular mortality rates? Eur Heart J 2011;32:1187–9.

41. Comparative quantification of health risks [Internet]. Geneva: World Health Organisation; 2004. Available from: http://www.who.int/publications/cra/en/

42. National Population Projections, 2012-based Statistical Bulletin [Internet]. Office for National Statistics; 2013 [cited 2014 Nov 11]. Available from: http://www.ons.gov.uk/ons/rel/npp/national-population-projections/2012-based-projections/index.html

43. Levin ML. The occurrence of lung cancer in man. Acta - Unio Int Contra Cancrum 1953;9:531–41.

44. Office for National Statistics. Published ad hoc data: health, requests during December 2014 [Internet]. Publ. Ad Hoc Data Health Req. Dec. 20142014 [cited 2015 Oct 14];Available from: http://www.ons.gov.uk/ons/about-ons/business-transparency/freedom-of-information/what-can-i-request/published-ad-hoc-data/health/december-2014/number-of-registered-deaths-by-sex--cause--year--the-adjusted-index.xls

45. Townsend N, Wickramasinghe K, Bhatnagar P, Smolina K, Nichols M, Leal J, et al. Coronary heart disease statistics 2012 edition [Internet]. British Heart Foundation; 2012. Available from: http://www.bhf.org.uk/publications/view-publication.aspx?ps=1002097

46. Smolina K, Wright FL, Rayner M, Goldacre MJ. Incidence and 30-day case fatality for acute myocardial infarction in England in 2010: national-linked database study. Eur J Public Health 2012;22:848–53.

47. Barendregt JJ, van Oortmarssen GJ, Vos T, Murray CJ. A generic model for the assessment of disease epidemiology: the computational basis of DisMod II. Popul Health Metr 2003;1:4.

48. Lim SS, Vos T, Flaxman AD, Danaei G, Shibuya K, Adair-Rohani H, et al. A comparative risk assessment of burden of disease and injury attributable to 67 risk factors and risk factor clusters in 21 regions, 1990–2010: a systematic analysis for the Global Burden of Disease Study 2010. The Lancet 2012;380:2224–60.

49. Boshuizen HC, Lhachimi SK, Baal PHM van, Hoogenveen RT, Smit HA, Mackenbach JP, et al. The DYNAMO-HIA Model: An Efficient Implementation of a Risk Factor/Chronic Disease Markov Model for Use in Health Impact Assessment (HIA). Demography 2012;49:1259–83.

50. Hyndman RJ. demography: Forecasting mortality, fertility, migration and population data [Internet]. 2014. Available from: http://CRAN.R-project.org/package=demography

51. Hyndman RJ, Shahid Ullah M. Robust forecasting of mortality and fertility rates: A functional data approach. Comput Stat Data Anal 2007;51:4942–56.

52. Doll R, Peto R, Boreham J, Sutherland I. Mortality in relation to smoking: 50 years' observations on male British doctors. BMJ 2004;328:1519.

53. The DECODE Study Group. Is the current definition for diabetes relevant to mortality risk from all causes and cardiovascular and noncardiovascular diseases? Diabetes Care 2003;26:688–96.

54. Collins GS, Altman DG. Predicting the 10 year risk of cardiovascular disease in the United Kingdom: independent and external validation of an updated version of QRISK2. BMJ 2012;344:e4181.

55. Symmons D, Turner G, Webb R, Asten P, Barrett E, Lunt M, et al. The prevalence of rheumatoid arthritis in the United Kingdom: new estimates for a new century. Rheumatology 2002;41:793–800.

56. Law MR, Wald NJ, Rudnicka AR. Quantifying effect of statins on low density lipoprotein cholesterol, ischaemic heart disease, and stroke: systematic review and meta-analysis. BMJ 2003;326:1423.

57. Edwards JE, Moore RA. Statins in hypercholesterolaemia: A dose-specific meta-analysis of lipid changes in randomised, double blind trials. BMC Fam Pract 2003;4:18.

58. Forster AS, Dodhia H, Booth H, Dregan A, Fuller F, Miller J, et al. Estimating the yield of NHS Health Checks in England: a population-based cohort study. J Public Health 2014;fdu079.

59. Usher-Smith JA, Pritchard J, Poole S, Griffin SJ. Offering statins to a population attending health checks with a 10-year cardiovascular disease risk between 10% and 20%. Int J Clin Pract 2015;n/a – n/a.

60. Koerkamp BG, Stijnen T, Weinstein MC, Hunink MGM. The combined analysis of uncertainty and patient heterogeneity in medical decision models. Med Decis Making 2011;31:650–61.

61. Briggs AH, Weinstein MC, Fenwick EAL, Karnon J, Sculpher MJ, Paltiel AD. Model parameter estimation and uncertainty: a report of the ISPOR-SMDM modeling good research practices Task Force-6. Value Health 2012;15:835–42.

62. Mackenbach JP, Kunst AE. Measuring the magnitude of socio-economic inequalities in health: An overview of available measures illustrated with two examples from Europe. Soc Sci Med 1997;44:757–71.

63. Bross IDJ. How to Use Ridit Analysis. Biometrics 1958;14:18–38.

64. Regidor E. Measures of health inequalities: part 2. J Epidemiol Community Health 2004;58:900–3.

65. Schmid VJ, Held L. Bayesian age-period-cohort modeling and prediction-BAMP. J Stat Softw 2007;21:1–15.

66. Friendly M. Mosaic Displays for Multi-Way Contingency Tables. J Am Stat Assoc 1994;89:190–200.

67. Allen K, Gillespie DOS, Guzman-Castillo M, Diggle PJ, Capewell S, O'Flaherty M. Future trends and inequalities in premature coronary deaths in England: Modelling study. Int J Cardiol 2016;203:290–7.

68. Ezzati M, Henley SJ, Thun MJ, Lopez AD. Role of smoking in global and regional cardiovascular mortality. Circulation 2005;112:489–97.

69. Huxley RR, Woodward M. Cigarette smoking as a risk factor for coronary heart disease in women compared with men: a systematic review and meta-analysis of prospective cohort studies. The Lancet 2011;378:1297–305.

70. He J, Vupputuri S, Allen K, Prerost MR, Hughes J, Whelton PK. Passive Smoking and the Risk of Coronary Heart Disease — A Meta-Analysis of Epidemiologic Studies. N Engl J Med 1999;340:920–6.

71. Prospective Studies Collaboration. Age-specific relevance of usual blood pressure to vascular mortality: a meta-analysis of individual data for one million adults in 61 prospective studies. The Lancet 2002;360:1903–13.

72. Prospective Studies Collaboration. Blood cholesterol and vascular mortality by age, sex, and blood pressure: a meta-analysis of individual data from 61 prospective studies with 55 000 vascular deaths. The Lancet 2007;370:1829–39.

73. The Emerging Risk Factors Collaboration. Separate and combined associations of body-mass index and abdominal adiposity with cardiovascular disease: collaborative analysis of 58 prospective studies. The Lancet 2011;377:1085–95.

74. The Emerging Risk Factors Collaboration. Diabetes mellitus, fasting blood glucose concentration, and risk of vascular disease: a collaborative meta-analysis of 102 prospective studies. The Lancet 2010;375:2215–22.

75. Bull FC, Armstrong TP, Dixon T, Ham S, Neiman A, Pratt M. Comparative quantification of health risks. Chapter 10: physical inactivity [Internet]. Geneva: World Health Organisation; 2004. Available from: http://www.who.int/publications/cra/en/

76. Dauchet L, Amouyel P, Hercberg S, Dallongeville J. Fruit and vegetable consumption and risk of coronary heart disease: a meta-analysis of cohort studies. J Nutr 2006;136:2588–93.

77. Oono IP, Mackay DF, Pell JP. Meta-analysis of the association between secondhand smoke exposure and stroke. J Public Health 2011;33:496–502.

78. Dauchet L, Amouyel P, Dallongeville J. Fruit and vegetable consumption and risk of stroke A meta-analysis of cohort studies. Neurology 2005;65:1193–7.

79. Chang KC-M, Soljak M, Lee JT, Woringer M, Johnston D, Khunti K, et al. Coverage of a national cardiovascular risk assessment and management programme (NHS Health Check): Retrospective database study. Prev Med 2015;78:1–8.

80. Wallach-Kildemoes H, Andersen M, Diderichsen F, Lange T. Adherence to preventive statin therapy according to socioeconomic position. Eur J Clin Pharmacol 2013;69:1553–63.

81. Bauld L, Bell K, McCullough L, Richardson L, Greaves L. The effectiveness of NHS smoking cessation services: a systematic review. J Public Health 2010;32:71–82.

82. West R, Lesley Owen. Estimates of 52-week continuous abstinence rates following selected smoking cessation interventions in England [Internet]. Smoking in England; 2012. Available from: http://www.smokinginengland.info/downloadfile/?type=report&src=5

83. Levy DT, Huang A-T, Currie LM, Clancy L. The benefits from complying with the framework convention on tobacco control: a SimSmoke analysis of 15 European nations. Health Policy Plan 2014;29:1031–42.

84. Sharma A, Hauck K, Hollingsworth B, Siciliani L. The effects of taxing sugar-sweetened beverages across different income groups. Health Econ 2014;23:1159–84.

85. Briggs ADM, Mytton OT, Kehlbacher A, Tiffin R, Rayner M, Scarborough P. Overall and income specific effect on prevalence of overweight and obesity of 20% sugar sweetened drink tax in UK: econometric and comparative risk assessment modelling study. BMJ 2013;347:f6189.

86. Cabrera Escobar MA, Veerman JL, Tollman SM, Bertram MY, Hofman KJ. Evidence that a tax on sugar sweetened beverages reduces the obesity rate: a meta-analysis. BMC Public Health 2013;13:1072.

87. Collins M, Mason H, O'Flaherty M, Guzman-Castillo M, Critchley J, Capewell S. An economic evaluation of salt reduction policies to reduce coronary heart disease in England: a policy modeling study. Value Health J Int Soc Pharmacoeconomics Outcomes Res 2014;17:517–24.

88. Gillespie DOS, Allen K, Guzman-Castillo M, Bandosz P, Moreira P, McGill R, et al. The health equity and effectiveness of policy options to reduce dietary salt intake in England: policy forecast. PLoS ONE 2015;10:e0127927.

89. Bartlett S, Klerman J, Olsha L, Logan C, Blocklin M, Beauregard M, et al. Evaluation of the Healthy Incentives Pilot (HIP) Final Report [Internet]. 2014. Available from: http://www.fns.usda.gov/sites/default/files/HIP-Final.pdf

90. Nnoaham KE, Sacks G, Rayner M, Mytton O, Gray A. Modelling income group differences in the health and economic impacts of targeted food taxes and subsidies. Int J Epidemiol 2009;38:1324–33.
